# Constraint of Lignin–Carbohydrate Complex Orchestrated on Polyphenol in Oil–Water Interface Targeting Ulcerative Colitis Therapy

**DOI:** 10.1002/advs.202524070

**Published:** 2026-03-12

**Authors:** Qian Wu, Xingyu Zhang, Jingjia Zhang, Gaohui Huang, Chen Zhou, Chunlin Li, Xiaojun Huang, Jianbo Xiao, Nianjie Feng, Yuanbin She

**Affiliations:** ^1^ National “111” Center for Cellular Regulation and Molecular Pharmaceutics Hubei University of Technology Wuhan Hubei China; ^2^ State Key Laboratory of Green Chemical Synthesis and Conversion College of Chemical Engineering Zhejing University of Technology Hangzhou Zhejiang China; ^3^ State Key Laboratory for Quality and Safety of Agro–Products Institute of Agro–Products Safety and Nutrition Zhejiang Academy of Agricultural Sciences Hangzhou Zhejiang China; ^4^ Research Group on Food Nutritional Biochemistry and Health Universidad Europea del Atlántico Santander Spain; ^5^ State Key Laboratory of Food Science and Resources China–Canada Joint Lab of Food Science and Technology (Nanchang) Nanchang University Nanchang Jiangxi China

**Keywords:** intestinal microflora, lignin–carbohydrate complex, polyphenols, ulcerative colitis, W_1_/O/W_2_ emulsion

## Abstract

The therapeutic potential of polyphenols in ulcerative colitis (UC), mediated through immune modulation and gut microbiota homeostasis. To enhance the oral bioavailability of polyphenols, we architected a colon–targeted W_1_/O/W_2_ emulsion system featuring a rationally designed lignin–carbohydrate complex (LCC) as a dual–functional emulsifier system for the first time. Based on the innate structural duality of LCC, which comprising hydrophobic lignin and hydrophilic carbohydrates, we employed LCC for O/W emulsifier. This inherent amphiphilicity was further engineered via laccase–mediated grafting of isovanillin, yielding a modified LCC with tailored lipophilicity for effective W/O interfacial stabilization. The W_1_/O/W_2_ emulsion ensured the stability of the encapsulated polyphenols with divergent polarity but also enabled pH–responsive payload release under colonic conditions (pH >7.0). In DSS–induced colitis, the system demonstrated a synergistic effect, the LCC itself acted as a prebiotic to modulate the gut microbiota, specifically enriching short chain fatty acid–producing bacteria, while the released polyphenols reinforced the intestinal barrier, which collectively accelerated mucosal healing. This research proposes a carbon–neutral therapeutic strategy for colitis, not only establishing a proof–of–concept for replacing synthetic emulsifiers with engineered biomass, but also as a multi–functional platform to stabilize colon–targeted co–delivery system and microbiome regulation in colitis.

## Graphical abstract

This study constructed the W_1_/O/W_2_ emulsion–based targeted therapy delivery system for ulcerative colitis (UC) utilizing LCC as surfactant for the first time. This multifunctional emulsion offered certain therapeutic advantages for UC, including targeted colonic delivery of active compounds, synergistic modulation of gut microbiota through combined action of polyphenols and LCC prebiotics and comprehensive restoration of the intestinal barrier in DSS–induced colitis.

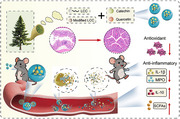

## 1 Introduction

Inflammatory bowel diseases (IBD), primarily encompassing Crohn's disease (CD) and ulcerative colitis (UC) [1, 2]. UC is typically characterized by symptoms such as weight loss and rectal bleeding [3, 4]. Studies have shown that dysregulation of the intestinal mucosal immune response and gut microbiota imbalance are closely associated with the onset and progression of UC [5, 6]. At present, the intestinal immune response is mainly regulated by steroids, immunosuppressants, but long–term use may trigger serious complications [7]. Therefore, the exploration of safer and more effective treatments has become a research hotspot today.

Polyphenols have demonstrated potential therapeutic applications in the treatment of UC. Catechin and Quercetin are widely distributed in plants and exhibit a certain degree of synergistic activity [8, 9, 10, 11, 12, 13, 14]. Nevertheless, their clinical translation is significantly constrained by intrinsic limitations, including poor stability, low aqueous solubility, and limited oral bioavailability. Recent studies [15] have shown that W_1_/O/W_2_ emulsion can effectively encapsulate bioactive compounds and improve their oral bioavailability, showing great potential for application in biomedicine. Furthermore, compared to conventional single–layer and Pickering emulsions, W_1_/O/W_2_ emulsions offer enhanced modulation of oil–water interfaces, while their multiphasic structure confers unique advantages for the co–delivery of both hydrophilic and hydrophobic bioactive compounds. Specifically, conventional single–layer emulsions are stabilized by surfactants, whereas Pickering emulsions are stabilized by solid particles at the interface. In contrast, W_1_/O/W_2_ emulsions are not confined to solid–particle stabilization, they benefit from greater versatility in interfacial design. However, the fabrication of W_1_/O/W_2_ emulsions typically require multiple emulsifiers and rely heavily on chemically synthesized surfactants such as Tween 80 and PGPR, which may impart undesirable odors and raise safety concerns.

The increasingly stringent global carbon–neutrality goals and the advancement of green–energy policies have accelerated the exploration of sustainable and renewable natural functional materials [16, 17]. Lignin–carbohydrate complex (LCC), a biocompatible and nontoxic biopolymer [18], consists of a hydrophobic lignin backbone covalently linked to hydrophilic carbohydrate moieties, which endow LCC with pronounced amphiphilicity. Tsuji [19] demonstrated that the immunostimulatory activity of LCC is likely mediated by the enrichment of galactose and mannose residues. Moreover, compared to conventional synthetic emulsifiers, LCC not only effectively inhibits HIV proliferation and *Escherichia coli* growth, but also promotes the metabolic activity of hepatocytes [20, 21, 22, 23]. Additionally, the lignin fraction of LCC contains abundant phenolic hydroxyl groups, conferring antioxidant capability in free–radical scavenging [21, 24, 25], whereas its polysaccharide components may function as potential prebiotics for the gut microbiota. Collectively, these multifaceted properties make LCC as a highly attractive natural platform for the design of next–generation sustainable surfactants.

Here, we reported the first successful construction of LCC–stabilized W_1_/O/W_2_ emulsion–based targeted therapy delivery system for UC. Building upon the O/W emulsifying properties of LCC, we enzymatically grafted isoeugenol onto the lignin moiety using laccase, yielding a modified LCC that functions as a W/O emulsifier. Considering the complementary anti–inflammatory and antioxidant activities of Catechin and Quercetin [13, 26, 27, 28, 29] along with the prebiotic potential of LCC, we hypothesized that LCC synergizes with Catechin and Quercetin to exert therapeutic effects against UC, and developed an LCC–stabilized W_1_/O/W_2_ emulsion system capable of co–encapsulating polyphenols with disparate polarities and achieving colon–specific controlled release, which was validated through mass spectrometry and in vitro imaging.

**SCHEME 1:**
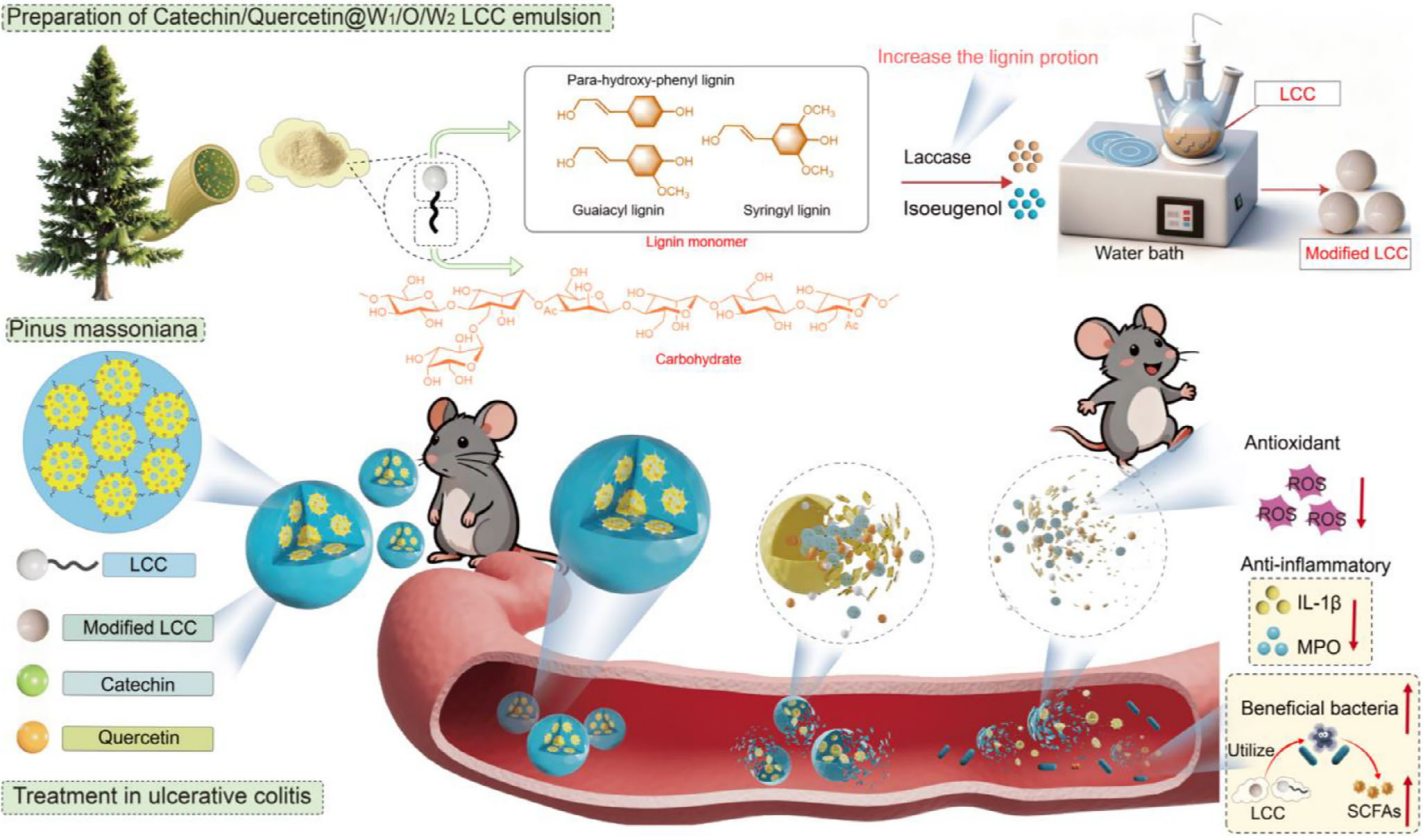
Preparation of Catechin/Quercetin@W_1_/O/W_2_ LCC emulsion and regulation of gut microbiota in ulcerative colitis mice.

This multifunctional emulsion system offered several therapeutic advantages for UC, including targeted colonic delivery of active compounds, synergistic modulation of gut microbiota through combined action of polyphenols and LCC–derived prebiotics and comprehensive restoration of the intestinal barrier in DSS–induced colitis. Our findings not only propel advancements in natural emulsifier design and lignin valorization but also establish a novel therapeutic platform for modulating intestinal homeostasis in ulcerative colitis (Scheme 1).

## 2 Results and Discussion

### 2.1 Chemical Characterization of LCC and Modified LCC

**FIGURE 1:**
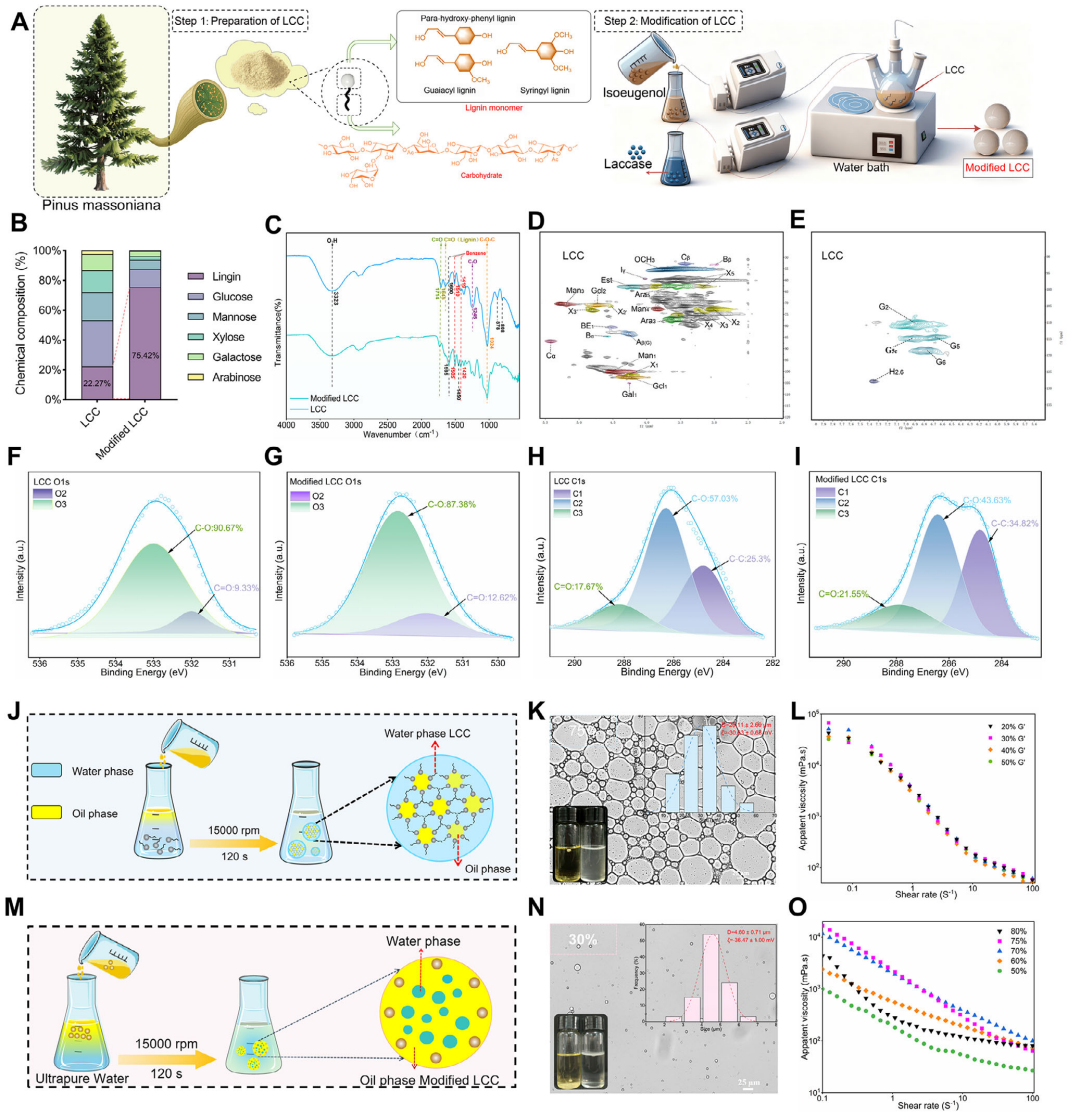
Chemical characterization and emulsion preparation of LCC. (A) Step1: Preparation of LCC, Step2: Modification of LCC. (B) Chemical composition analysis of LCC/modified LCC. (C) Fourier transform infrared spectroscopy of LCC/modified LCC. (D–E) 2D–HSQC NMR spectroscopy of LCC. (F–I) XPS analysis of LCC/modified LCC. (J) Schematic diagram of O/W emulsion preparation. (K) 75% O/W emulsion micrograph, size, Zeta potential. (L) Rheology of O/W emulsion: viscosity. (M) Schematic diagram of W/O emulsion preparation. (N) 30% W/O emulsion micrograph, size, Zeta potential. (O) Rheology of W/O emulsion: viscosity.

The lignin–carbohydrate complexes were extracted from Pinus massoniana (Figure 1A step–1), which consist of hydrophobic lignin covalently linked to hydrophilic carbohydrates. After modification (Figure 1A step–2, Table S1), the average molecular weight of LCC increased from 2.26 ± 0.01 × 10^4^ Da to 4.18 ± 0.02 × 10^4^ Da. Specifically, the compositional analysis revealed that LCC contained a carbohydrate–to–lignin ratio of 77.7% to 22.3%, whereas the modified LCC exhibited a markedly shifted ratio of 24.5% to 75.5% (Figure 1B, Figure S1 and Table S2).

On this basis, to further verify structural alterations, Fourier Transform Infrared Spectroscopy (FTIR) was performed, which showed that the characteristic absorption bands corresponding to lignin aromatic units (1600, 1510, and 1419 cm^−^^1^) were prominently observed and showed increased intensities following modification. In addition, the characteristic absorption peaks of the xylan–ether bond at 1024 cm^−1^, the β–glycosidic bond at 878 cm^−1^, and the mannosidic bond at 808 cm^−1^ were also detected [30], but their peak intensities decreased after modification (Figure 1C). These results collectively confirm successful structural reconstruction of LCC toward a more lignin–enriched architecture. Collectively, these transformations in chemical bonding demonstrate that the grafting of isoeugenol through radical coupling of its aromatic ring onto the LCC backbone provided a critical chemical foundation for improved interfacial anchoring and emulsion stability.

2D–HSQC NMR spectroscopy was employed to further elucidate the chemical structure of LCC (Figure 1D,E, Figure S2, Table S3). The characteristic guaiacyl unit cross–peaks were observed at δ_C_/δ_H_ 110.18/6.90 (G_2_), 114.40/6.68 (G_5_), and 120.30/6.74 (G_6_) in the modified LCC, indicating that these aromatic structures remained relatively stable during the modification process [31, 32, 33]. Notably, the δ_C_/δ_H_ 86.75/5.42 (C_α_) signal disappeared after modification, and the δ_C_/δ_H_ 79.34/4.01 (D’_α_) signal of spiradienone appeared, which may be due to the oxidative cleavage of the C_α_–C_β_ bond and the coupling with guaiacyl units during the modification process results in a more hydrophobic and stable condensation structure, which significantly enhances the interfacial hydrophobicity of LCC. It was worth noting that C_3_–H_3_, C_4_–H_4_ of *β–*D–galactoside, *β–*D–glucopyranosyl (C_1_–H_1_, C_2_–H_2_) and *β*–D–mannose signal peaks were weak in the modified LCC. Meanwhile, several lignin structural signals, particularly *β*–O–4′ and *β*–β′ linkages, increased in intensity to varying degrees, further confirming lignin enrichment and structural rearrangement during modification.

C and O were the main elements on the surface of LCC/modified LCC (Figure S3, Tables S4 and S5). The O/C ratio of the modified LCC decreased to 33%, compared with the LCC (Figure 1F,G), indicating that the successful incorporation of isoeugenol, the changes in surface chemical characteristics likely result from laccase–catalyzed grafting of isoeugenol onto the LCC structure, which leads to the shielding or conversion of hydroxyl groups into hydrophobic moieties, which was led to the enhanced hydrophobicity of modified LCC. In addition, the C—C ratio of the modified LCC increased to 34.82%, and C═O also increased to some extent (Figure 1H,I), which may be due to the oxidation of the hydroxyl group to the carbonyl group in laccase catalysis [34]. This enhanced hydrophobicity allowed the LCC to forming a more stable W_1_/O primary emulsion, which serving as the essential foundation for constructing the entire W_1_/O/W_2_ emulsion. Notably, we found that both native and modified LCC are free from detectable residues of relevant toxic organic solvents (Figure S4), complying with safety standards for natural, green–source emulsifiers and further supporting their practical biosafety.

In summary, the significant increase in lignin content during modification results from the polymerization of exogenous isoeugenol and its covalent integration with the inherent lignin framework, facilitating the formation of a more stable interfacial film at the oil–water interface. The structural analyses indicate that the modification primarily occurs within the lignin results in an asymmetric amphiphilic structure of LCC, which served as the fundamental basis for constructing dual interface (W/O and O/W) green–source emulsions.

### 2.2 Preparation and Characterization of Emulsions Based on LCC and Modified LCC

The preparation methods for the O/W and W/O emulsions are shown in Figure 1J,M. For the O/W emulsions, when the oil phase volume fraction ranged from 50% to 75%, the emulsions appeared milky white (Figure S5) and maintained intact morphology (Figure 1K, Figure S6). After standing for 24 h, the 75% oil phase system exhibited the highest stability. For the W/O emulsions, the 30% internal aqueous phase system showed the best stability after 24 h (Figure S5), during which the number of water droplets was maximal (Figure 1N, Figure S7). In contrast, in the 20% system, the reduced water content resulted in sparse and dispersed droplets that collided more frequently due to intensified Brownian motion, ultimately causing phase separation [35].

For the O/W emulsions, the 75% internal phase system exhibited an average droplet size of 20.10 ± 2.66 µm (Figure 1K). In contrast, the W/O emulsion showed significantly smaller droplets with an average size of 4.60 ± 0.71 µm in 30% internal phase system (Figure 1N). The reduction in droplet size correlated was attributed to the lower interfacial tension provided by the modified LCC in the oil phase, which in turn enhanced emulsion stability. Moreover, the Zeta were consistent with the observed microscopic morphology and droplet size distributions, further confirming the stability trends of the two emulsion systems (Figure S8A,B).

The rheological properties can indicate the network structure and mechanical properties of the sample. In O/W emulsions, the 75% internal phase exhibited the highest G′ and G″ values (Figure 1L and Figure S9A,B), with G′ consistently exceeding G″, indicating a predominantly elastic behavior and strong resistance to deformation [36]. In W/O emulsions, as shown in Figure 1O and Figure S9C,D, the water–oil ratio had little effect on the viscosity and modulus of the emulsion. Overall, the comprehensive characterization of LCC and the resulting emulsions successfully demonstrated that it was feasible to use LCC/modified LCC as emulsifiers for O/W and W/O emulsions.

**FIGURE 2:**
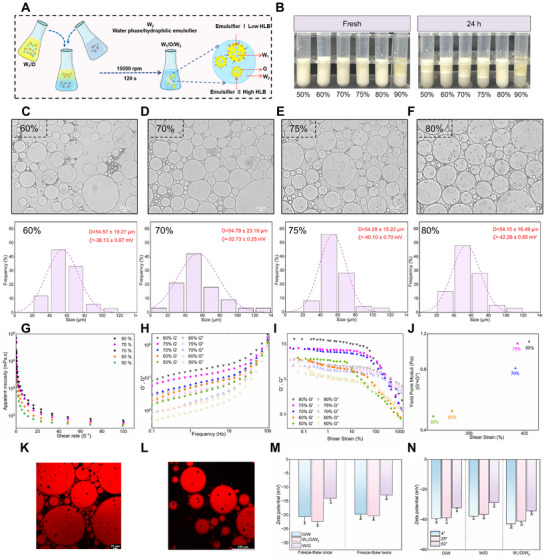
W_1_/O/W_2_ emulsion and its characterization. (A) Schematic diagram of double emulsion preparation. (B) Macrograph of emulsions with 50%–80% internal phase colostrum. (C–F) Size and Zeta potential of W_1_/O/W_2_ emulsions scale bar = 25 µm. (G–J) W_1_/O/W_2_ emulsion rheology: viscosity, modulus, stress–strain. (K–L) 80% W_1_/O/W_2_ emulsion under laser confocal (Nile red stained oil phase) scale bar = 100, 25 µm. (M) Freeze–thaw stability of different types of emulsions. (N) Temperature stability of different types of emulsions. Different letters represent significant differences between the two groups (n = 3).

The bilayer emulsion system, characterized by its unique “two–membrane three–phase” structure, provides enhanced protection of encapsulated bioactive compounds and enables more controlled release compared with conventional emulsions. A green–sourced and more stable W_1_/O/W_2_ emulsion system was successfully fabricated using LCC and modified LCC as stabilizers (Figure 2A, Figure S10). The 80% internal phase group showed the highest stability after 24 h of storage (Figure 2B), likely because its phase volume approached the upper limit for maintaining a coherent emulsion structure. In contrast, when the internal phase volume reached 90%, the interfacial film was insufficient to fully encapsulate the excess oil phase during secondary homogenization^3^ ^4^, ultimately preventing the formation of a stable W_1_/O/W_2_ system.

In the W_1_/O/W_2_ emulsions, the “two–membrane three–phase” structure showed the optimal morphological characteristics under microscopy (Figure 2C–F, Figure S11). Combined with the size and Zeta potential of W_1/_O/W_2_ emulsions, it was found that the 80% group of W_1_/O/W_2_ emulsions was the most stable (Figure S8C). The micron–sized W_1_/O/W_2_ emulsion effectively ensures oral stability and colon–targeted accumulation of Catechin and Quercetin. Its structural configuration was further confirmed by laser confocal microscopy, which revealed a clear “two–membrane three–phase” (W_1_/O/W_2_) architecture (Figure 2K–L), consistent with the characteristic morphology of double emulsions reported in previous studies [37, 38]. As shown in Figure 2G–J, the G' values of all W_1_/O/W_2_ emulsions were higher than the G'' values and the values of G' and G'' were the highest. Notably, both G' and G'' reached their highest levels when the dispersed phase accounted for 80%, which may be due to the larger volume of initial emulsion and the stronger interaction between droplets.

Freeze–thaw cycling tests (Figure 2M) revealed that the W_1_/O/W_2_ emulsion showed significant electrochemical stability. In addition, the W_1_/O/W_2_ emulsion demonstrated good thermal stability, indicating considerable thermal tolerance, which we attributed to the relatively rigid interfacial film formed by LCC (Figure S12). The film effectively prevented droplet coalescence and thereby enhanced the stability of the system against thermal perturbation. Notably, kinetic stability was preserved across pH 2–5 for all emulsion systems. Under weak alkaline conditions (pH 8), the single–layer emulsions underwent structural collapse accompanied by marked droplet reduction, whereas the W_1_/O/W_2_ emulsion progressively transitioned into an O/W morphology, which triggered the gradual disintegration of the double–layered configuration. The rigidity of the LCC–derived interfacial film partially restricted droplet coalescence, thereby facilitating a pH–modulated, stepwise release profile of encapsulated polyphenols (Figure S13). These results indicate that the W_1_/O/W_2_ emulsion provides robust interfacial protection for encapsulated active compounds during gastrointestinal digestion [39].

### 2.3 Characterization of Catechin/Quercetin@W_1_/O/W_2_ LCC Emulsion During Digestion

**FIGURE 3:**
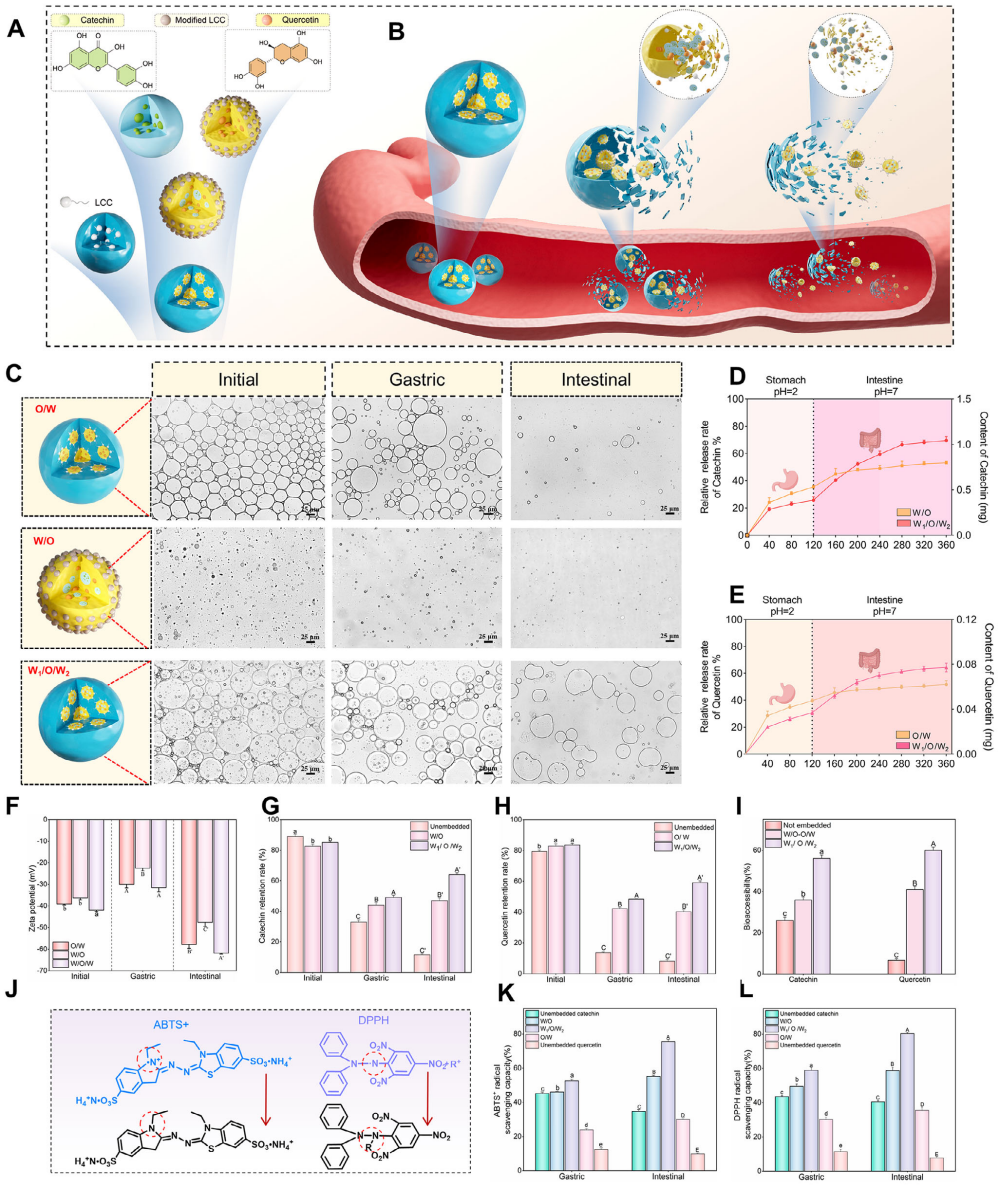
Catechin/Quercetin@W_1_/O/W_2_ LCC emulsion during the digestion. (A) Schematic diagram of Catechin/Quercetin@W_1_/O/W_2_ LCC emulsion preparation. (B) Schematic diagram of W_1_/O/W_2_ LCC emulsion changes during digestion. (C) Micrographs of emulsions in different systems, scale bar = 25 µm. (D,E) Release rates of Catechin and Quercetin. (F) Zeta potential changes of emulsions at different digestion stages. (G–H) Retentions rate of Catechin/Quercetin during digestion. (I) Bio–acceptability of Catechin and Quercetin. (J–L) Antioxidant mechanism of Catechin/Quercetin@W_1_/O/W_2_ LCC emulsion, n = 3.

Based on the above characteristics, a schematic illustration of the emulsions stabilized by LCC and modified LCC was constructed (Figure 3A). The solubility of Catechin in the aqueous phase was measured to be 1.50 mg/mL, whereas Quercetin showed a solubility of 0.12 mg/mL in the soybean oil phase (Figure S14A,B). Moreover, the encapsulation efficiency of Catechin was as high as 96.61 ± 0.57%, while that of Quercetin was 80.83 ± 0.46% (Figure S15), demonstrating superior performance compared to conventional W_1_/O/W_2_ emulsions [40, 41]. To assess whether effective interfacial protection can be provided for the active substances during gastrointestinal delivery of W_1_/O/W_2_ emulsion, a digestion experiment was performed to observe the microstructural changes (Figure 3B,C, Figures S16 and S17). During the gastric phase, the emulsion experienced only slight structural perturbations. However, in the intestinal phase, the combined effects of the alkaline environment, pancreatic enzymes, and bile salts led to partial rupture of the emulsion's interfacial membrane [42].

The release profiles of Catechin and Quercetin from the W_1_/O/W_2_ system during digestion phases were quantified by LC–MS (Figure 3D,E, Figures S18–S20). At the end of the gastric phase, 25.8 ± 2.53% of Catechin was released, increasing to 69.51 ± 2.90% after intestinal digestion. Similarly, Quercetin release reached 30.83 ± 1.61% in the gastric stage and 64.42 ± 3.24% in the intestinal stage. In contrast, the distinct release behavior of the W_1_/O/W_2_ emulsion stems from the differential adsorption of LCC and modified LCC at the O/W and W/O interfaces, respectively, forming interfacial layers with distinct charge characteristics (Figure 3F). As a dietary‑fiber‑based emulsifier, LCC may interact with bile salts in an environment‑dependent manner: bile salt concentrations facilitate the solubilization of the oil phase in LCC emulsions, thereby accelerating interfacial stripping. In addition, LCC may also influence upstream lipid digestion by modulating pancreatic enzyme activity, indirectly altering the functional microenvironment of bile salts [43]. During the gastric acidic phase, the W_1_/O/W_2_ emulsion exhibits resistance to coalescence. In the intestinal phase, digestive components such as pancreatin preferentially disrupt the oil–water interface stabilized by modified LCC, leading to the release of the Quercetin. Following oil–phase digestion, the inner aqueous phase is directly exposed to intestinal fluid, resulting in the subsequent release of the Catechin.

Simultaneously, the retention rates of Catechin/Quercetin during digestive phases were systematically evaluated. The retention rate of Catechin in W_1_/O/W_2_ emulsion was 64.96 ± 1.32%, which was significantly higher than that in W/O emulsion (Figure 3G), while Quercetin retention reached 61.12 ± 1.09% (Figure 3H). The superior retention is attributed to the multilayer structure of the W_1_/O/W_2_ system, which provides additional barriers that impede enzymatic erosion compared with O/W emulsions [44]. Since micellar solubility directly influences the bio–accessibility of active substances, it determines the fraction available for intestinal absorption [45]. After the intestinal digestion, the bio–accessibility of Catechin and Quercetin in the W_1_/O/W_2_ emulsion was 56.12 ± 1.62% and 60.04 ± 1.55%, respectively, which were significantly higher than those in the monolayer emulsion and the free group (Figure 3I).

The radical–scavenging activities of ABTS^+^ and DPPH during gastrointestinal digestion were further evaluated (Figure 3J–L). Compared with the gastric phase, greater amounts of bioactive compounds were released during the intestinal phase due to the combined effects of bile salts and the pH environment. Consequently, the W_1_/O/W_2_ emulsion exhibited significantly enhanced free radical scavenging, with ABTS^+^ and DPPH activities reaching 75.46 ± 1.30% and 80.33 ± 0.50%, respectively, indicating that the W_1_/O/W_2_ system effectively releases Catechin and Quercetin in the intestinal stage, thereby maximizing their antioxidant potential.

### 2.4 In Vivo and In Vitro Safety of Catechin/Quercetin@W_1_/O/W_2_ Emulsion

**FIGURE 4:**
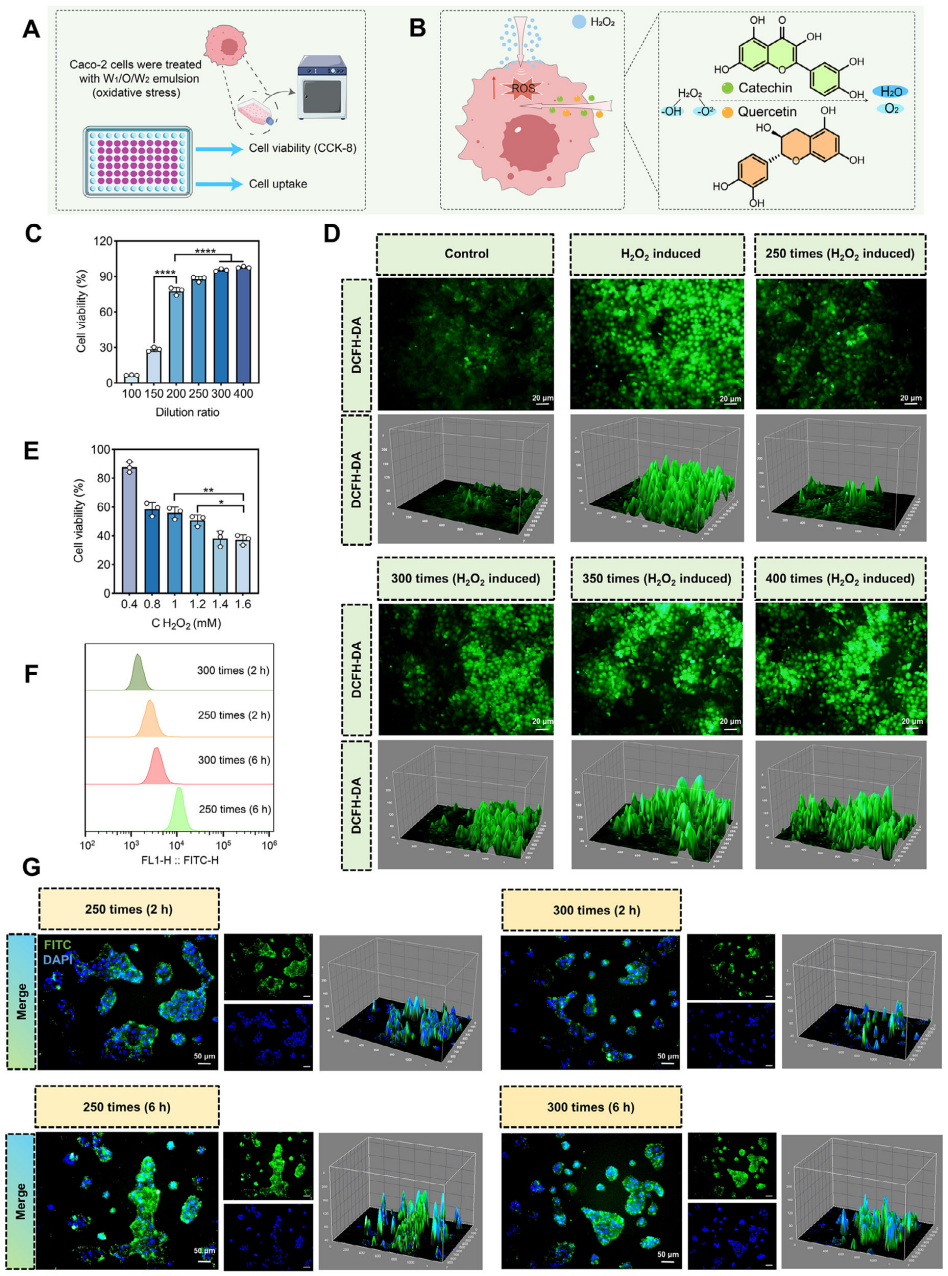
Effect of Catechin/Quercetin@W_1_/O/W_2_ LCC emulsion on Caco–2 cells. (A) Schematic diagram of cell experiment. (B) Antioxidant mechanism of Catechin/Quercetin@W_1_/O/W_2_ LCC emulsion. (C) Effects of Catechin/Quercetin@W_1_/O/W_2_ LCC emulsion on cell viability. (D–E) Effects of Catechin/Quercetin@W_1_/O/W_2_ LCC emulsion on cell oxidative stress, scale bar = 20 µm. (F) Quantitative detection of cellular uptake by flow cytometry using FITC labeling. (G) Fluorescence microscopy of cell uptake, including blue fluorescence (DAPI), green fluorescence (FITC), scale bar = 50 µm. (n = 3) *p < 0.05, **p < 0.01, ***p < 0.001, ****p < 0.0001, n = 3.

The raw emulsion solutions were diluted with culture medium to address potential cytotoxicity concerns, as undiluted emulsion had high concentrations of bile salts that could cause cellular damage through hyperosmotic stress. It was found that the cell survival rate of the deliute 250 times, 300 times, and 400 times groups were more than 80%, and the cell morphology was relatively intact (Figure 4A,C, Figure S21A).

To verify the bioactivity of Catechin and Quercetin released from emulsion, an oxidative stress model was established in Caco–2 cells using H_2_O_2_ (Figure 4B), in which H_2_O_2_ concentration (1.2 mM) with cell viability of about 50% was selected for induction (Figure 4E, Figure S21B). It was found that O_2_^2−^ in H_2_O_2_ was reduced to O^2−^ in H_2_O after the antioxidant effect of Catechin and Quercetin. In Figure 4D, we found that the ROS fluorescence signal of the 250 times group was close to that of the Con group (Figure 4D, Figure S22), indicating that Catechin and Quercetin released from W_1_/O/W_2_ emulsion have sufficient bioactivity to mitigate oxidative stress.

Within incubation of 2 to 6 h, the fluorescence intensity associated with cellular internalization was observed to increase over time (Figure 4G, Figures S23–S25). Quantitative analysis of FITC by flow cytometry demonstrated that the highest fluorescence intensity was achieved after 6 h of treatment with the 250 times group (Figure 4F, Figure S26), indicating that prolonged exposure of the Catechin/Quercetin@W_1_/O/W_2_ LCC emulsion enables effective absorption by Caco‑2 cells. Moreover, the 250 times group was found to effectively modulate intracellular inflammatory levels: upregulating IL–10 (Figure S27A) and downregulating TNF–α and IL–1β (Figure S27B,C). Additionally, the biocompatibility of the W_1_/O/W_2_ system was further evaluated using a hemolysis assay. The supernatant color of the emulsion–treated samples closely resembled that of the PBS control group (Figure S28A–C), confirming negligible hemolytic activity.

### 2.5 Colon Targeted Delivery of Catechin/Quercetin@W_1_/O/W_2_ LCC Emulsion

**FIGURE 5:**
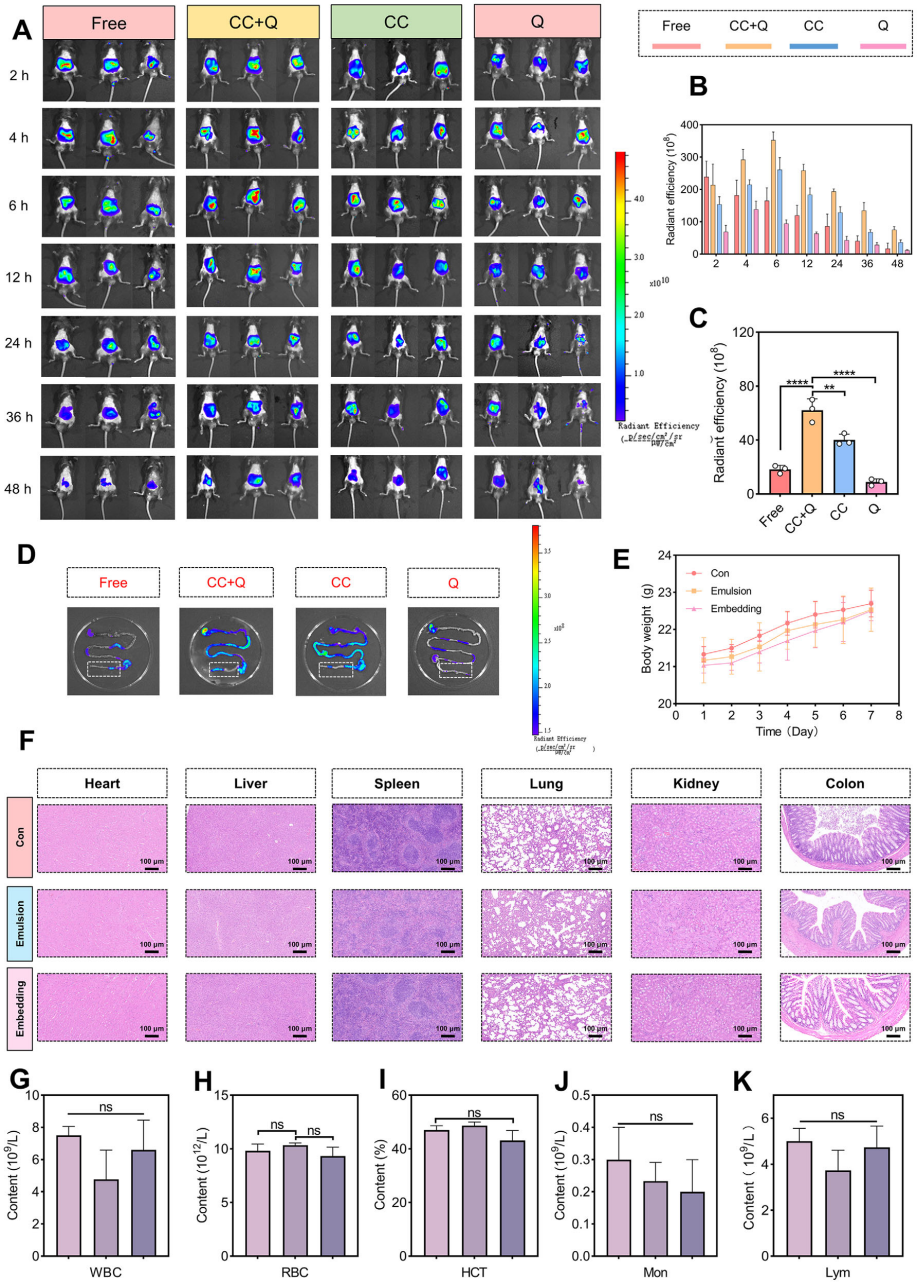
Safety of Catechin/Quercetin@W_1_/O/W_2_ LCC emulsion and fluorescence images of mice in various groups. (A) Fluorescence images were collected at 7 time points (2, 4, 6, 12, 24, 36, 48 h). (B) Quantitative analysis in vivo fluorescence intensity of various groups at different time points. (C) Intensity analysis of fluorescence for mouse organs in each group after 48 h of oral administration. (D) Fluorescence images of the whole gastrointestinal tract after 48 h of oral administration. (E) Changes in mouse body weight. (F) Various organs of different groups of mice (heart, liver, spleen, lungs, kidney, and colon H&E staining), scale bar = 100 µm. (G–K) Blood routine indexes of mice in different groups. *p < 0.05, **p < 0.01, ***p < 0.001, ****p < 0.0001, n = 3.

Analysis of Catechin and Quercetin in mouse feces revealed that release from the W_1_/O/W_2_ emulsion was delayed compared with the free group (Figures S29–S31), aligning with the release of Catechin and Quercetin during digestion (Figure 3D,E). Additionally, to further investigate biodistribution, a delivery system labeled with CY–5.5 was employed for in vivo fluorescence imaging and organ analysis (Figure S32). As shown in Figure 5A,B, compared to the embedding group, the fluorescence signal of the free group significantly decreased in mice at 48 h, the release rules of the Catechin and Quercetin group were consistent with the structural feature of W_1_/O/W_2_ emulsion, which Quercetin was released first in the oil phase, Catechin was then released in inner aqueous phase. Moreover, the endpoint fluorescence of other organs was weak (Figure 5C,D, Figures S33), further confirming the system's ability to enhance colon–targeted delivery efficiency.

Further, we used H&E staining to evaluate the histology morphology of organs, the results revealed that the weight change, organs and blood indicators of mice in different groups did not significantly differ (Figure 5E–K, Figures S34 and S35). Collectively, these findings demonstrated that the Catechin/Quercetin@W_1_/O/W_2_ LCC emulsion exhibits excellent biosafety.

### 2.6 Therapeutic Efficacy of Catechin/Quercetin@W_1_/O/W_2_ LCC Emulsion in DSS–Induced Colitis

**FIGURE 6:**
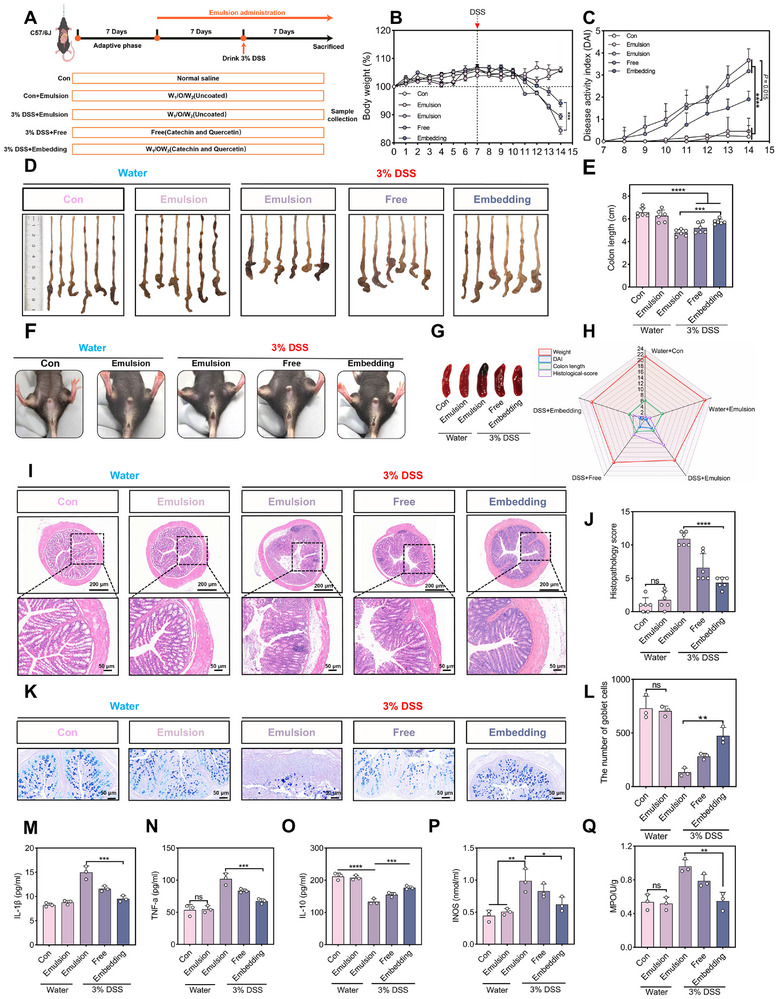
Evaluate the effect of Catechin/Quercetin@W_1_/O/W_2_ LCC emulsion on colitis mice. (A) Experimental design. (B) Weight changes of mice in different groups (n = 6). (C) Change trend of disease pathology score (DAI) in different groups of mice. (D–E) Macroscopic morphology of colon tissues in experimental mice. (F) Hematochezia of mice in different groups. (G)Spleen of mice in different groups (n = 6). (H) Radar chart analysis of macroscopic health indicators in experimental mice. (I–J) H&E staining and scoring of mice in different groups, scale bar = 200 µm, 50 µm. (K–L) AB–PAS staining and goblet cells of mice in different groups, scale bar = 50 µm, n = 3. (M–Q) Inflammatory cytokinesin different groups of mice, n = 3. *p < 0.05, **p < 0.01, ***p < 0.001, ****p < 0.0001.

The therapeutic impact of Catechin/Quercetin@W_1_/O/W_2_ LCC emulsion against colitis was evaluated (Figure 6A). Compared with the Con group, the body weight of mice in the DSS group was reduced by 20.38%, whereas the embedding group significantly mitigated weight loss, with a decrease of only 11.32% relative to the Con group (Figure 6B). Colon assessment revealed edema and shortening to 4.78 ± 0.28 cm in the DSS group, which was markedly alleviated in the embedding group (Figure 6D,E). Furthermore, it was found that a significant increase of DAI (Figure 6C) [25], blood in the stool (Figure 6F), decreased stool (Figure S36) and enlargement of the spleen (Figure 6G,H, Figure S37A,B) in the DSS group, further confirming the successful establishment of DSS‐induced colitis model [2, 46, 47, 48].

To elucidate the mechanism by which Catechin/Quercetin@W_1_/O/W_2_ LCC emulsion alleviates colitis, H&E staining was performed on colon tissues, the results showed that both the Con group and the emulsion group colons had intact colonic mucosa, which was essential for maintaining intestinal health [49, 50, 51] (Figure 6I,J, Figure S38). In contrast, a large reduction in goblet cells and significant deformation of the crypts in the DSS group were observed [52, 53], which were suppressed in the embedding group. For the DSS group, colon tissue scores were reduced by 39.69% and 60.31% in the free and the embedding groups. In Figure 6K,L and Figure S39, the goblet cells and mucins in the DSS group almost disappeared, which were consistent with previous reports [54, 55].

Notably, compared with the DSS group, the embedding group exhibited significantly lower levels of pro–inflammatory cytokines (IL–Iβ, Figure 6M, TNF–α, Figure 6N, Figure S40B,C) was observed, while IL–10 was markedly upregulated, approaching levels observed in the Con group (Figure 6O, Figure S40A). Concurrently, there was a significant reduction in inducible nitric oxide synthase (INOS, Figure 6P) and myeloperoxidase (MPO, Figure 6Q) expression in the embedding group, further demonstrating that W_1_/O/W_2_ emulsion could effectively deliver Catechin and Quercetin to the colon, thereby exerting anti‐inflammatory and antioxidant effects.

In summary, a comprehensive evaluation of body weight, colon length, and pro–inflammatory cytokine levels demonstrated that Catechin/Quercetin@W_1_/O/W_2_ LCC emulsion system significantly enhanced both colonic bioavailability and therapeutic efficacy of Catechin and Quercetin.

### 2.7 Effect of Catechin/Quercetin@W_1_/O/W_2_ LCC Emulsion on the Intestinal Mechanical Barrier of Mice

**FIGURE 7:**
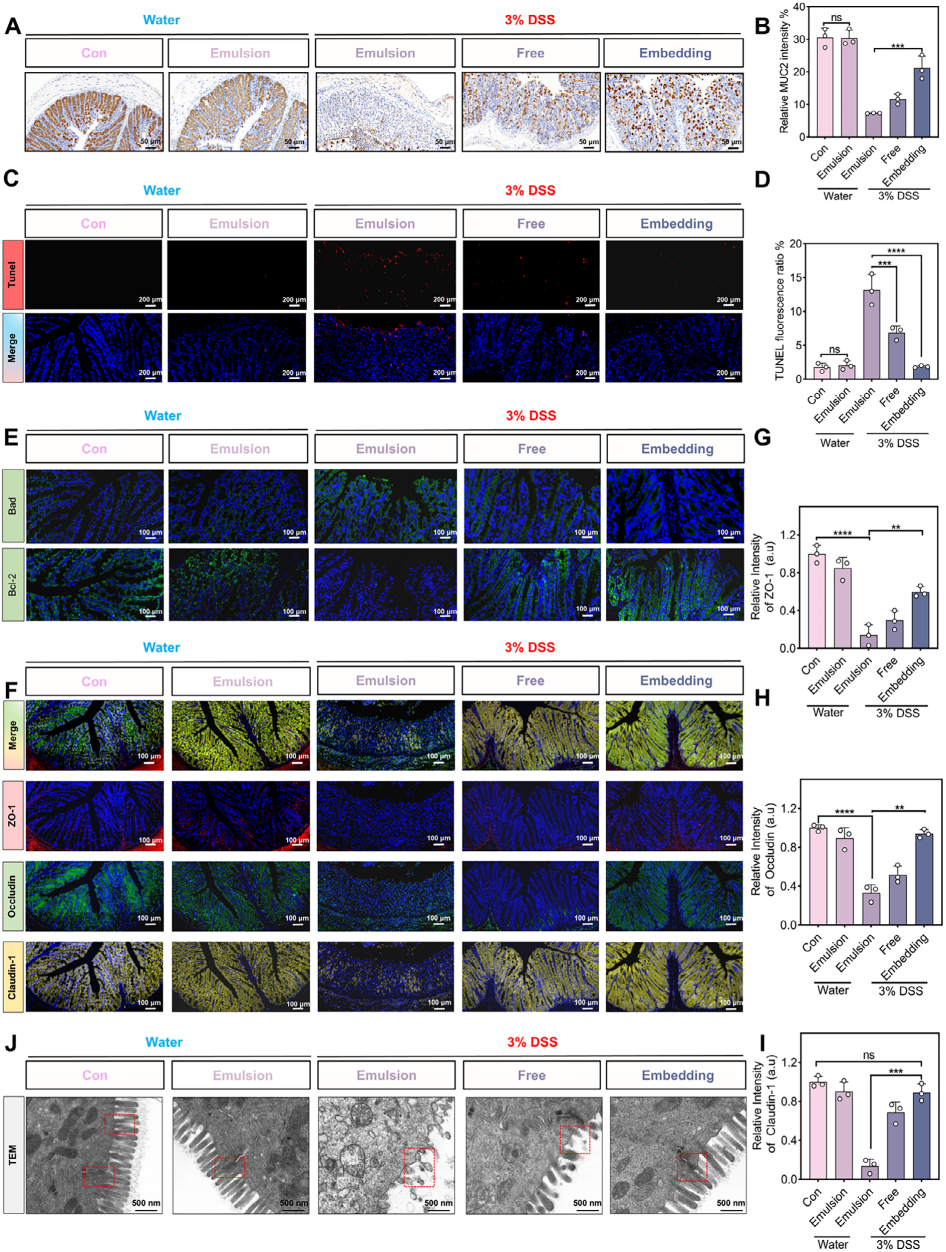
Evaluate the effect of Catechin/Quercetin@W_1_/O/W_2_ LCC emulsion on the intestinal barrier of colitis mice. (A–B) Immunohistochemical analysis of MUC–2 protein in different groups of mice, scale bar = 50 µm. (C–D) Tunnel staining of the colon tissues in different groups, scale bar = 200 µm. (E) Bad, Bcl–2 in colon tissues of mice in different groups, scale bar = 100 µm. (F–I) Tight junction proteins (ZO–1, Occludin, Claudin–1) in different groups of mice, scale bar = 100 µm (n = 3). (J) Microscopic high–power TEM observation of colon tissue in different groups of mice, scale bar = 500 nm (n = 3). *p < 0.05, **p < 0.01, ***p < 0.001, ****p < 0.0001.

The intestinal mucus layer serves as a critical barrier against gut microbiota and pathogenic antigens, which consists of two layers including a strong inner layer (composed of polymerized MUC–2) and a loose outer layer (formed by MUC–2 proteolysis) [26, 53]. In Figure 7A,B and Figure S41, compared with the DSS group, the free and embedding groups exhibited 1.61 times and 2.91 times elevations of MUC–2.

Nevertheless, MUC–2 deficiency impairs mucus barrier integrity, rendering intestinal epithelia prone to damage [56]. Therefore, the apoptosis of colon epithelial cells was further determined by the Tunnel. Specifically, the DSS group exhibited significantly increased apoptosis, whereas the embedding group effectively suppressed apoptotic responses, maintaining levels comparable to the Con group (Figure 7C,D). Consistently, the expression of Bad protein was significantly increased, the expression of Bcl–2 was down–regulated by DSS treatment (Figure 7E, Figure S42), while these alterations were largely reversed in the embedding group, corroborating the Tunnel assay results.

To unravel the pathways of colitis relief in the embedding group, we further investigated changes in the intestinal barrier. Based on the immunofluorescence results (Figure 7G–I, Figure S43), we found that the expressions of tight junction proteins (Occludin, ZO–1 and Claudin–1) were significantly reduced in the DSS group compared to the Con group [53]. In contrast, these proteins were substantially upregulated in the embedding group, indicating that modulation of tight junction protein expression may be a key mechanism for barrier restoration and colitis improvement [57, 58]. Moreover, high–magnification TEM imaging of colon tissues provided ultrastructural evidence supporting the superior therapeutic efficacy of the embedding group. Specifically, the DSS group experienced tight junctions of the colonic epithelium, basic destruction of desmosomes, large fragmentation of microvilli, and swelling of mitochondria, which were consistent with the results of the previous study [59]. As shown in Figure 7J and Figure S44, this group more effectively reconstructed microvilli and tight junctions than the free group. These ultrastructural improvements strongly suggest that our emulsion system ameliorates colitis by repairing the intestinal epithelial barrier, possibly through upregulation of tight junction proteins expression.

### 2.8 Effect of Catechin/Quercetin@W_1_/O/W_2_ LCC Emulsion on Intestinal Microbiota in Colitis Mice

**FIGURE 8:**
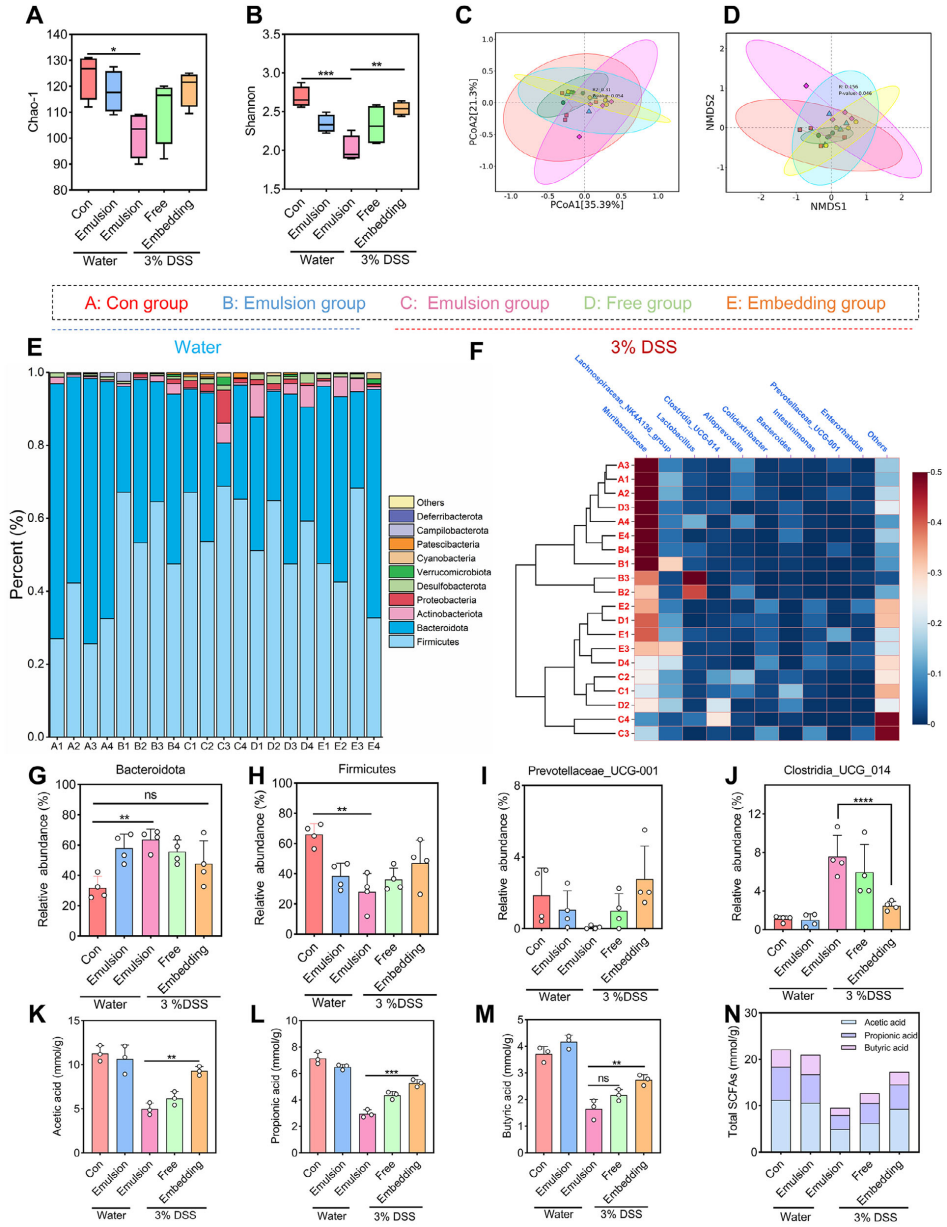
Effects of Catechin/Quercetin@W_1_/O/W_2_ LCC emulsion on gut microbiota (16S rRNA sequencing analysis). (A–B) ASV‐based α index: Chao–1, Shannon. (C–D) ASV–based β index: PcoA and NMDS1 analysis. (E) Relative abundance of microbiota at the phylum level in different groups of mice. (F) Relative abundance of genus–level microbiota in different groups of mice (heat map). (G–J) Relative abundance of microbiota at the phylum level in different groups of mice. Relative abundance of *Bacteroidota*, *Fimicutes*, *Prevotellaceae_UCG–001*, and *Clostridia_UCG_014* in different groups of mice (n = 4). (K–N) Content of Short chain fatty acid (Acetic acid, Butyric acid, Propionic acid) in different groups (n = 3). *p < 0.05, **p < 0.01, ***p < 0.001, ****p < 0.0001.

To investigate the impact of the embedding group on the intestinal microbiota of mice. For the DSS group, the α indices, such as the Shannon and Chao–1 index, were increased after the embedding group treatment (Figure 8A,B, Figure S45). At the ASV classification level, the distribution area of the embedding group and the Con group was closest [57, 60], indicating a restoration of microbial community structure (Figure 8C,D).

**FIGURE 9:**
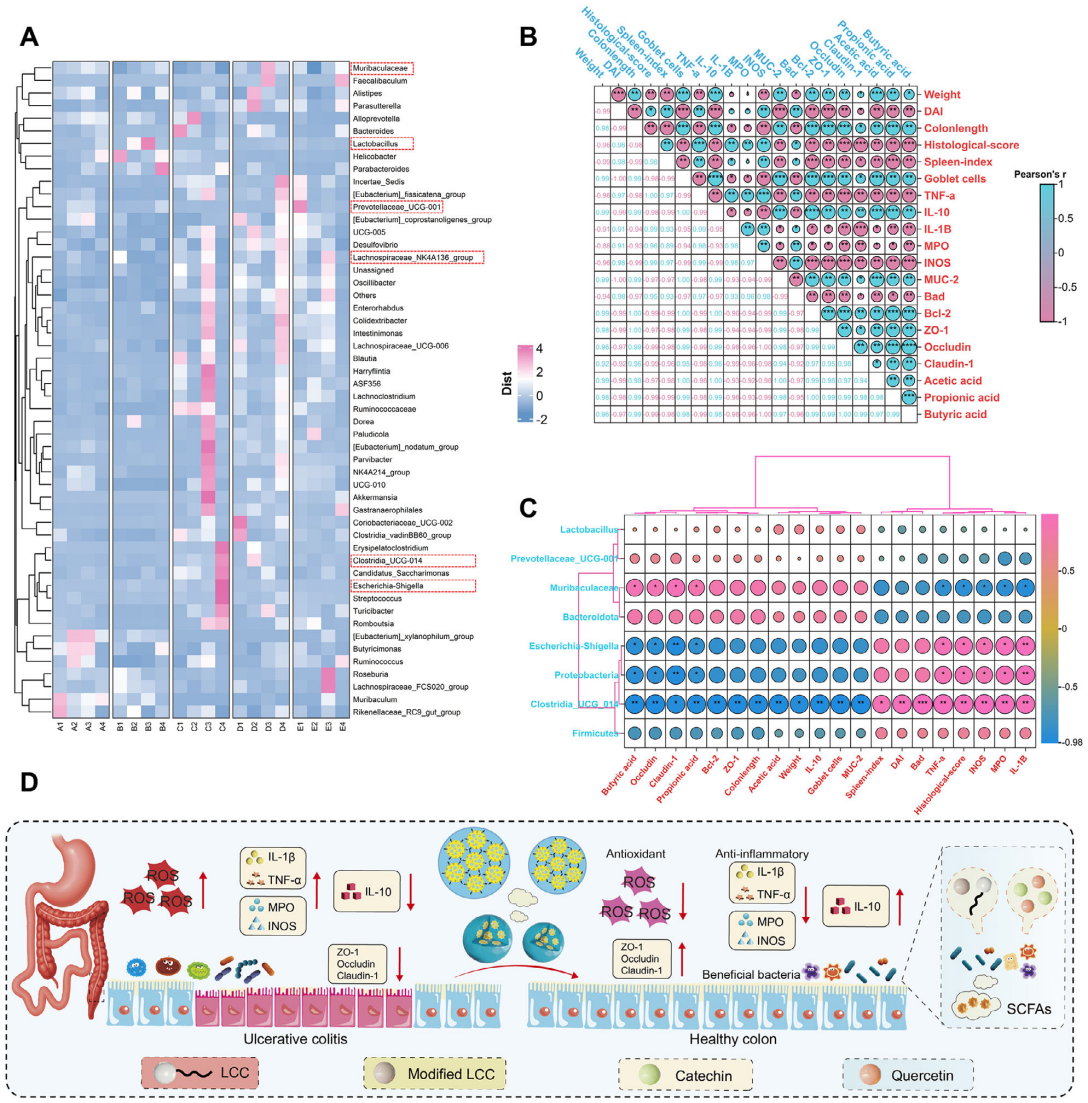
Correlation analysis of significant bacteria with physiological and biochemical indicators of colitis. (A) Relative abundance of species–level microbiota in different groups of mice (heat map). (B–C) Correlation analysis of significant bacteria with Colitis severity (Weight, DAI, Colonlength, Histological–score, Spleen–index, Goblet cells, MPO, INOS, Bad, Bcl–2), Cytokines (TNF–α, IL–10, IL–1β), Intestinal barrier (MUC–2, ZO–1, Claudin–1, Occludin) and Short chain fatty acids (Acetic acid, Propionic acid, Butyric acid). (D) Catechin/Quercetin@W_1_/O/W_2_ LCC emulsion regulates the intestinal flora and its repair in the intestine of colitis mice.

Microbial composition differences among groups were analyzed at both the phylum and genus levels. As shown in Figure 8E,F, *Firmicutes* and *Bacteroidetes* were dominant in each group at the phylum level [4, 60, 61]. Compared with the Con group, the relative abundance of *Firmicutes* in the emulsion group increased. However, the relative abundance of *Proteobacteria* increased and *Bacteroides* decreased in the DSS group (Figure 8G,H). At the genus level, the embedding group exhibited reduced relative abundances of Escherichia–Shigella and Clostridia_UCG_014 compared with the DSS group, both of which are recognized as pathogenic bacteria associated with intestinal inflammation (Figures 8I and 9A, Figures S46 and S47A,B). More importantly, the relative abundance of *Lactobacillus_murinus* in the embedding group increased compared with the DSS group (Figure 8J, Figures S47C–S49). Collectively, these results indicate that the Catechin/Quercetin@W_1_/O/W_2_ LCC emulsion effectively delivers Catechin and Quercetin to the colon, with LCC functioning as a prebiotic to synergistically inhibit pathogenic bacteria while promoting the proliferation of probiotics.

In addition, the content of acetic acid and butyric acid in the feces of mice in the emulsion group increased, which may be due to the degradation and utilization of carbohydrates in LCC by intestinal flora, resulting in the production of acetic acid and butyric acid (Figure 8K–N). Generally speaking, aldehydes (glucose, galactose) are more likely to produce acetic acid and butyric acid [4, 60, 61, 62, 63, 64]. Compared with the DSS group, the embedding group exhibited a significant elevation in SCFA levels, indicating that the W_1_/O/W_2_ emulsion efficiently delivered Catechin and Quercetin while synergistically interacting with LCC to modulate gut microbial metabolism.

Notably, the expressions of ZO–1, Occludin, Claudin–1, and MUC–2 were positively correlated with SCFA (Figure 9B,C). Moreover, *Muribaculaceae* exhibited negative correlations with pro–inflammatory markers TNF–α, IL–1β, and iNOS, while showing positive correlations with IL–10, ZO–1, Occludin, Claudin–1, and MUC–2, which was consistent with the Luo [6]. The Catechin/Quercetin@W_1_/O/W_2_ LCC emulsion significantly reduced the relative abundance of *Clostria_UCG_014*, which was negatively correlated with ZO–1, Occludin, and Claudin–1, and also enhanced the relative abundance of potential beneficial bacteria (*Prevotellacese_UCG_001*), further indicating that restoration of the intestinal barrier via regulation of gut microbiota to resist DSS colitis may be a pathway of the Catechin/Quercetin@W_1_/O/W_2_ LCC emulsion (Figure 9D).

In the DSS group, pathways related to glycan biosynthesis and metabolism, as well as lipid metabolism, were significantly enriched, indicating disruption of cellular lipid homeostasis and impairment of the host intestinal barrier by the microbiota. In contrast, these pathways were effectively suppressed in the embedding group. Furthermore, KEGG prediction revealed that carbohydrate metabolism replaced glycan biosynthesis and metabolism as the most active metabolic module in the embedding group, suggesting a shift in the microbial carbon‑utilization strategy from host‑derived glycans to dietary carbohydrates following oral administration of the Catechin/Quercetin@W_1_/O/W_2_ LCC emulsion (Figures S50 and S51). In conclusion, these results indicated that the key mechanism by which the embedding group ameliorates metabolic and inflammatory diseases likely involves modulation of carbohydrate metabolism and immune responses.

### 2.9 Therapeutic Effects of Catechin/Quercetin@W_1_/O/W_2_ LCC Emulsion in DSS Mice

**FIGURE 10:**
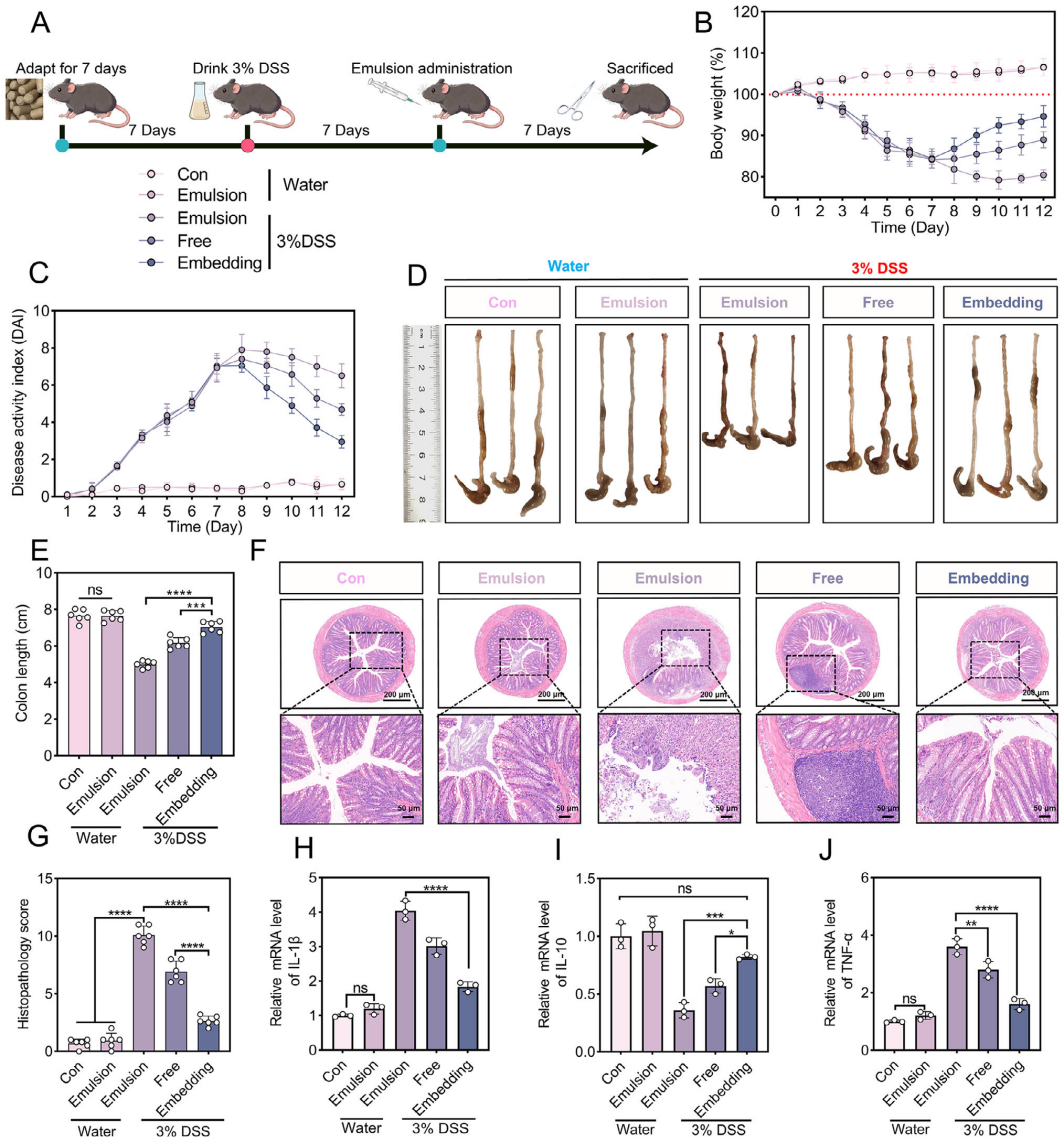
Therapeutic effects of Catechin/Quercetin@W_1_/O/W_2_ LCC emulsion in DSS mice. (A) Experimental design. (B) Weight changes of mice in different groups (n = 6). (C) Change trend of disease pathology score (DAI) in different groups of mice. (D–E) Macroscopic morphology of colon tissues in experimental mice. (F–G) H&E staining and scoring of mice in different groups, scale bar = 200 µm, 50 µm (n = 6). (H–J) Inflammatory cytokinesin different groups of mice. *p < 0.05, **p < 0.01, ***p < 0.001, ****p < 0.0001.

Building on the marked preventive efficacy of the Catechin/Quercetin@W_1_/O/W_2_ LCC emulsion in DSS colitis, we further investigated its therapeutic effect in mice. The experimental design is clearly illustrated in Figure 10A. The body weight loss, colon shortening and splenomegaly (Figure 10B–E, Figure S52) in the DSS group confirmed successful model induction. Furthermore, colonic structure in the embedding group more closely resembled that of the Con group (Figure 10F,G). Notably, oral administration of the Catechin/Quercetin@W_1_/O/W_2_ LCC emulsion reduced the expression levels of TNF–α and IL–1β (Figure 10H,I) while significantly restoring IL–10 expression (Figure 10J). Together, the findings robustly demonstrate that this delivery system possesses not only preventive capacity but also clear therapeutic utility, providing dual evidence for its potential as a treatment for UC.

## 3 Conclusions

Advanced drug delivery systems represent a promising strategy for targeted ulcerative colitis therapy (UC) [1, 4, 5, 15, 46]. In this study, LCC was employed for the first time as emulsifier to formulate a W_1_/O/W_2_ emulsion, demonstrating its effectiveness in the co–encapsulation of Catechin and Quercetin for targeted UC. To mitigate the adverse effects associated with long–term use of chemical emulsifiers [7], we used laccase to catalyze the isoeugenol partial grafting on the lignin in the LCC and yielding a hydrophobic lignin–dominated modified LCC. Notably, the W_1_/O/W_2_ emulsion exhibited optimal physicochemical stability and facilitated controlled intestinal release, consistent with previous observations [65, 66]. The micron‑sized W_1_/O/W_2_ emulsion served as an effective carrier for Catechin and Quercetin, ensuring their oral stability. Intracellular delivery is ultimately achieved through pH‑responsive disassembly during digestion, followed by dynamic self‑assembly of lipid digestion products into polyphenol‑rich micelles leading to a significant enhancement in bioavailability.

Polyphenols have been shown to effectively modulate gut microbiota composition and function [26, 27, 67]. Oral administration of Catechin/Quercetin@W_1_/O/W_2_ LCC emulsion in mice effectively alleviated colitis, including mitigating body weight loss, preventing colon shortening, suppressed the expressions of TNF–α, IL–1β and ameliorated DSS–induced colonic epithelial cell apoptosis. Moreover, the embedding group increased the abundance of *Prevotellaceae_UCG–001, Lactobacillus_mucosicola* produced SCFAs to provide energy for colonic epithelial cells, thereby better restoring the intestinal barrier. Therefore, we hypothesized that the LCC synergistic release of polyphenols alleviated colitis by attenuating the inflammatory response in the colonic epithelium and enriching SCFA–producing bacteria to restore the intestinal mechanical barrier.

However, our study must acknowledge several limitations, advancing toward human application requires systematically addressing comprehensive safety assessments and resolving its generalizability. Future work should extend the platform's capacity to encapsulate more complex active substances, validate its efficacy across a broader range of disease models, and conduct systematic safety assessments. Second, this study only evaluated short–term efficacy in an acute UC model, which limits its direct applicability to chronic disease. Nevertheless, we have introduced a new delivery system Catechin/Quercetin@W_1_/O/W_2_ LCC emulsion. This strategic approach not only offers a concrete, and operationally feasible paradigm for the formulation of pioneering delivery systems that are intrinsically anchored in natural materials, but also unveils an innovative and promising trajectory for the high–value utilization of Lignin–carbohydrate Complex. In doing so, it makes a proactive and substantive contribution to the nation's ambitious and strategically vital objectives of carbon peaking and carbon neutrality.

## 4 Experimental Section

### 4.1 Materials

Pinus massoniana was obtained from Jiangxi Academy of Forestry, China. Dioxane, n–hexane, dimethyl sulfoxide, acetone, acetic acid, ether, and dichloroethane are provided by Sinopharm Chemical Reagents Co.,Ltd. (Shanghai, China). Tetrahydrofuran and isoeugenol are provided by Shanghai Maclean's Biochemical Technology Co., Ltd. (Shanghai, China). Catechin, Quercetin, laccase, and dextran sulfate are provided by Shanghai Yuanye Biotechnology Co., Ltd. (Shanghai, China). Soybean oil is obtained from Arowana Food Group Co., Ltd. CCK–8 reagent, fluorescein isothiocyanate, and reactive oxygen species fluorescent probe were obtained from Biyun Tian Co., Ltd. (Shanghai, China). DMEM medium and fetal bovine serum were obtained from Gibco in the United States. DAPI and paraformaldehyde are purchased from Leagene (Beijing, China).

### 4.2 LCC Extraction and Modification

Wood powder was mixed with benzene and ethanol solution (2:1, v/v), then the powder was milled by vibrating ball milling for 96 h. The sample was added to a dioxane/water mixture (96:4, v/v) and stirred for 24 h, followed by centrifugation (4000 rpm, 10 min) to isolate the insoluble fraction. The mixture was rotary–evaporated at 40°C, transferred to a freeze dryer, and subsequently obtained as a solid sample. Then, the residue was extracted with acetic acid/water (1:1, v/v) for 24 h under identical conditions. After centrifugation (4000 rpm, 10 min), the supernatant was collected. The sample was dissolved in dimethyl sulfoxide (DMSO) and stirred for 12 h, then added dropwise to a dichloroethane/ethanol mixture, followed by centrifugation to isolate precipitate 1. This precipitate was washed sequentially with dichloroethane/ethanol and diethyl ether. Next, precipitate 1 was redissolved in acetic acid/water (1:1, v/v) and stirred for 12 h to obtain solution 1 to add dropwise to acetone, yielding precipitate 2 after centrifugation (4000 rpm, 10 min). Precipitate 2 was washed sequentially with acetone/acetic acid (99:1, v/v), diethyl ether, and petroleum ether, finally vacuum‐dried to obtain the LCC.

LCC was dissolved in a mixture of equal volume buffer solution and anhydrous ethanol (15 mL), then placed in 30°C water bath. Isoeugenol (3 g) was mixed with buffer (35 mL) and laccase (4 mL) was added dropwise using a peristaltic pump (acetate buffer solution (pH = 5.0) was prepared by mixing 6.8 mL of 0.2 M acetic acid with 43.2 mL of 0.2 M sodium acetate solution). The oxygen was introduced continuously, after 5 days, the mixture was centrifuged to collect the insoluble substances (8000 rpm, 10 min) and freeze–dried. The insoluble substances were mixed with a mixture of dichloroethane and ethanol (volume ratio 2:1), and thoroughly washed by centrifugation, then freeze–dried to obtain the modified LCC.

### 4.3 Chemical Compositions of LCC

The chemical compositions of LCC were separated by two–step sulfuric acid hydrolysis. Klason lignin content was determined by the gravimetric method, acid–soluble lignin content was determined by a UV–vis spectrophotometer (Hitachi, Japan), and carbohydrate content was analyzed by HPLC (HP1100, Agilent, USA).

### 4.4 Structural Characterization of LCC

LCC was acetylated with a pyridine–acetic anhydride system to improve its solubility. The acetylated samples were dissolved in tetrahydrofuran, then the molecular weight was determined by gel permeation chromatography (GPC, Wyatt, USA). The mobile phase was pumped at a constant flow rate of 1.0 mL/min, and polyphenylene glycol (PEG) was used as the calibration standard. Fourier transform infrared spectroscopy (FTIR, Nicolet, USA) was used to reveal the functional group changes of the LCC, and the data were collected using a Nexus 470 FTIR spectrometer (wavenumbers: 500 and 4000 cm^−1^ with a resolution of 0.004 cm^−1^ adding 32 scans). X–ray photoelectron spectroscopy (XPS, Physical Electronics, USA) equipped with a monochromatic Alka source (1486.6 eV) was used to analyze the surface chemistry of the LCC. The working voltage was set to 12 kV, and the binding energy scale was calibrated using the C1s peak at 284.8 eV as the reference. The sample was dissolved in DMSO–*d*_6_, then performed with hydrogen–carbon heteronuclear single–quantum correlation (^1^H–^13^C HSQC) NMR. The spectral width was set to 5 MHz in the ^1^H dimension and 18 MHz in the ^13^C dimension. The number of data points in the ^1^H dimension was 1024, and the number of sampling points in the ^13^C dimension was 256.

### 4.5 Organic Solvent Residue Analysis (GC–MS)

Equivalent aliquots of each sample were analyzed using an HP‐5MS column with the following temperature program: 40°C/6 min, ramped to 210°C at 15°C/min and held for 2 min. The split ratio was 50:1 and the flow rate 1 mL/min. Mass spectra were acquired in the range m/z 12–300 using an Agilent 7697A/8860/5977C GC–MS system.

### 4.6 Preparation and Characterization of LCC Emulsions

An aqueous solution of LCC (10 mg/mL) was prepared, and refined soybean oil was used as the oil phase (O). A homogenizer (Ultra Turrax–T18, IKA) was used at 15 000 r/min to prepare O/W emulsions with different water–oil ratios (O: 50%, 60%, 70%, 75%, 80%).

The oil phase (O) was prepared with modified LCC of refined soybean oil (1.5 mg/mL). Deionized water was used as the aqueous phase (W). Homogenization was used at 15 000 rpm to prepare W/O emulsions with different water–oil ratios (W: 20%, 30%, 40%, 50%).

The W_1_/O/W_2_ emulsions were processed by a two–step method: LCC solution (10 mg/mL) was used as the external aqueous phase (W_2_), and W_1_/O colostrum was prepared according to the above method.

### 4.7 Determine the Type of Emulsion

The emulsion was classified as oil–in–water (O/W) if it rapidly dispersed in the aqueous phase but remained agglomerated in the oil phase, otherwise, it was designated water–in–oil (W/O).

### 4.8 Macroscopic Stability of the Emulsions

The emulsions with different oil–water ratios were allowed to stand for 24 h, and photographs were taken to record the degree of oil–water stratification to represent their macro stability.

### 4.9 Emulsion Morphology, Size, and Zeta Potential Determination

The morphology of the emulsion was observed by an Optical microscope (Keens, BZ–X800E, China), and the size analysis of the emulsion was performed by ImageJ–v1.8.0 software. The emulsion was diluted, and the Zeta potential was determined using Nanoparticle size and potential analyzer (Zetasizer, ZS–90, Malvern Instruments Ltd, Malvern, UK).

### 4.10 Rheological Properties of Emulsion

The rheological properties of the emulsion were analyzed using Rotational rheometer (HAAKE, RheoStress 6000, Germany). A strain sweep was performed to determine the linear viscoelastic region, and then the frequency sweep range was set to 0.1–100 Hz, and the storage modulus (G') and loss modulus (G'') as a function of frequency were recorded. The shear rate range was set to 0.01–1000 s^−1^, and the apparent viscosity as a function of the shear rate was recorded.

### 4.11 Two–Chamber, Three–Phase Structure of W_1_/O/W_2_ Emulsion

Laser confocal microscopy (Leica AG, Germany) was used to observe the “two–chamber and three–phase” structure of W_1_/O/W_2_ emulsion. 100 µL Nile red stain solution (0.01% w/v) was added to soybean oil and stirred for 10 min in the dark. The prepared W_1_/O/W_2_ emulsion was then placed on the laser confocal microscope stage. The Nile red fluorescent dye was excited with an argon/krypton laser at 488 nm, and the emitted light was collected through a 525 nm lanmpass filter.

### 4.12 Emulsion Physicochemical Stability

The emulsion was stored at −4°C, 25°C, and 50°C for 30 min, then the Zeta potential was measured to judge its temperature stability. Then, the emulsion underwent 2 freeze–thaw cycles and was followed by Zeta potential monitoring.

### 4.13 Catechin and Quercetin Solubility Assay

Catechin aqueous solution and Quercetin oil solution with different concentrations were prepared. The supernatant was obtained by ultrasound and centrifugation, and diluted 10 times with methanol. Thecontents of Catechin and Quercetin were measured at 280 nm, 370 nm by a UV–vis spectrophotometer (Hitachi, Japan).

### 4.14 Encapsulation Efficiency of Catechin and Quercetin in Emulsion

The emulsion containing Catechin or Quercetin was centrifuged at 4000 r/min to collect the supernatant. Absorbance was measured at 280 nm and 370 nm by a UV spectrophotometer. According to equation (1), where A_0_ denotes the total amount of active substance, A_1_ indicates the amount of active material migration to the external aqueous phase in the newly prepared emulsion.

(1) $$\mathrm{EE}\;\left(\%\right)=\frac{A_{0}-A_{1}}{A_{0}}\times 100\%\;$$

### 4.15 The Microscopic Changes and Zeta Potential Changes of Emulsions During Digestion

The microscopic changes and Zeta potential of emulsions with optimal water ratio were detected at different stages before digestion, gastric, and intestinal stages.

### 4.16 Retention Rates of Catechin and Quercetin During Digestion

The digested samples were mixed with 2.5 mL of ethanol–n–hexane (2:3, V/V), and the n–hexane phase was collected. This was repeated three times, and the extracts were combined. After filtration through a 0.22 µm membrane, the contents of Catechin and Quercetin were analyzed by HPLC–MS (Thermo Fisher Scientific, USA), and the retention rates were calculated according to the equation (2). Where C_0_ is the concentration of Catechin/Quercetin in the emulsion after different digestion stages (mg/mL), C is the initial concentration of Catechin/Quercetin in the emulsion.

(2) $$\mathrm{Retention}\;\mathrm{rate}\;\left(\%\right)=\frac{C_{0}}{C}\times 100\%$$

### 4.17 Antioxidant Activity of Emulsions During Digestion

Sample set: Emulsion digest (100 µL) mixed with 900 µL ABTS^+^ solution and diluted by an appropriate factor. Con group: methanol (100 µL) mixed with ABTS^+^ (900 µL) solution. The absorbance value was determined by a microplate reader at 517 nm by dark treatment for 5 min at room temperature, and the ABTS^+^ clearance was calculated according to the equation (3), where A_0_ is the absorbance of ABTS^+^ at 734 nm, and A_1_ is the absorbance of the sample at 734 nm.

DPPH Radical Scavenging: Sample set: Blank group: Deionized water (0.1 mL) mixed with 1.9 mL DPPH–ethanol solution (0.1 mM). Con group: the digest of different stages of emulsions (0.1 mL) mixed with the ethanol solution (1.9 mL). The absorbance of the sample was measured at 517 nm after standing in the dark for 30 min at room temperature, and the DPPH radical scavenging rate was calculated according to equation (4), where: A_0_ is the absorbance of DPPH at 517 nm, A is the absorbance of the sample mixed with DPPH at 517 nm, and A_b_ is the absorbance of the sample at 517 nm.

(3) $$\mathrm{ABT}S^{+}=1-\frac{A_{1}}{A_{0}}\times 100\%$$

(4) $$\mathrm{DPPH}=\left(1-A-A_{b}\times A_{0}\right)\times 100\%$$

### 4.18 Bioavailability Rates of Catechin and Quercetin During Digestion

The intestinal digest emulsions were centrifuged (10 000 rpm, 4°C, 30 min), which the micellar layer was filtered (0.45 µm membrane) and analyzed for Catechin and Quercetin contents via HPLC–MS. The bioaccessibility rate (BA) was calculated by Equation (5), where: C_1_ is the mass concentration of Catechin/Quercetin in the micelle part (mg/mL), C_0_ is the initial concentration of Catechin/Quercetin in the emulsion.

(5) $$\mathrm{BA}\;\left(\%\right)=\frac{C_{1}}{C_{0}}\times 100\%$$

### 4.19 Release Rates of Catechin and Quercetin During Digestion

At 40 min intervals, aliquots were withdrawn, mixed with three times of volume of anhydrous methanol, and vortexed (the digest volume maintained by equal replenishment). After centrifugation (8000 rpm, 10 min), supernatants were subjected to LC–MS for Catechin/Quercetin quantification. The contents were calculated according to equation (6), where C_0_ represents the Catechin or Quercetin content in the supernatant and C represents the initial Catechin or Quercetin concentration

(6) $$\mathrm{Release}\;\mathrm{ratio}=\frac{C_{0}}{C}\times 100\%$$

### 4.20 Caco–2 Cell Culture

DMEM complete medium (pH = 7.4) containing 10% fetal bovine serum and 1% bispecific antibody was incubated at 37°C. The cells were washed three times with PBS and then treated with 2 mL of 0.25% trypsin–EDTA at 37°C for 2 min. After centrifugation at 1000 rpm for 5 min, the cells were passaged at a 1:2 ratio into a new flask.

### 4.21 Effect of Catechin/Quercetin@W_1_/O/W_2_ LCC Emulsion Micelle on Caco–2 Cell Viability

The cell counting kit (CCK–8) was used to detect the effect of Catechin/Quercetin@W_1_/O/W_2_ LCC emulsion on cell viability. Catechin/Quercetin@W_1_/O/W_2_ LCC emulsions were co–cultured with cells at different final concentrations, corresponding to catechin levels of 1.05 µg/mL (400 times), 1.2 µg/mL (350 times), 1.4 µg/mL (300 times), and 1.68 µg/mL (250 times), and quercetin levels of 0.0375 µg/mL (400 times), 0.0429 µg/mL (350 times), 0.05 µg/mL (300 times), and 0.06 µg/mL (250 times). Test samples (100 µL) were dispensed into wells in euplicate, while negative controls received culture medium alone (100 µL). CCK–8 reagent was added to each well and incubated at 37°C for 2 h. The microplate reader reads the absorbance value at 450 nm. Cell viability is calculated according to equation 7, where: sample A_1_ is the absorbance value of the sample group, control A is the absorbance value of the complete medium after replacing the sample, and A_0_ is the absorbance value of the blank group.

(7) $$Cell\;viability=\frac{A_{1}-A_{0}}{A-A_{0}}\times 100\%$$

### 4.22 Effect of Catechin/Quercetin@W_1_/O/W_2_ LCC Emulsion on Oxidative Damage in Caco2 Cells

Caco‐2 cells were treated with 1.2 mM H_2_O_2_ for 2 h to establish the oxidative damage model. Intracellular ROS was quantified using 3 µL DCFH‐DA (15 min, dark), followed by three PBS washes prior to fluorescence measurement. Photographs were acquired under a fluorescence microscope and analyzed using ImageJ–v1.8.0.

### 4.23 Uptake of Catechin/Quercetin@W_1_/O/W_2_ LCC Emulsion by Caco–2 Cells

Referring to the method of Zhou [68], the fluorescently stained W_1_/O/W_2_ emulsion (1 mL) was added to the Petri dish for co–incubation (2 h, 6 h) in the dark. Cells were fixed with 4% paraformaldehyde solution, washed with PBS (1 mL), DAPI (5 µg/mL) to stain the nuclei. Observed under a fluorescence microscope, the acquired photographs were analyzed for fluorescence intensity using ImageJ–v1.8.0. Cells were washed with PBS, digested, and collected into EP tubes. After centrifugation at 4°C (1000 rpm, 3 min), the supernatant was discarded and the cell pellet was retained. The pellet was resuspended in PBS and centrifuged again under the same conditions. The final pellet was filtered and subjected to flow cytometry analysis.

### 4.24 Hemocompatibility of Catechin/Quercetin@W_1_/O/W_2_ LCC Emulsion

The mouse blood was washed with PBS, centrifuged (1000 g, 5 min), and the red blood cell pellet was collected. Erythrocyte suspension (200 µL) was mixed with the W_1_/O/W_2_ emulsion (800 µL) at dilutions of 0, 10 x, 20 x, 50 x, and 100 x to incubate at 37°C for 3 h, then centrifuged (4000 g, 5 min). The absorbance (OD) value was measured at 540 nm using a microplate reader. The Water group was the positive control (100% hemolysis), and the PBS group was the negative control (0% hemolysis). Finally, the hemolysis rate is calculated using Equation (8), where A: OD value of the sample solution, A_0_: OD value of PBS, A_1_: OD value of 100% hemolysis.

(8) $$\mathrm{Hemolysis}\;\mathrm{ratio}=\frac{A-A_{0}}{A_{1}-A_{0}}\times 100\%$$

### 4.25 Biosafety and Blood Routine

The experimental period was 8 days, and male mice (8 weeks) were divided into 3 groups: Con group, emulsion group, and embedding group (n = 3); the mice were sacrificed on the eighth day. Collect individual organs (heart, liver, spleen, lung, kidneys, and colon for H&E staining) and whole blood for routine blood testing.

### 4.26 In Vitro Imaging

The experiment was divided into 4 groups: free, embedding (Catechin + Quercetin), embedding (Catechin) and embedding (Quercetin), and each group was given an equal amount (300 uL) of drug by gavage, the vivo fluorescence imaging and ex vivo fluorescence imaging of mouse organs were performed at (2/4/6/12/24/36/48 h), respectively, with 3 male mice (C57/6J) in each group. CY–5.5 was coupled with Catechin and Quercetin, respectively, to construct markers with excitation wavelengths of 695 nm and emission wavelengths of 720 nm.

### 4.27 DSS Induced Prevention of Colitis Mouse Model

Week C57/6J male mice were raised at an ambient temperature of 25°C, a humidity of 60%, and a cycle of 12 h of light exposure / 12 h of night. First, the mice were adapted for 7 days, then the formal experiment (8–21 days), the mice were randomly divided into 5 groups (n = 6): Five experimental groups were established in mice as follows: Con group: received sterile drinking water. Emulsion group: administered the LCC–stabilized blank emulsion by oral gavage. DSS–Emulsion group: given 3% DSS. Free group: treated with unencapsulated Catechin and Quercetin via oral gavage while receiving 3% DSS. Embedding group: administered the Catechin/Quercetin@W_1_/O/W_2_ LCC emulsion orally alongside 3% DSS, the specific experimental design was shown in Table S7a. On the last day, mice were sacrificed, collected the mouse colons, colon contents. The distal colons were fixed with 4% paraformaldehyde for 0.5 cm, and then used for H&E, immunohistochemistry, and immunofluorescence analysis. Another 0.5 cm of the distal colons were placed in the electron microscope fixative solution, and then used as TEM sample.

### 4.28 DSS Induced Therapeutic Colitis Mouse Model

The mice were adapted for 7 days, then the formal experiment (8–21 days), the mice were randomly divided into 5 groups (n = 6): Five experimental groups were established in mice as follows: Con group: received sterile drinking water. Emulsion group: administered the LCC–stabilized blank emulsion by oral gavage. DSS‐Emulsion group: given 3% DSS. Free group: treated with unencapsulated Catechin and Quercetin via oral gavage while receiving 3% DSS. Embedding group: administered the Catechin/Quercetin@W_1_/O/W_2_ LCC emulsion orally alongside 3% DSS.

### 4.29 Colitis Mouse Body Weight and Disease Score (DAI)

The mice were weighed at the same time each day, and the Disease Activity Index score table is shown in (Table S7b), DAI = (weight change score + stool shape score + hematochezia score) /3.

### 4.30 Spleen Index

The organ index of the spleen was calculated according to the Equation (9), where SW stands for spleen weight and BW stands for body weight.

(9) $$\mathrm{Spleen}\;\mathrm{index}=\frac{\mathrm{SW}}{\mathrm{BW}}\times 100\%$$

### 4.31 Changes in Fecal Catechin and Quercetin Content at 24 Hours

The feces of mice in the free group and the embedding group were collected for 24 h. Fecal samples were homogenized with methanol (1:20, w/v) and sonicated for 1 h. The extracts were centrifuged (12 000 g, 4°C, 15 min), and the supernatant was incubated at 4°C for 1 h before filtration through a 0.22 µm membrane. Catechin and Quercetin were quantified by LC–MS at 280 nm and 370 nm.

### 4.32 Histopathological Evaluation

Colons were fixed in 4% paraformaldehyde and embedded with paraffin before histological staining. H&E sections were scored from three aspects: lesion depth, crypt damage, and lesion extent, and the specific scoring details are shown in Table S7c. To assess mucosal integrity and secretory function, colons were stained with AB–PAS. In addition, the expression of mucin MUC–2 was evaluated by immunofluorescence histochemistry. ImageJ–v1.8.0 was used to evaluate goblet cells and measure the average optical density of MUC–2 protein in AB–PAS sections.

### 4.33 Determination of Inflammatory Factors and Oxidative Stress–related Indicators in the Colons

An appropriate amount of colon tissues was removed, tissue lysate was added 1:9 after thawing, and the colon tissue was broken by a high–throughput disruptor (65 Hz, 25 s) to obtain a homogeneiye, then centrifuged at 12 000 rpm and 4°C for 15 min to collect the supernatant. An ELISA kit was used to determine the levels of inflammatory factors (TNF–α, IL–1β, IL–10) and inducible nitric oxide synthase (INOS) in the colon tissues of colitis mice. Referring to the operation instructions of the kit (Nanjing Jiancheng Institute of Bioengineering), MPO activity was determined by the MPO colorimetric method.

### 4.34 Colon Tunnel Staining

Apoptosis was detected using the terminal deoxynucleotidyl transferase (dUTP) nick end labeling (Tunel) method and were analyzed by ImageJ–v1.8.0 software.

### 4.35 Immunofluorescence Staining

Immunofluorescence was used to observe the expression of apoptosis protein (Bad/Bcl–2) and the expressions of colon tissue tight junction proteins Claudin–1, Occludin, and ZO–1. The mean fluorescence intensities of the positive area under the microscope were determined by ImageJ–v1.8.0 software.

### 4.36 RT–qPCR Analysis

Caco‑2 cells were pretreated with Catechin/Quercetin@W_1_/O/W_2_ LCC emulsion for 24 h, followed by stimulation with LPS (100 ng/mL) for 5 h. Cells were then collected for RT‑qPCR.

Colonic Tissue Analysis: total RNA was extracted from colon samples using VeZol Reagent (Vazyme Biotech Co., Ltd), reverse‑transcribed into cDNA with HiScript II Reverse Transcriptase (Vazyme Biotech Co., Ltd), and subjected to qPCR analysis using ChamQ Universal SYBR qPCR Master Mix (Vazyme Biotech Co., Ltd). The relative mRNA expression levels of TNF‑α, IL‑10, and IL‑1β were normalized to the reference gene. Primer sequences are listed in Table S6a,b.

### 4.37 Short Chain Fatty Acids (SCFAs)

Propanol (0.8 mL) was added to feces (0.8 mL), vortexed fully, and let stand for 5 min. Subsequent centrifugation at 8000 r/min, 5 min at 4°C, supernatant (0.5 mL) was aspirated, filtered through a 0.22 µm nylon filter membrane, and used for GC/MS (Agilent, USA) analysis.

### 4.38 16S Sequencing Detection of Intestinal Flora

Total DNA was extracted from stool samples and tested by agarose gel electrophoresis to confirm the size and integrity of the extracted product. Specific sequencing adapters and Barcode sequences are ligated to both ends of the PCR product, and the PCR amplification product is purified using magnetic beads to remove unnecessary impurities. The libraries were quantified and normalized, and DNB was prepared after mixing. DNB was loaded onto the high–throughput sequencing platform DNBSEQ–G99RS, and PE300 was selected for paired–end sequencing.

### 4.39 Statistical Analysis

All experimental results were expressed as mean ± standard deviation (SD). One–way ANOVA was used to compare the differences between different groups, and post–hoc analysis was used to compare the Turkey multiple comparison test. All data were analyzed by GraphPad Prism (9.5.0), Excel (2016), ImageJ–v1.8.0, and Origin (2024), Pearson correlation analysis was used to analyze the correlation between physiological and biochemical indexes and microbiota in mice with colitis, and the correlation heat map was plotted by ChiPlot (https://www.chiplot.online/). Statistical significance was expressed as *p < 0.05, **p < 0.01, ***p < 0.001, ****p < 0.0001.

## Funding

This work was financially supported by the National Natural Science Foundation of China (No.32472341 and 21908048), Hubei Provincial Natural Science Foundation for Distinguished Young Scholars (No. JCZRJQ202500133), Natural Science Foundation of Hubei Province (No. 2024AFD281), and Science and Technology Research Project of Education Department of Hubei Province (No. F2023006).

## Ethical Statement

This study and included experimental procedures were approved by Hubei University of Technology (approval No. HBUTXM20250043). All animal housing and experiments were conducted in strict accordance with the institution for care and use of laboratory animals.

## Conflicts of Interest

The authors declare no competing financial interest.

## Supporting information




**Supporting File**: advs74760‐sup‐0001‐SuppMat.pdf.

## Data Availability

The data that support the findings of this study are available from the corresponding author upon reasonable request.
