# Supplementary material for: Constraint of Lignin–Carbohydrate Complex Orchestrated on Polyphenol in Oil–Water Interface Targeting Ulcerative Colitis Therapy
Source: Adv Sci (Weinh). 2026 Mar 12;13(29):e24070. doi: 10.1002/advs.202524070 (PMC13205672; doi:10.1002/advs.202524070)
Supplement: Supplementary file 1 — Supporting File: advs74760‐sup‐0001‐SuppMat.pdf. [file ADVS-13-e24070-s001.pdf]

Constraint of Lignin-carbohydrate Complex Orchestrated on Polyphenol in Oil-water Interface Targeting Ulcerative Colitis Therapy

Qian Wu[a], *Xingyu Zhang*[a], *Jingjia Zhang*[a], *Gaohui Huang*[a], *Chen Zhou*[a], *Chunlin Li*[c]*, *Xiaojun Huang*[e], *Jianbo Xiao*[d], *Nianjie Feng*[a]*, and *Yuanbin She*[b]*

a. National "111" Center for Cellular Regulation and Molecular Pharmaceutics, Hubei University of Technology, Wuhan, Hubei 430068, China

b. State Key Laboratory of Green Chemical Synthesis and Conversion, College of Chemcal Engineering， Zhejing Uiversity of Technolgy, Hangzhou, Zhejiang 310014, China

c. State Key Laboratory for Quality and Safety of Agro-Products, Institute of Agro-Products Safety and Nutrition, Zhejiang Academy of Agricultural Sciences, Hangzhou, Zhejiang 310021, China

d. Research Group on Food, Nutritional Biochemistry and Health, Universidad Europea del Atlántico, Isabel Torres 21, Santander 39011, Spain

e. State Key Laboratory of Food Science and Resources, China-Canada Joint Lab of Food Science and Technology (Nanchang), Nanchang University, Nanchang 330047, China

***Corresponding author**: Yuanbin She[b]* (sheyb@zjut.edu.cn), Nianjie Feng[a]* (njfeng@hbut.edu.cn), and Chunlin Li[c]* (chunlinli0304@163.com)

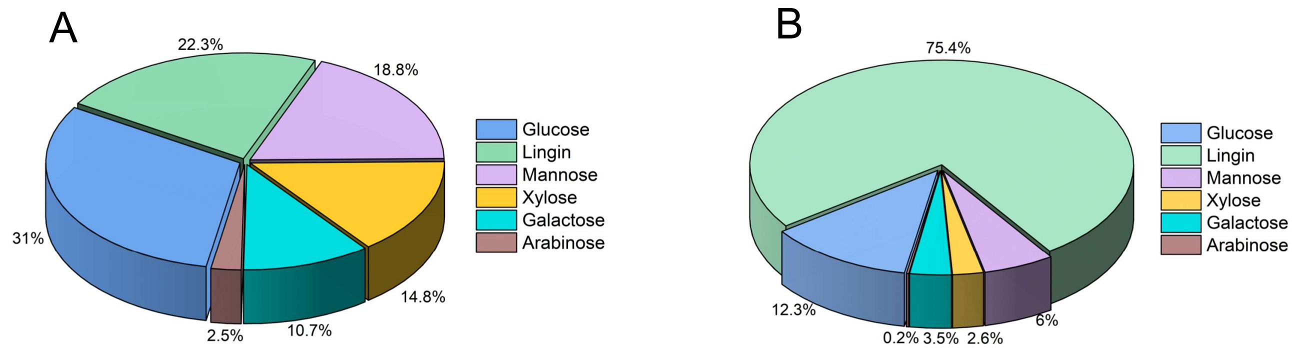

Figure S1. Chemical composition analysis of LCC/modified LCC.

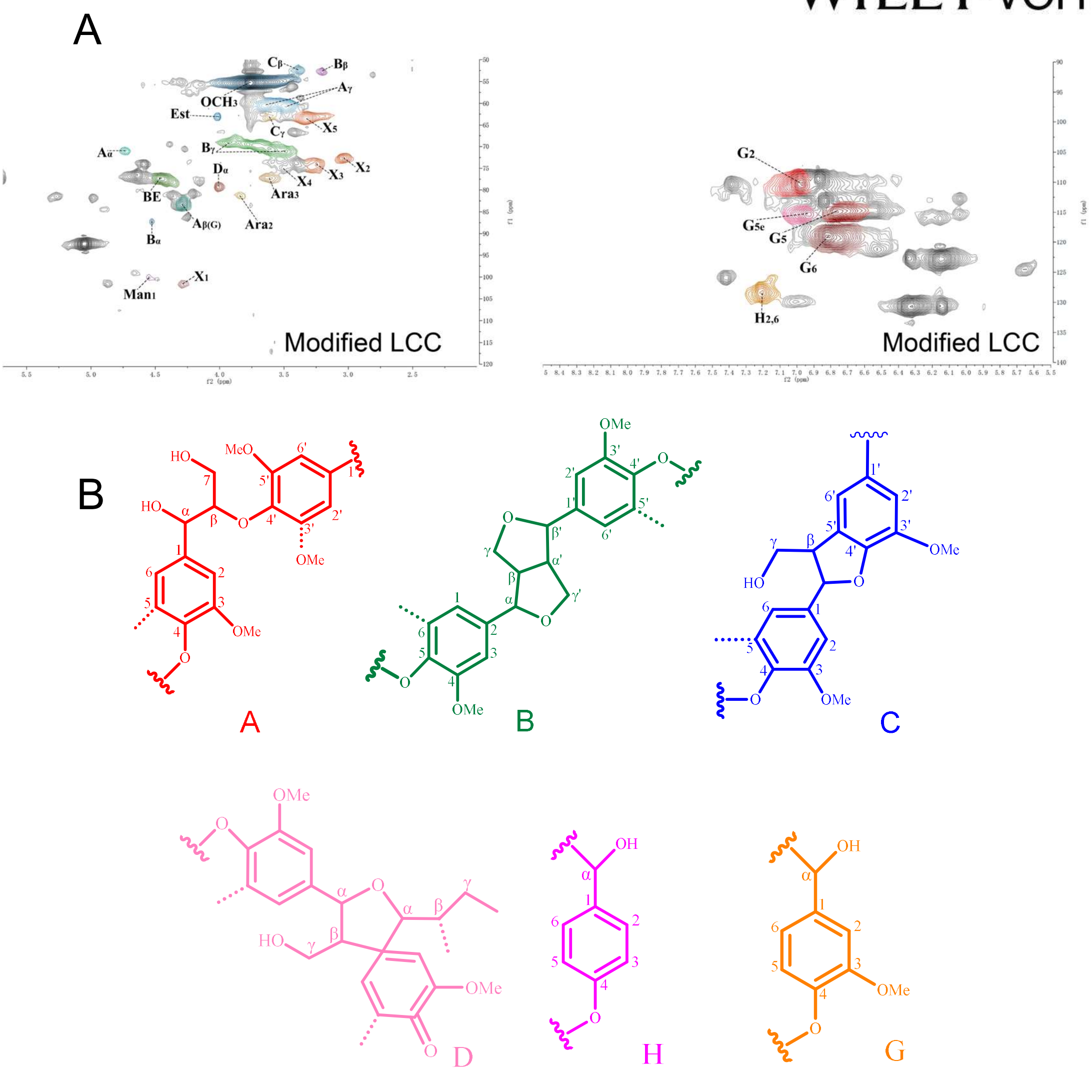

Figure S2. 2D-HSQC NMRspectroscopy of Modified LCC. A) Aromatic regions (δC/δH 140-90/8.5-5.25) and aliphatic regions (δC/δH 120-50/6.0-2.0) in HSQC spectra of Modified LCC: (A) *β*-O-4 ether bond structure. (B) Resin alcohol structure. (C) Phenyl coumarin structure. (D) spirodienones. (G) Yuchuang wood-based unit. (H) Hydroxyphenyl unit. (X) *β*-D-xylose. (Ara) *α*-L-arabinofuranose. (Glc) *β*-D-glucopyranose. (Man) *β*-D-mannose. (Gal) *β*-D-galactoside. (Est) gamma ester bond. (BE) benzyl ether bond. B) The main basic connecting structures and structural units in 2D-HSQC NMR spectroscopy.

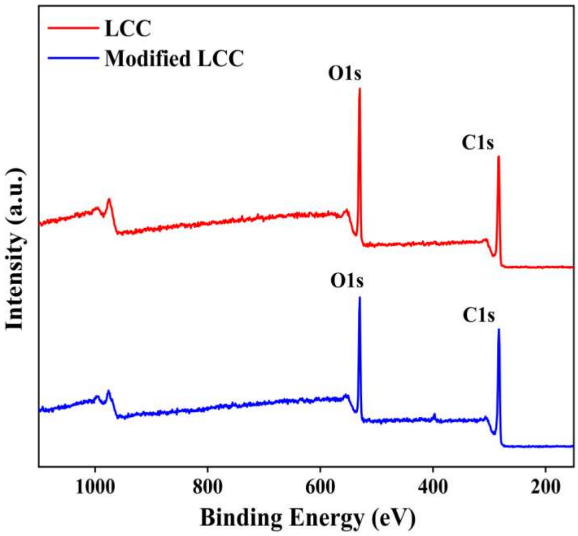

Figure S3. XPS full-spectrum analysis of LCC and modified LCC.

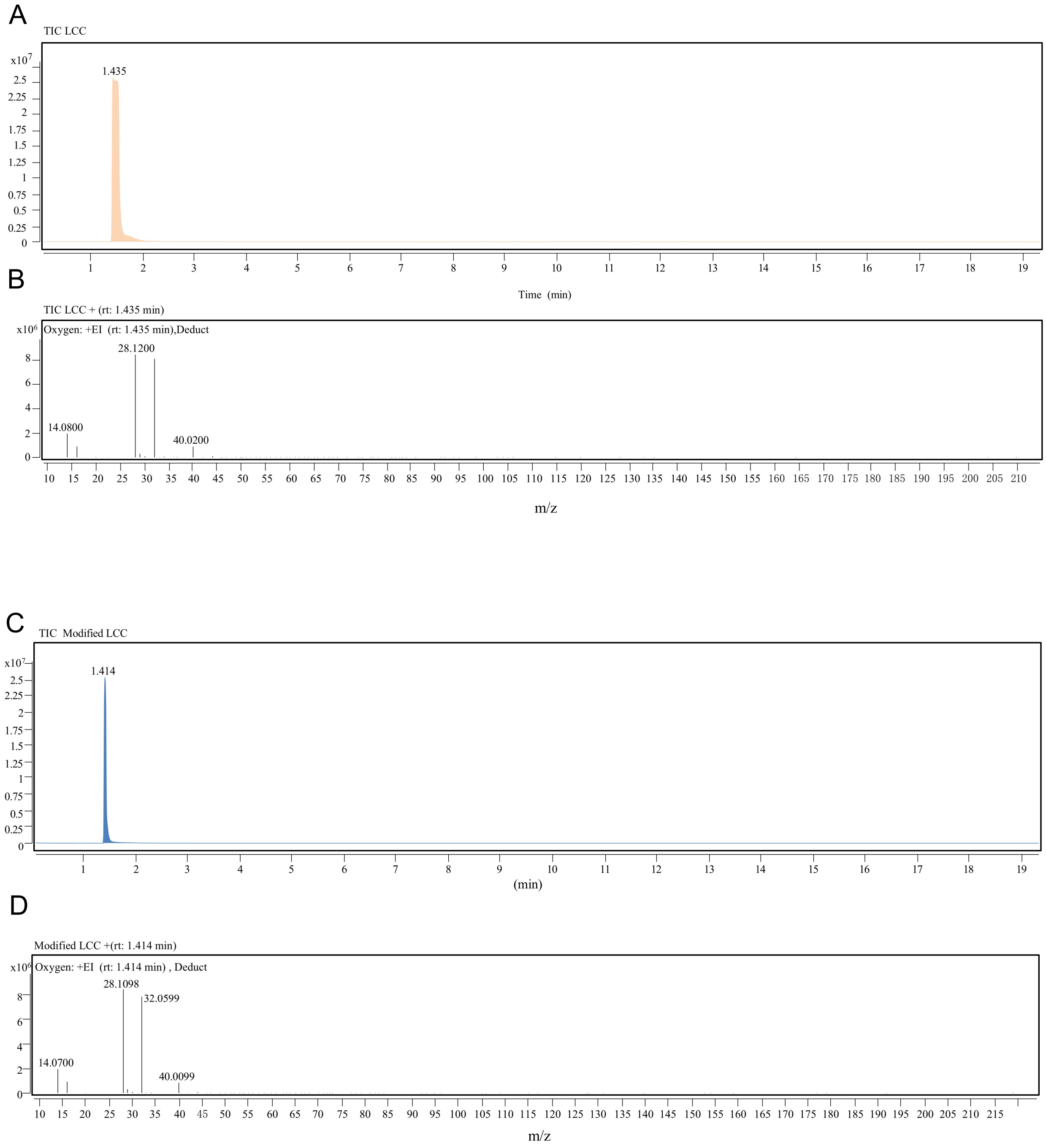

Figure S4. Detection of organic solvent residues in LCC samples by GC-MS. A-B) Chromatograms and mass spectra of organic solvent residue extraction from LCC samples. C-D) Chromatograms and mass spectra of organic solvent residue extraction from modified LCC samples.

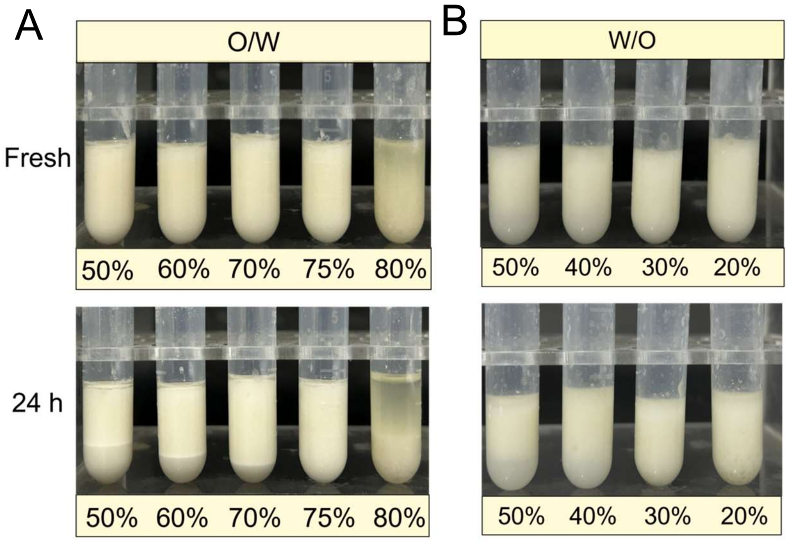

Figure S5. Stability of macroscopic view of the emulsions, the oil-water ratio increases from left to right. A) Macrograph of oil in water emulsions with different oil-water ratios (LCC in aqueous phase). B) Macrograph of water in oil emulsions under different oil-water ratios (oil phase contains modified LCC).

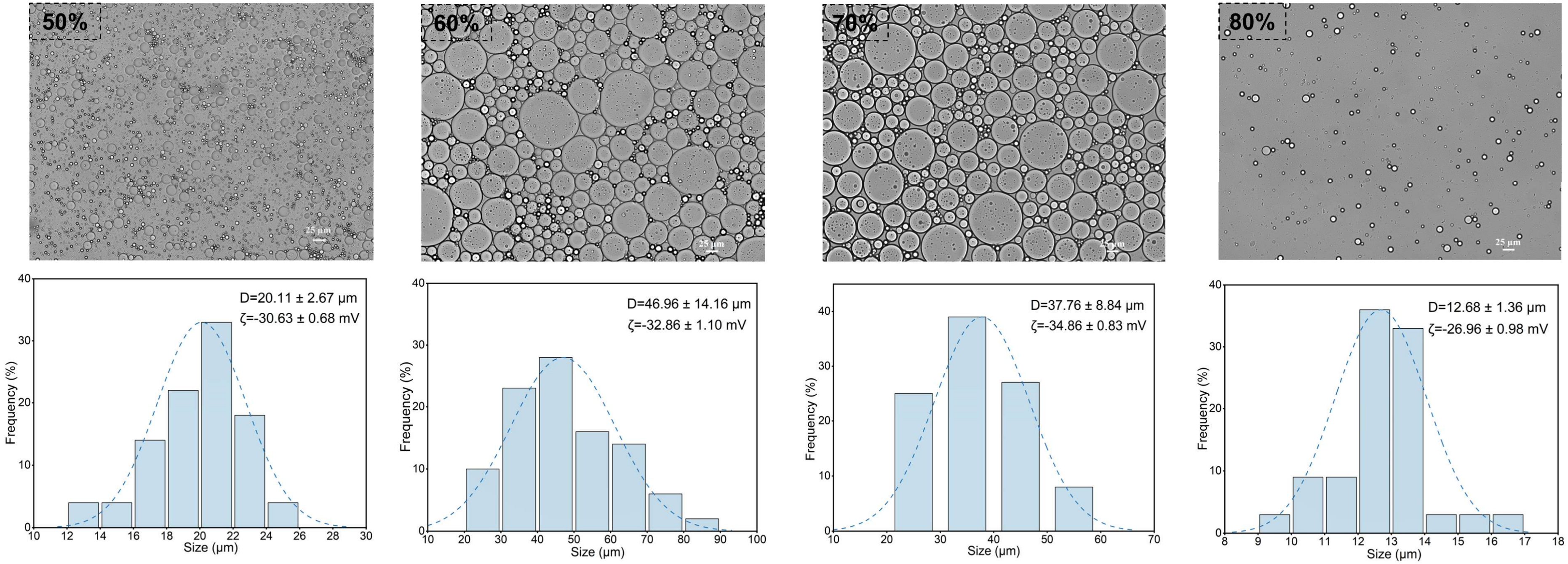

Figure S6. Oil in water emulsion with different oil-water ratios: micrograph, particle size distribution, and Zeta potential, n=3.

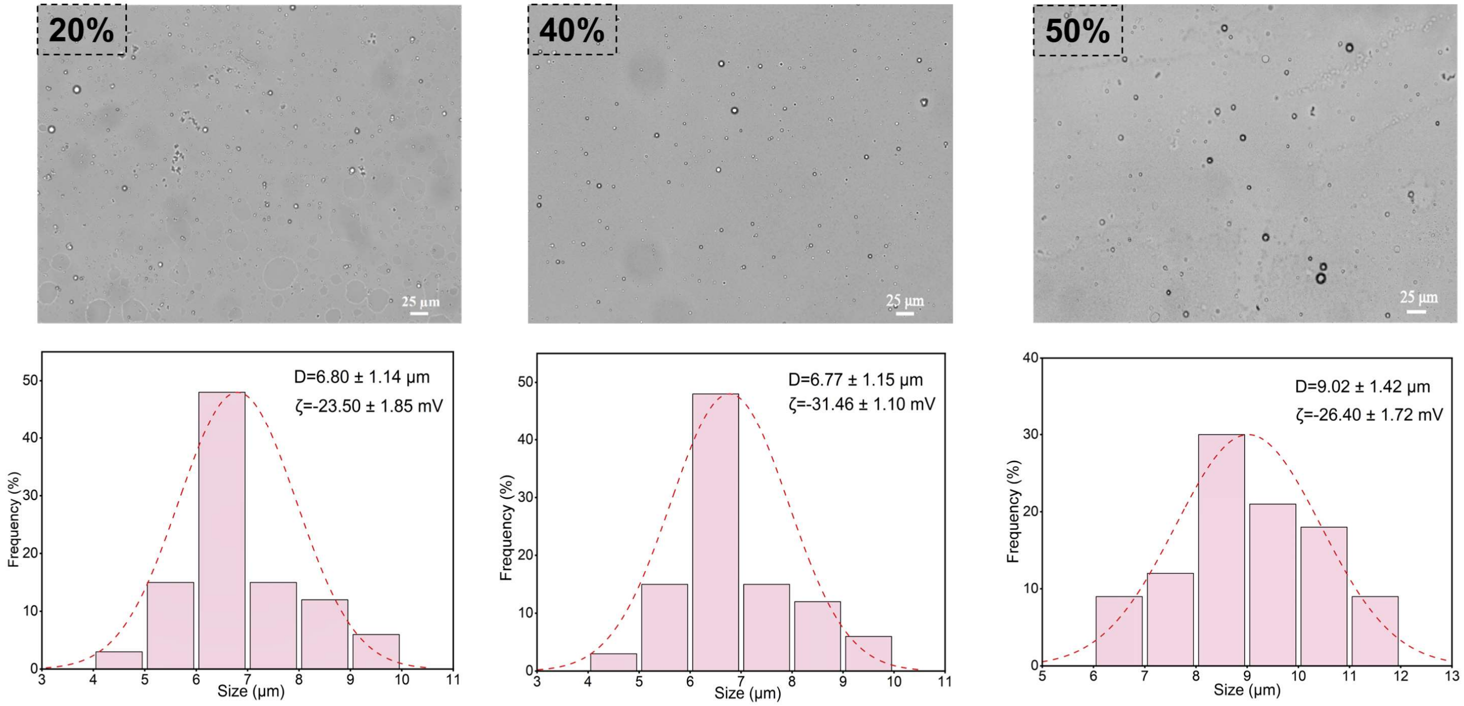

Figure S7. Water in oil emulsion under different oil-water ratios: micrograph, particle size distribution, and Zeta.Potential, n=3.

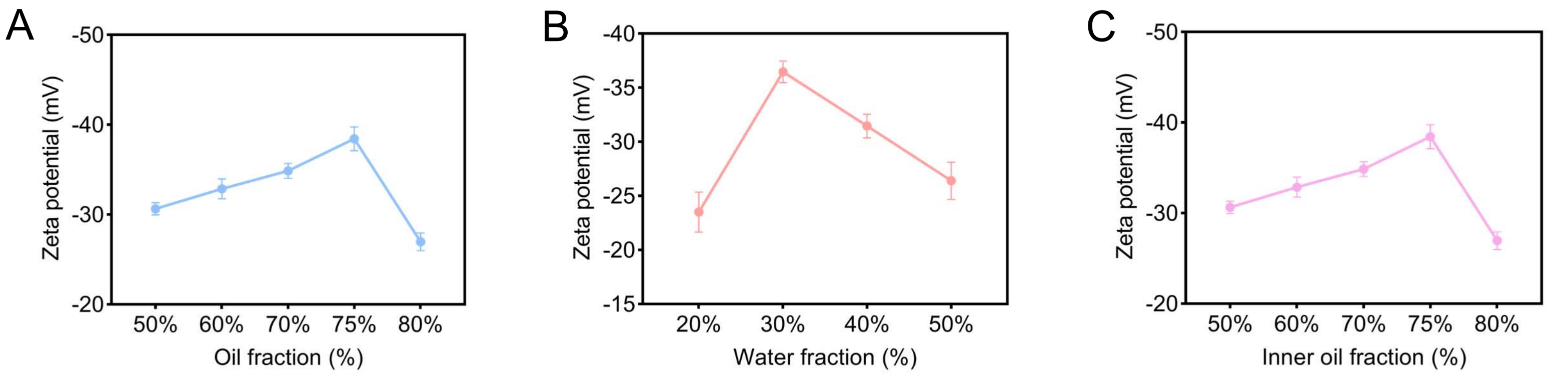

,

Figure S8. Zeta potential of emulsions. A) Zeta potential of O/W emulsion. B) Zeta potential of O/W emulsion. C) Zeta potential of W_1/O/W_2 emulsion, (n=3).

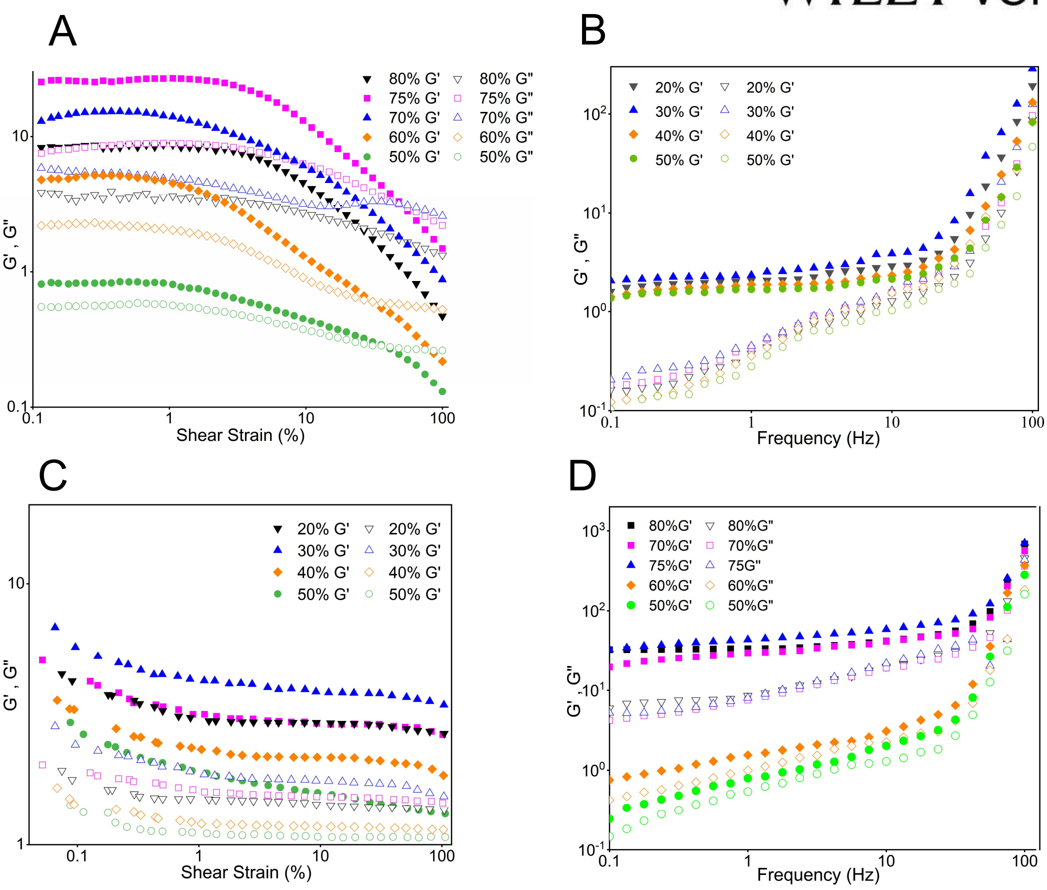

Figure S9. Rheology of emulsions. A) Rheology of oil in water emulsion: stress and strain. B) Rheology of oil in water emulsion: modulus. C) Rheology of water in oil emulsion: stress and strain. D) Rheology of water in oil emulsion: modulus.

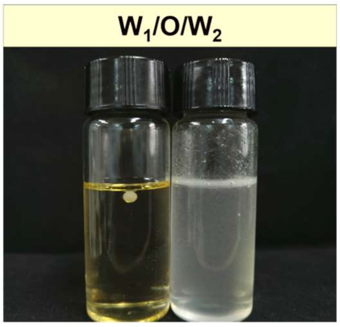

Figure S10. Schematic diagram of emulsions preparation and titration test: judge the type of emulsions: $W_1/O/W_2$ emulsion.

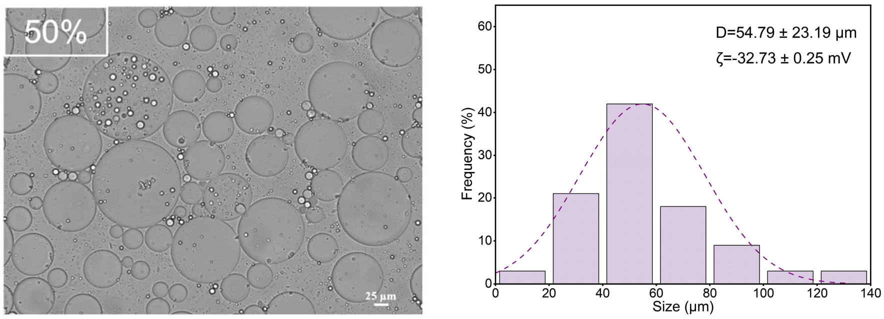

Figure S11. $W_1/O/W_2$ emulsion under different oil-water ratios: micrograph, particle size distribution, and Zeta potential (n=3).

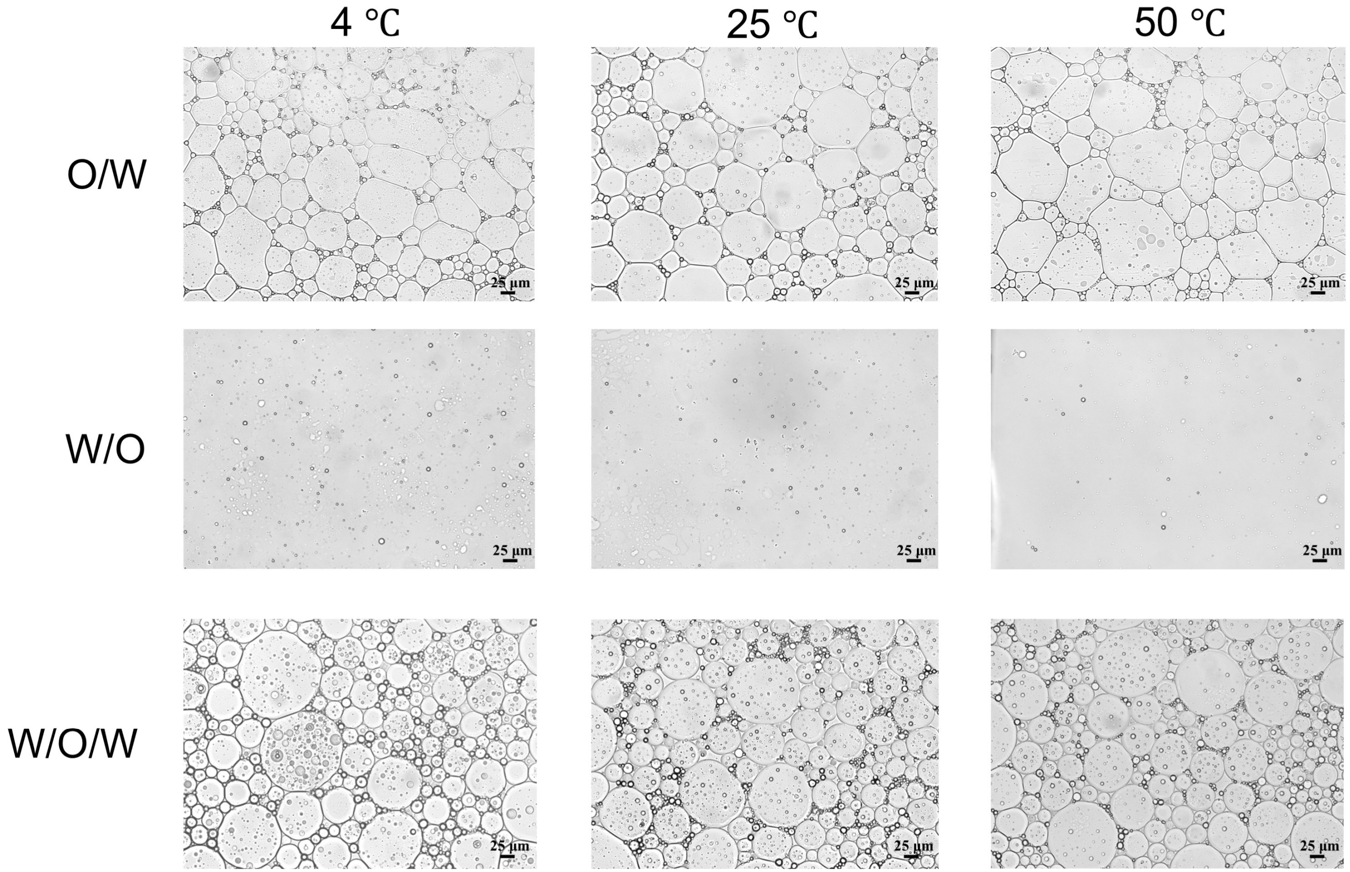

Figure S12. Temperature stability of the emulsions, scale bar=25 μm.

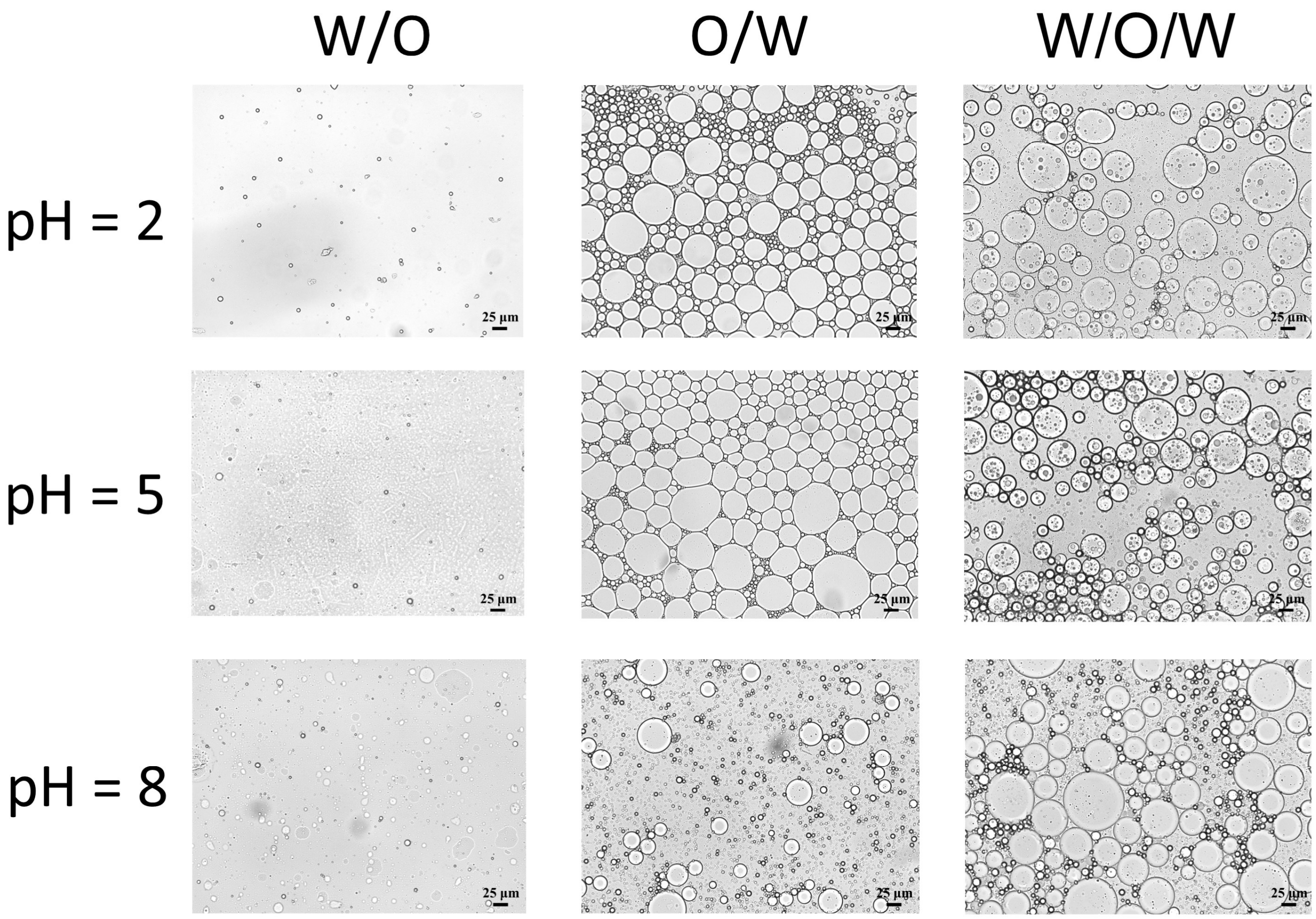

Figure S13. pH stability of the emulsions, scale bar=25 μm.

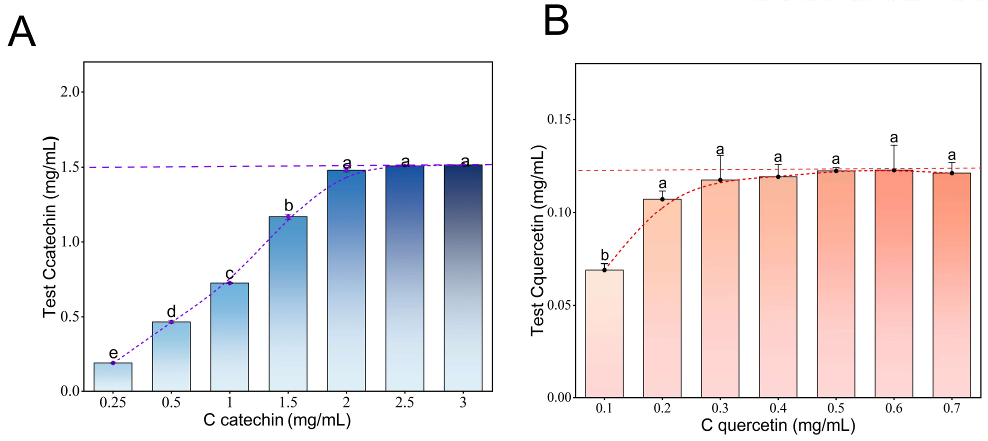

Figure S14. Solubility of Catechin and Quercetin. A) Solubility of Catechins. B) Solubility of Quercetin. Different letters represent significant differences between the two groups (n=3).

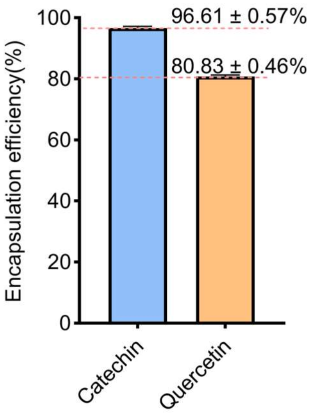

Figure S15. Encapsulation rate of Catechins and Quercetin (n=3).

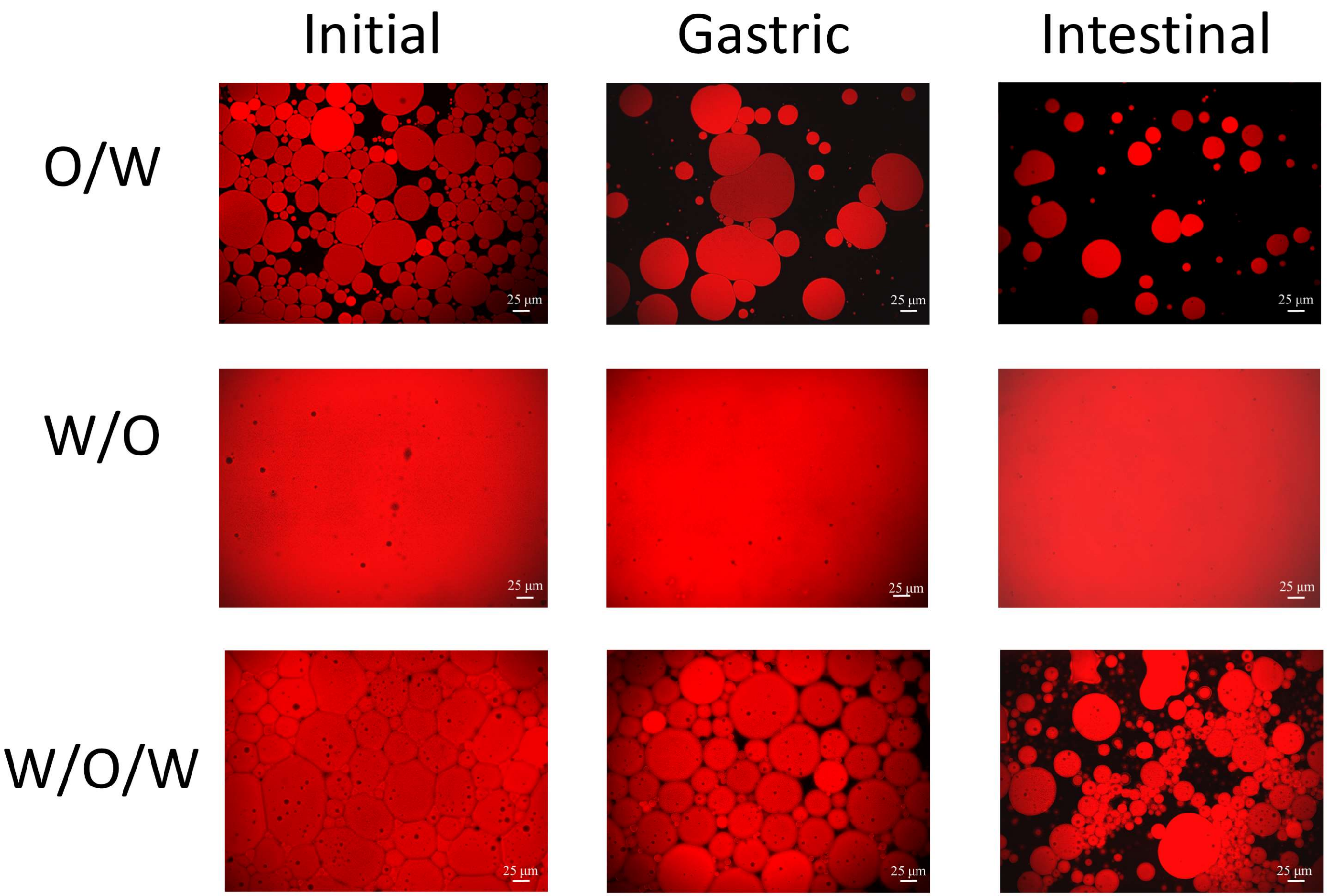

Figure S16. Different emulsions under laser confocal (Nile red stained oil phase), scale bar=25 μm.

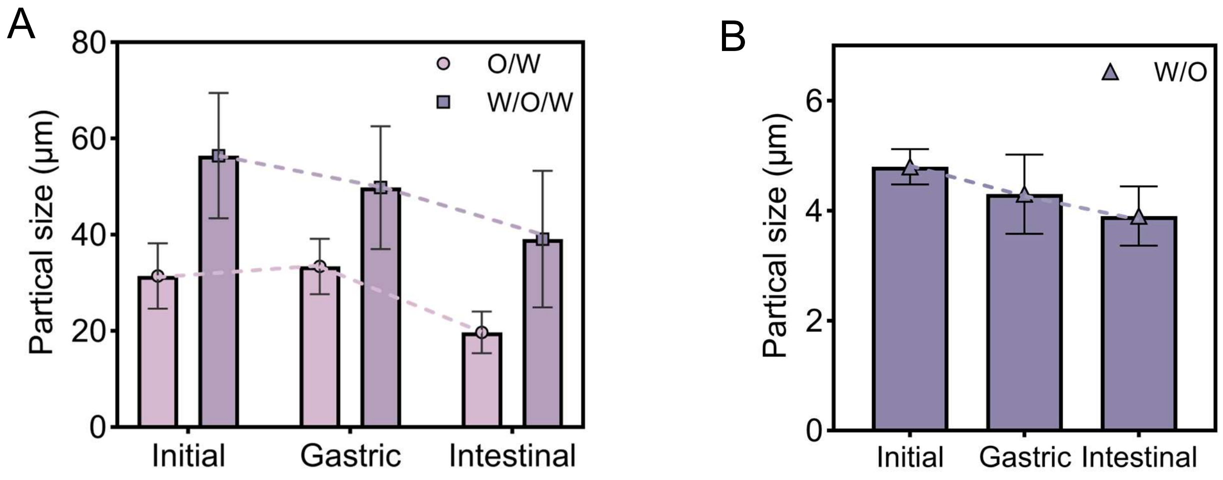

Figure S17. The particle size of different emulsions across digestive stages A). The particle size of O/W and W/O/W. B) The particle size of W/O.

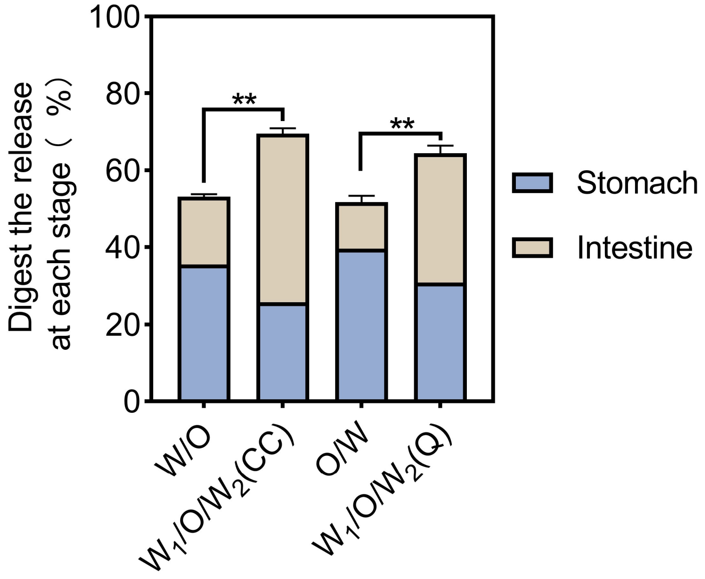

Figure S18. The release content of Catechin and Quercetin at different digestion stages, n=3.

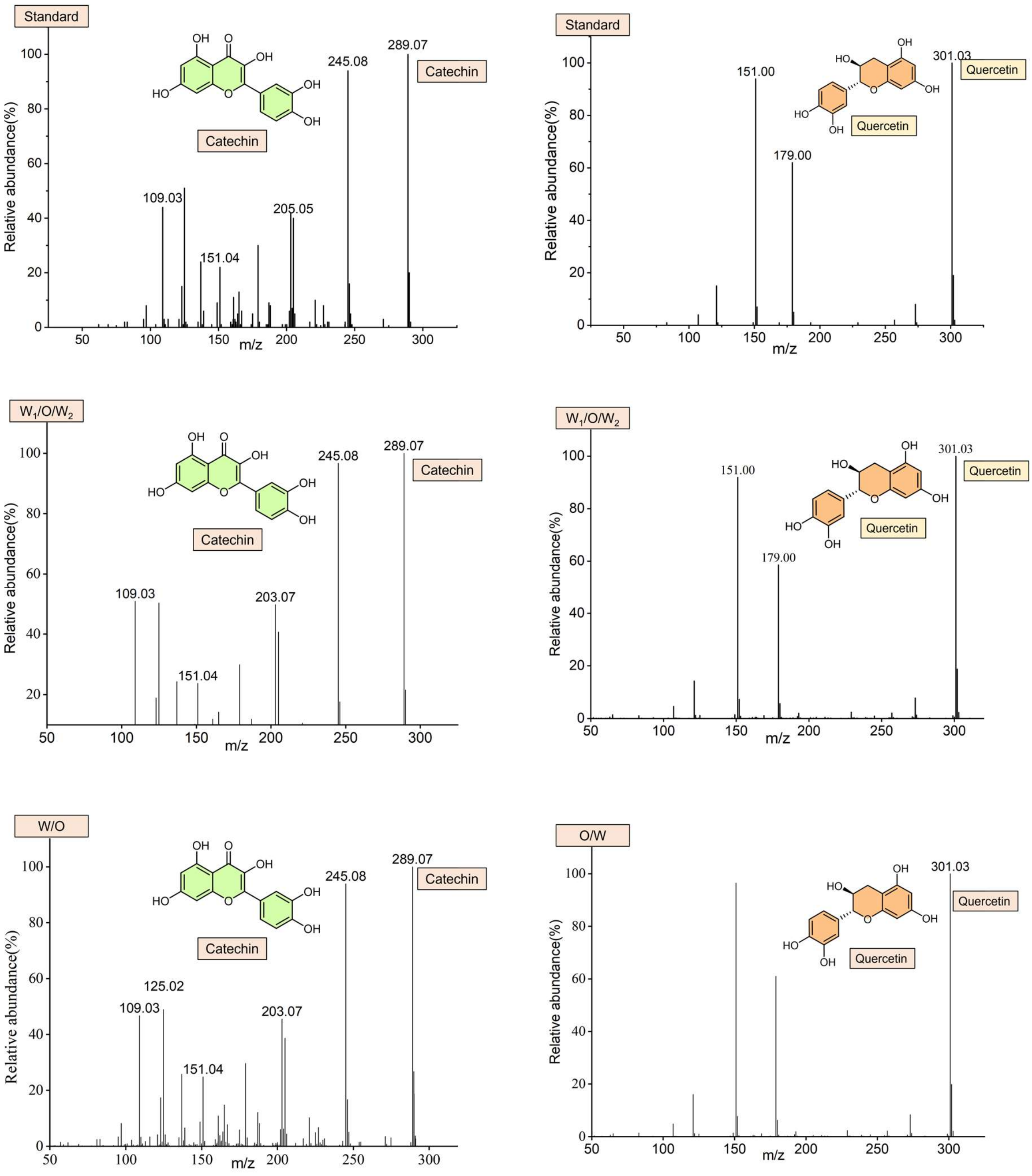

Figure S19. Secondary mass spectrometry of Catechin/Quercetin release rate in different lotion systems.

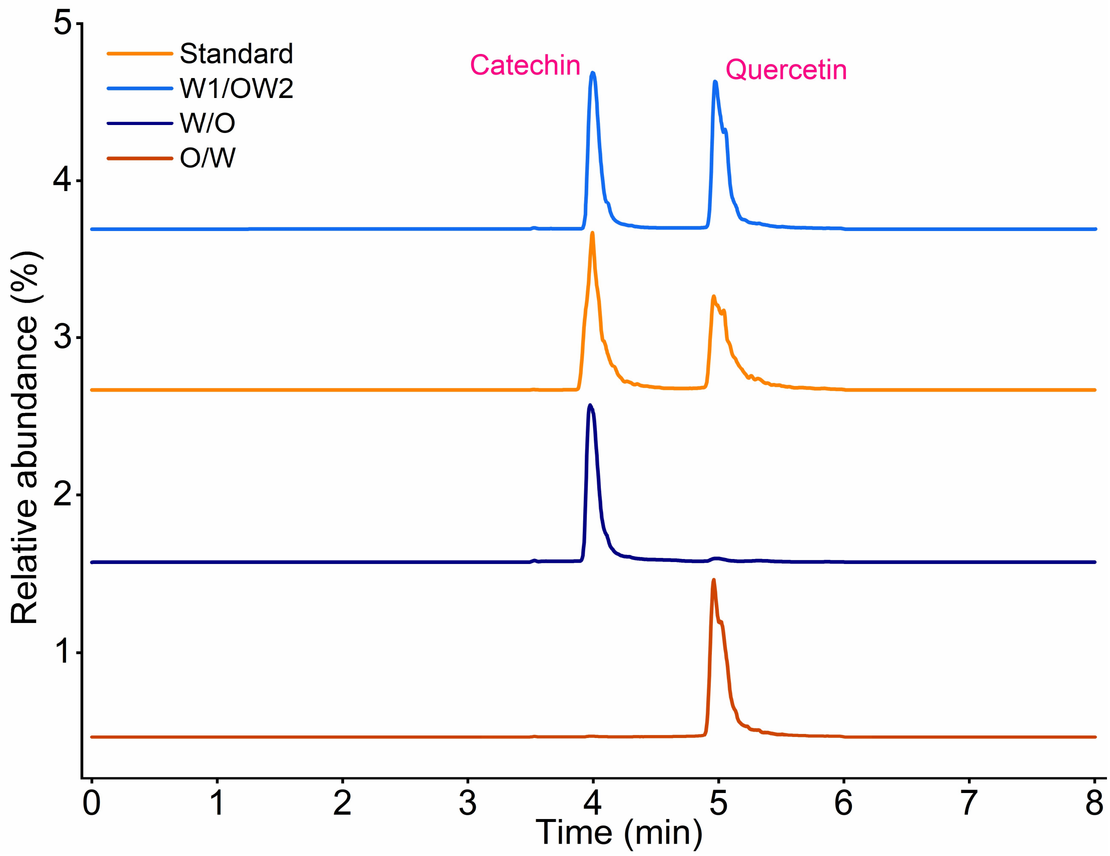

Figure S20. Total ion current diagram.

A

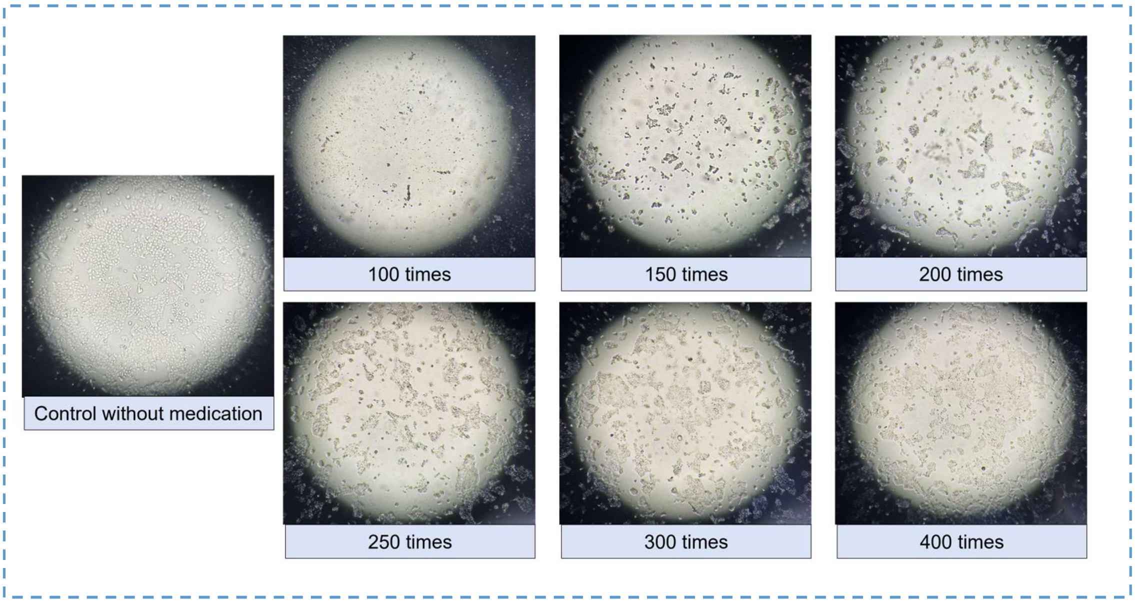

B

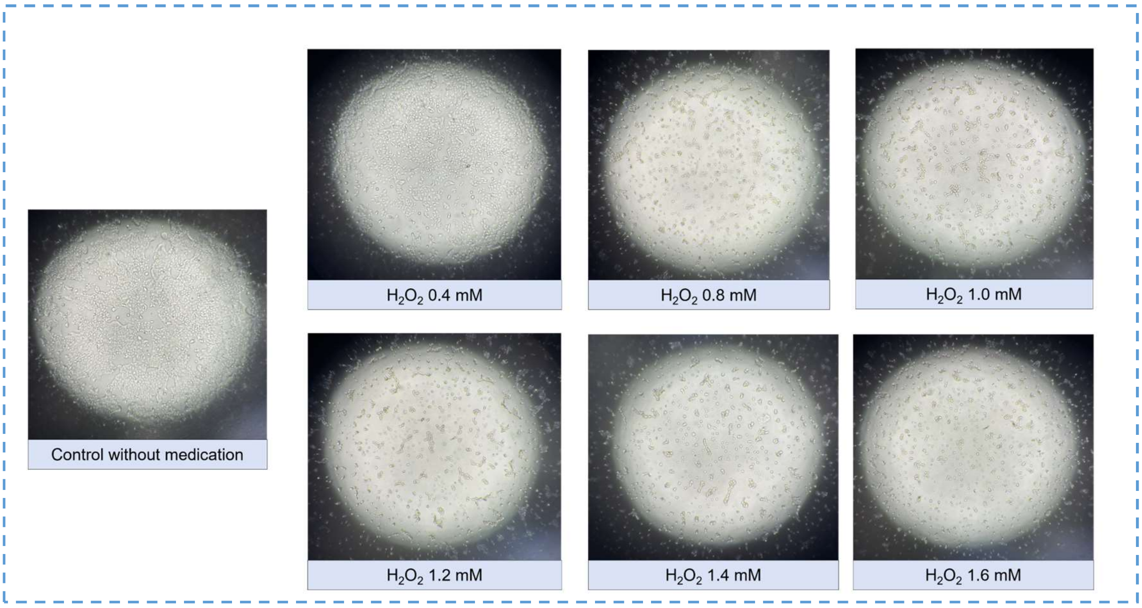

Figure S21. Microscope diagramas of Caco-2 Cells. A) Effects of Catechin/Quercetin@W_1/O/W_2 emulsion on cell viability. B) Effects of Catechin/Quercetin@W_1/O/W_2 emulsion on H_2O_2 induced cytotoxicity.

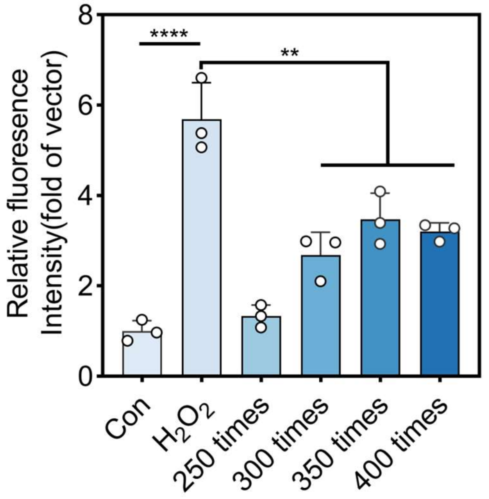

Figure S22. ROS relative fluorescence intensity (n=3) *P < 0.05, **P < 0.01, ***P < 0.001, ****P < 0.0001.

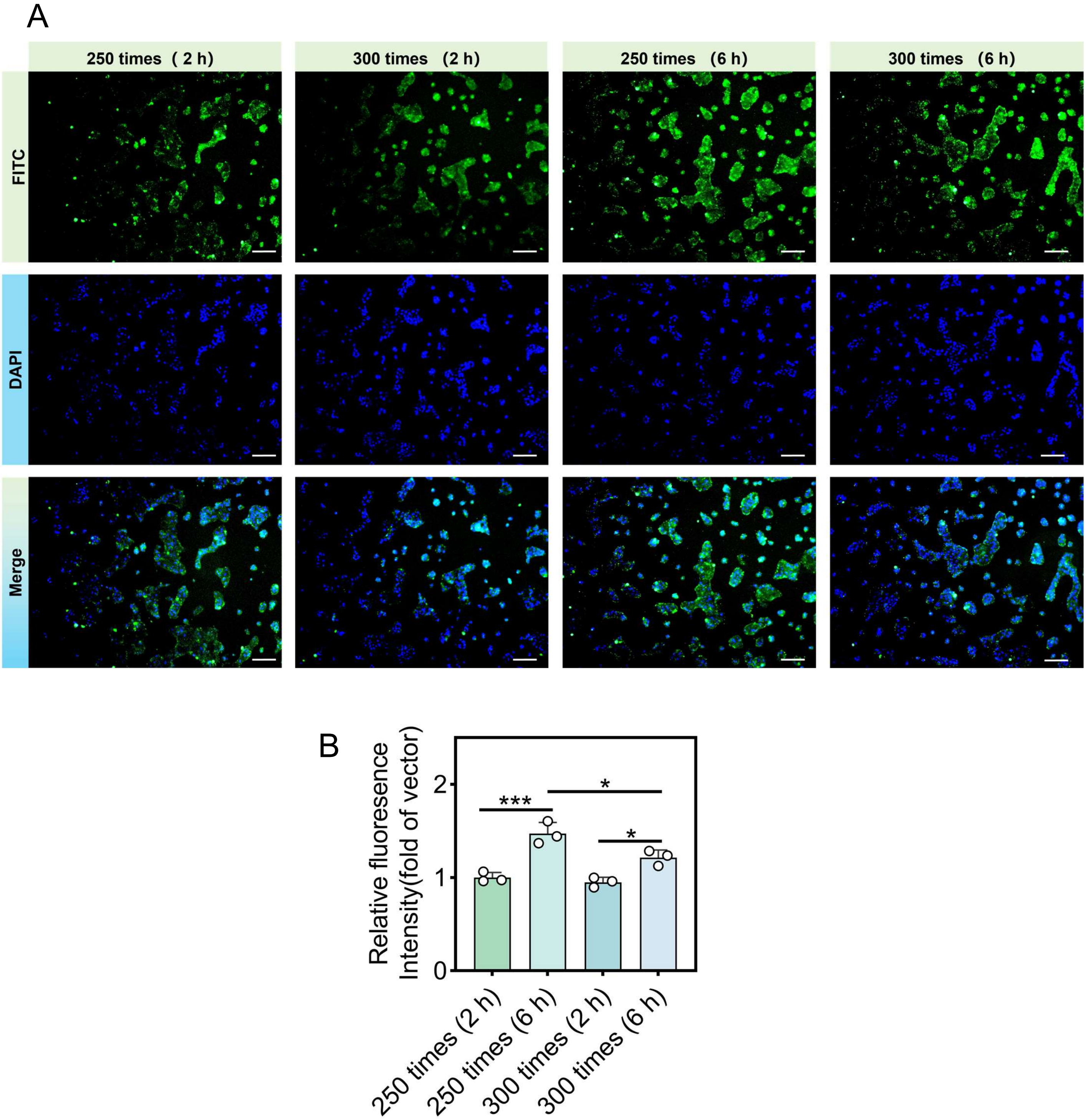

Figure S23. Quantitative analysis of cell uptake. A) Fluorescence microscopy image of cell uptake under 10 x magnification. B) Quantitative analysis of cellular uptake fluorescence intensity. *$P < 0.05$, **$P < 0.01$, ***$P < 0.001$, ****$P < 0.0001$. (scale bar=50 μm).

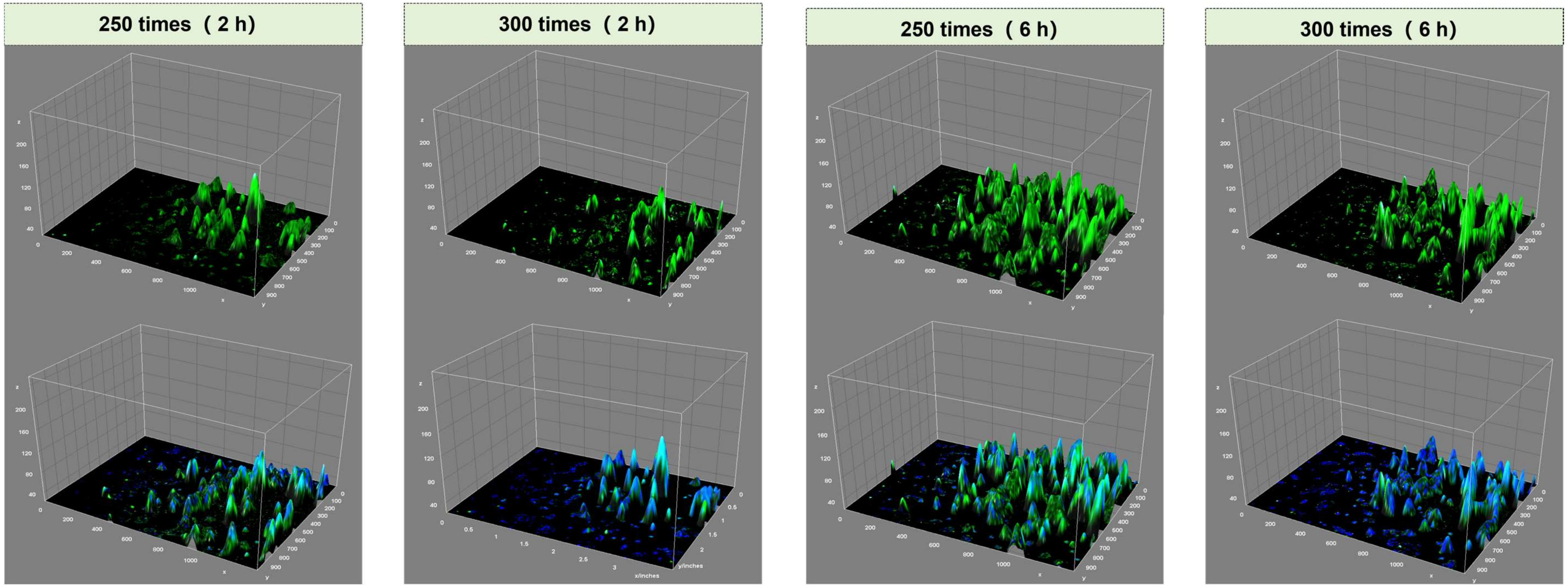

Figure S24. Fluorescence intensity distribution of different groups under low power microscope (green represents FITC, blue represents DAPI).

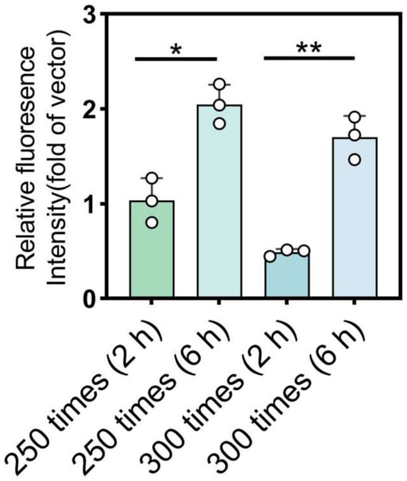

Figure S25. Quantitative analysis of cell uptake. Relative fluorescence intensity of polyphenol uptake by cells (n=3). **P* < 0.05, ***P* < 0.01, ****P* < 0.001, *****P* < 0.0001.

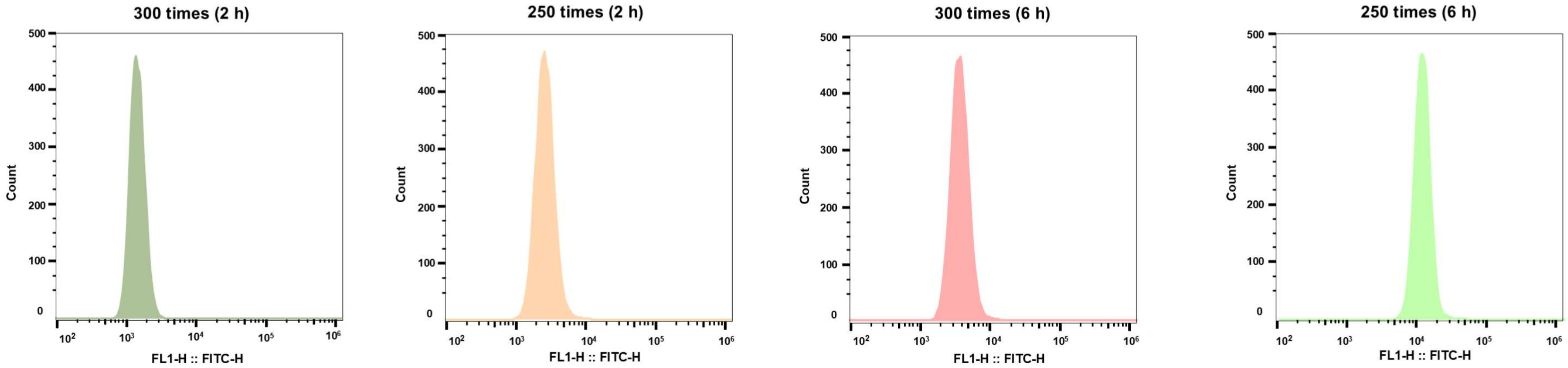

Figure S26. Quantitative detection of cellular uptake by flow cytometry using FITC labeling in each group.

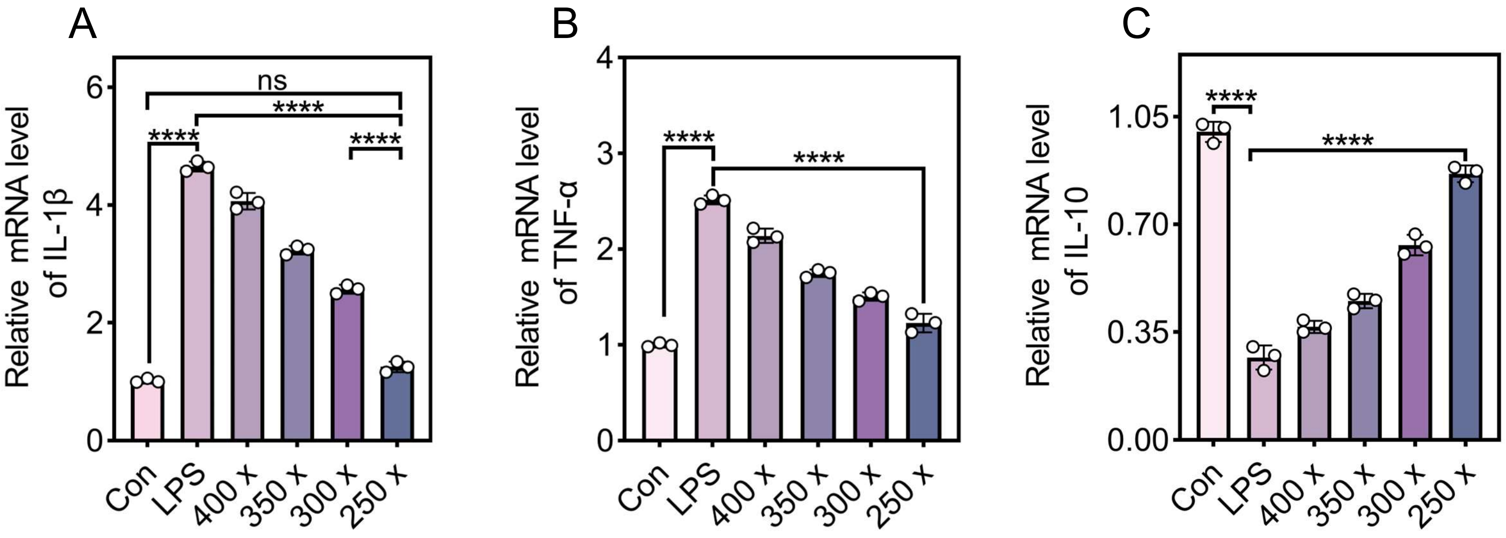

Figure S27. Effect of Catechin/Quercetin@$W_1/O/W_2$ LCC emulsion on inflammatory factors in Caco-2 cells. A-C) RT-qPCR analysis of inflammatory factors IL-10, TNF-α, and IL-1β.

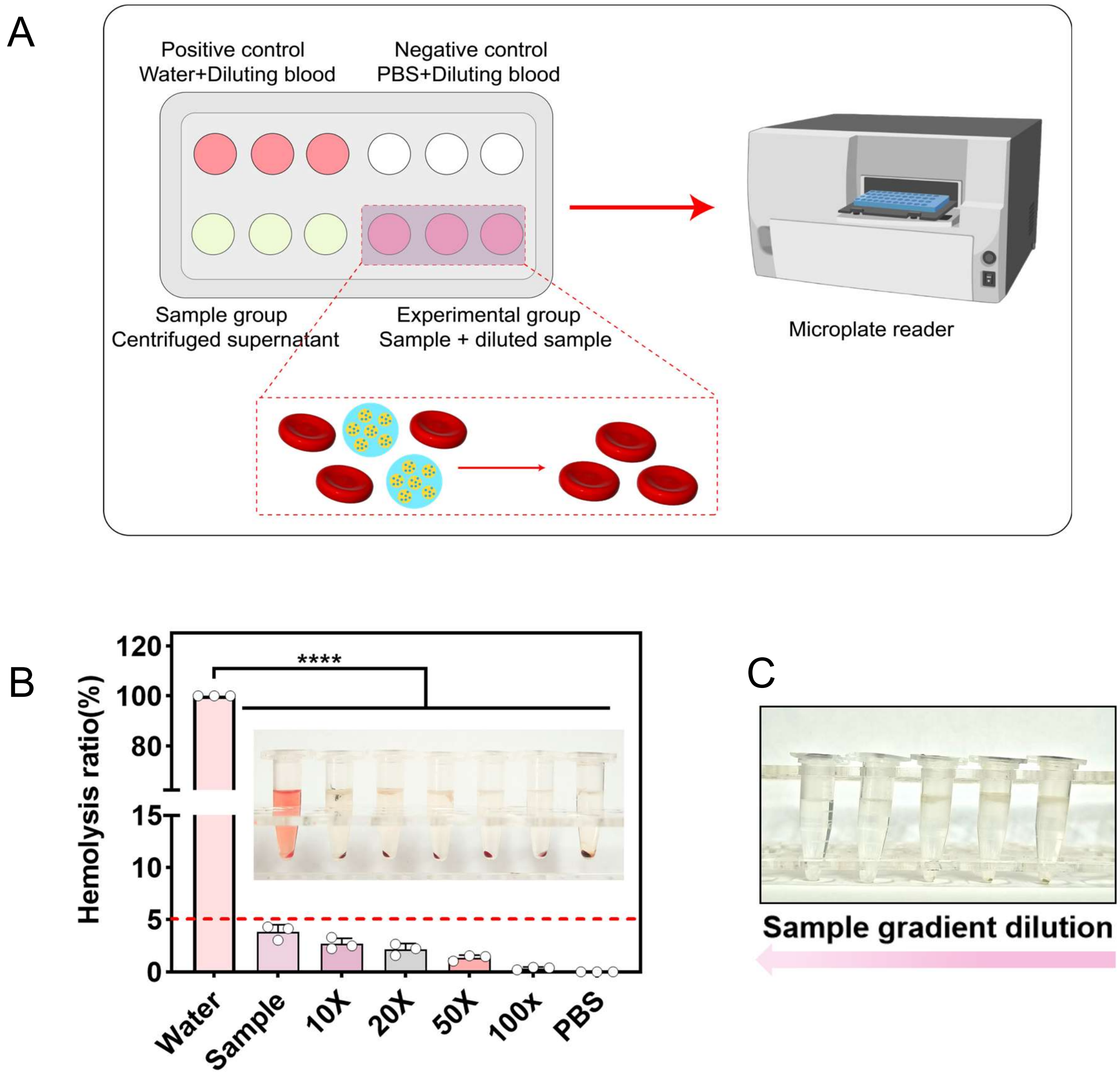

Figure S28. Biocompatibility. A) Schematic diagram of blood compatibility. B) Macrograph of blood compatibility and hemolysis. C) Macro graph of sample dilution. *$P < 0.05$, **$P < 0.01$, ***$P < 0.001$, ****$P < 0.0001$, VS Water group (n=3).

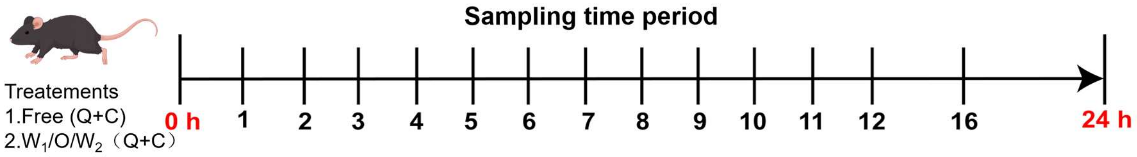

Figure S29. Schematic diagram of experimental design for 24-hour fecal excretion collection in mice.

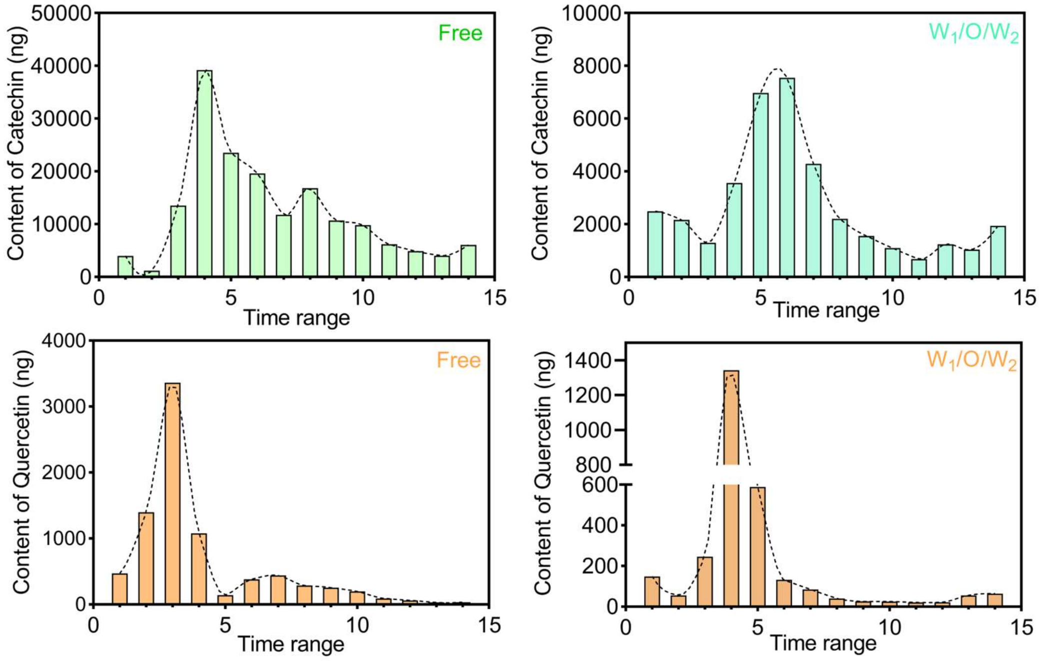

Figure S30. Content of Catechin and Quercetin in 24-hour feces of mice after different treatments (Free: Free Catechin/Quercetin, $W_1/O/W_2$: Catechin/Quercetin@$W_1/O/W_2$ LCC emulsion).

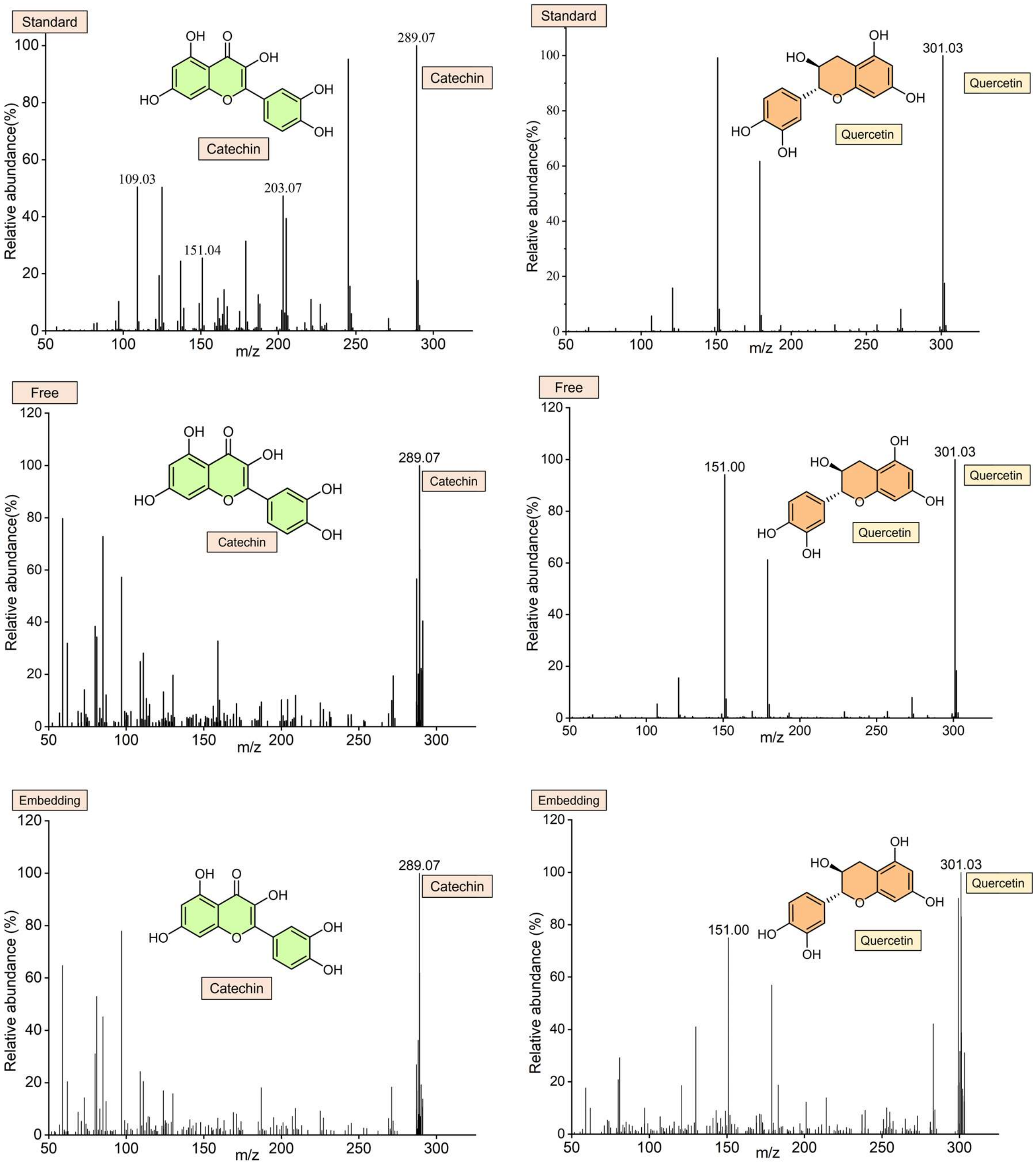

Figure S31. Secondary mass spectrometry of free Catechin, Quercetin, $W_1/O/W_2$ emulsion digested in mice.

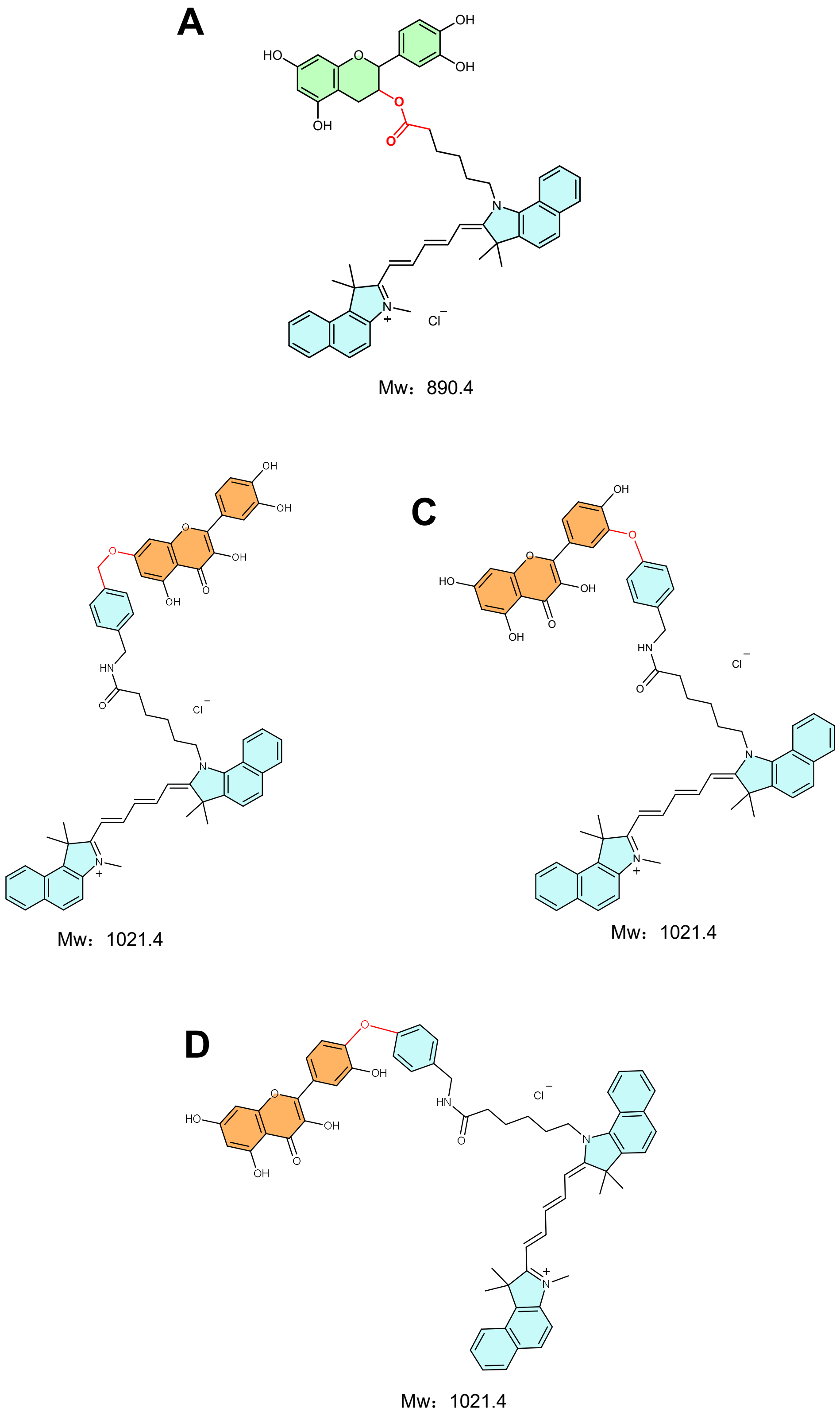

Figure S32. CY5.5-CC, CY5.5-Q structures and their isomers. A) Structure of CY5.5-CC and its molecular weight. B-D) CY5.5-Q structure and its molecular weight.

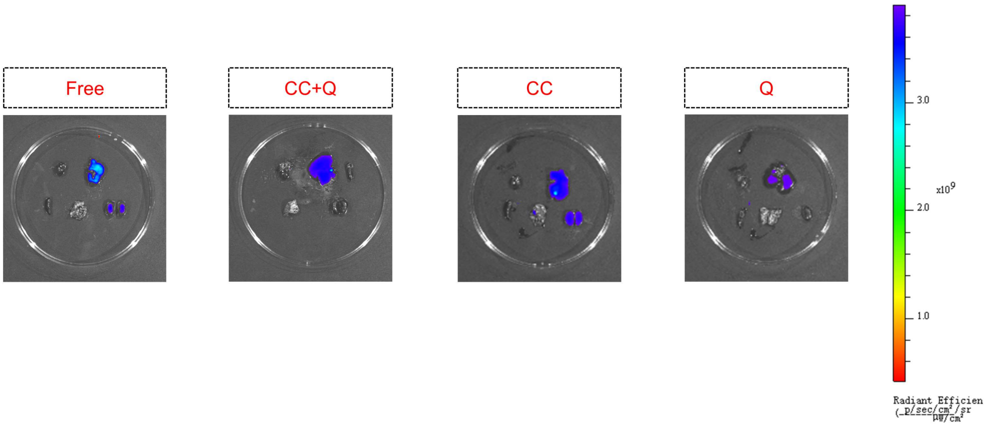

Figure S33. Fluorescence images of mice in various groups. Intensity analysis of fluorescence for mouse organs in each group after 48 hours of oral administration.

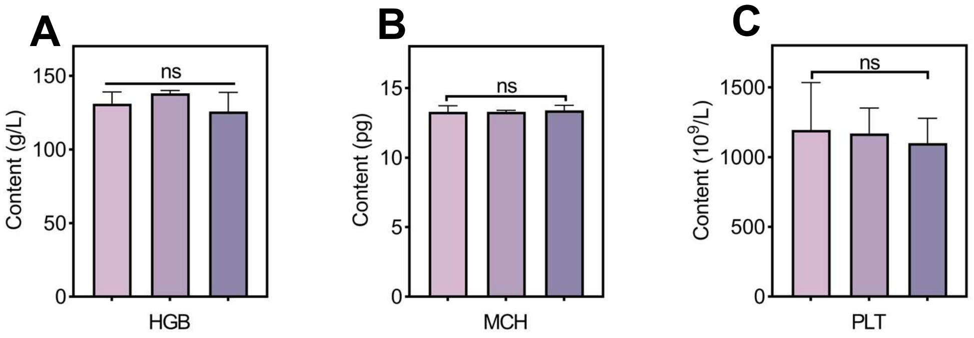

Figure S34. Safety of Catechin/Quercetin@W_1/O/W_2 emulsion in vivo organisms. (A-C) Blood routine indexes of mice in different groups *$P < 0.05$, **$P < 0.01$, ***$P < 0.001$, ****$P < 0.0001$, VS Water group (n=3).

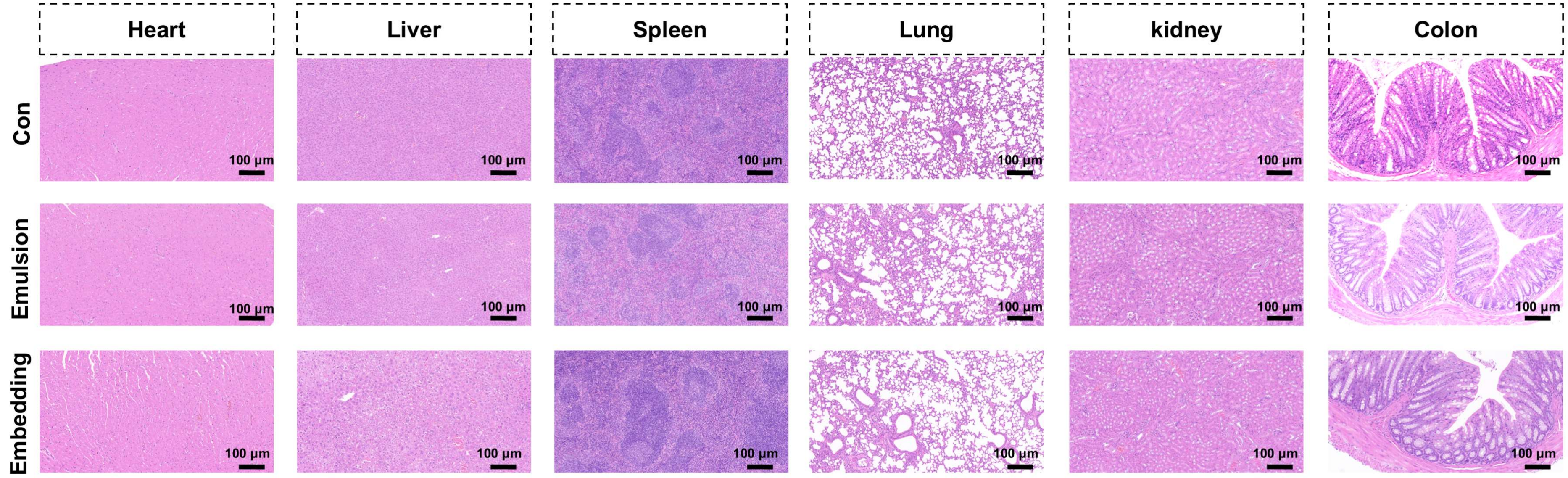

Figure S35. Biosecurity. H&E staining of various organs (Heart, Liver, Spleen, Lung, Kidney and Colon) of mice in different groups, scale bar=100 μm (n=3).

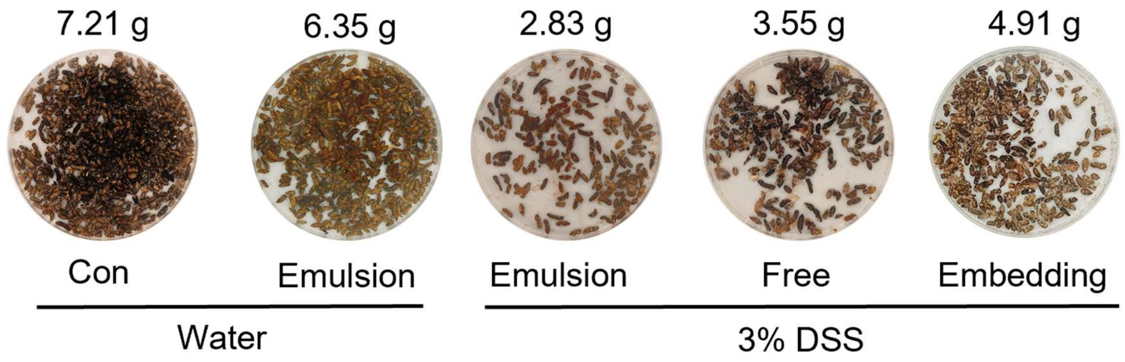

Figure S36. Fecal volume of mice in different groups during 24 h.

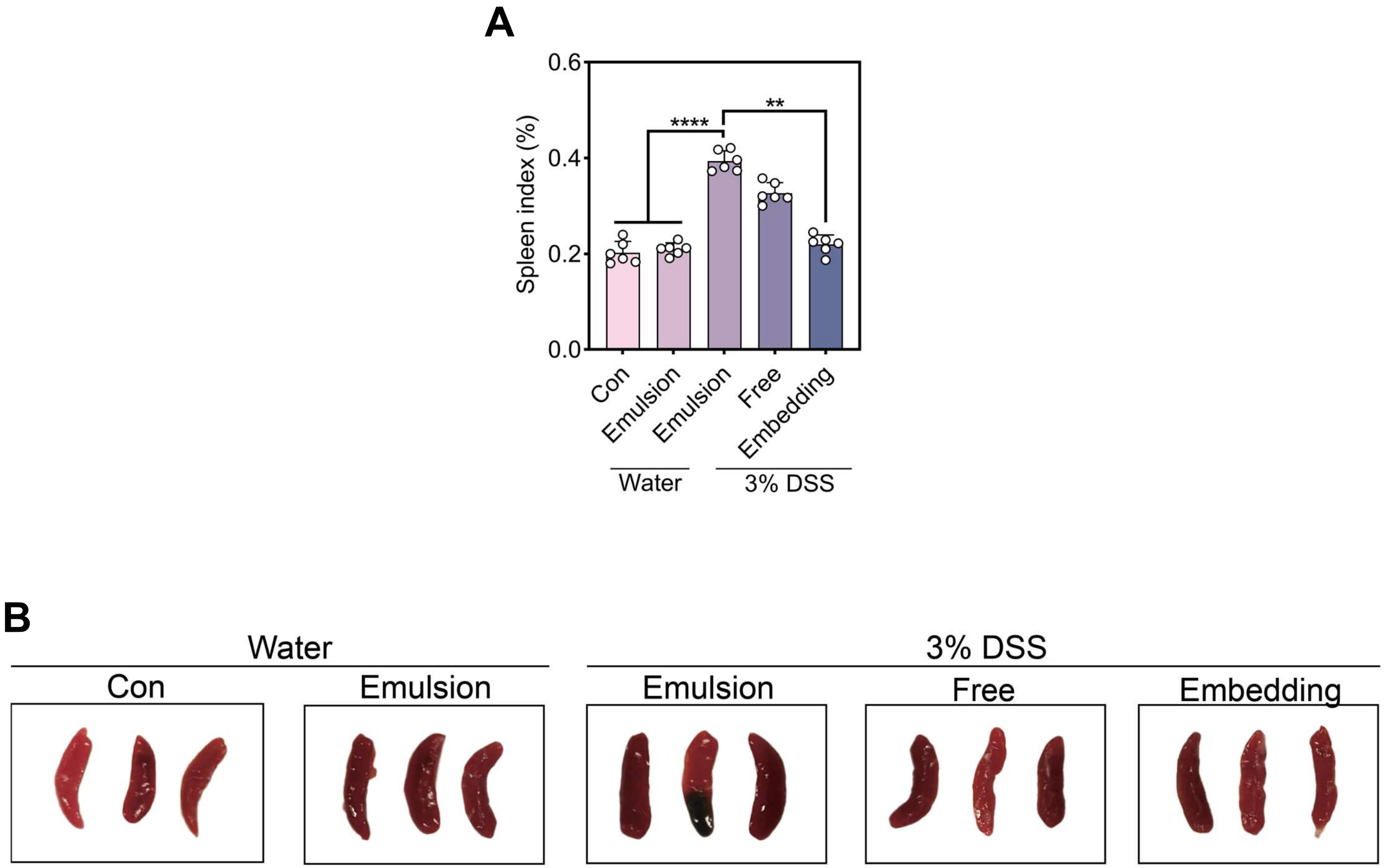

Figure S37. Splee-related paraments. A) Spleen index. B) Macrograph of spleen in different groups (n=6), $*P < 0.05$, $**P < 0.01$, $***P < 0.001$, $****P < 0.0001$.

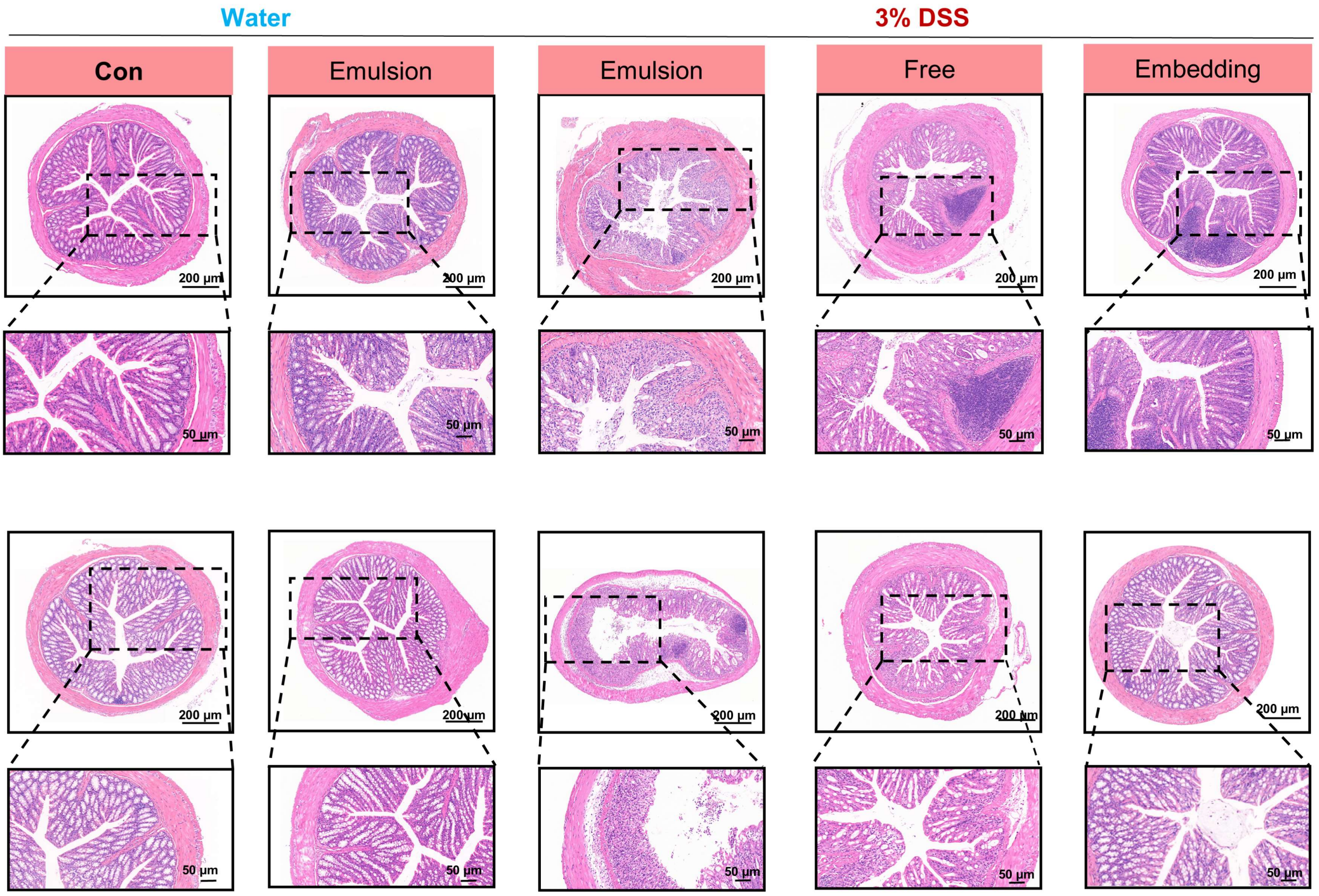

Figure S38. Pathological analysis of H&E in colon tissues of mice in different groups. (scale bar=200 μm, 50 μm).

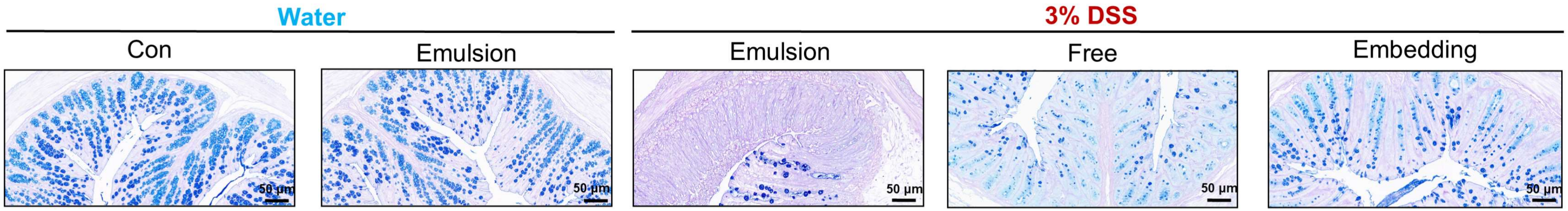

Figure S39. Colon AB-PAS staining (scale bar=50 µm).

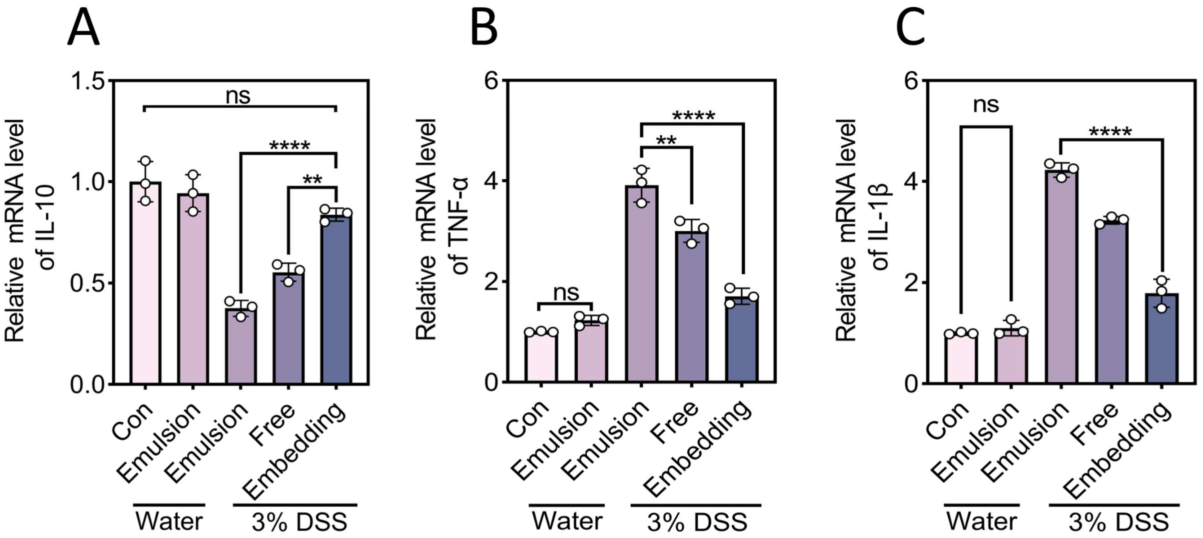

Figure S40. Relative mRNA expression levels of inflammatory cytokines in colon of different mice. A) Relative mRNA expression levels of IL-10. B) Relative mRNA expression levels of TNF-α. C) Relative mRNA expression levels of IL-1β) (n = 3).

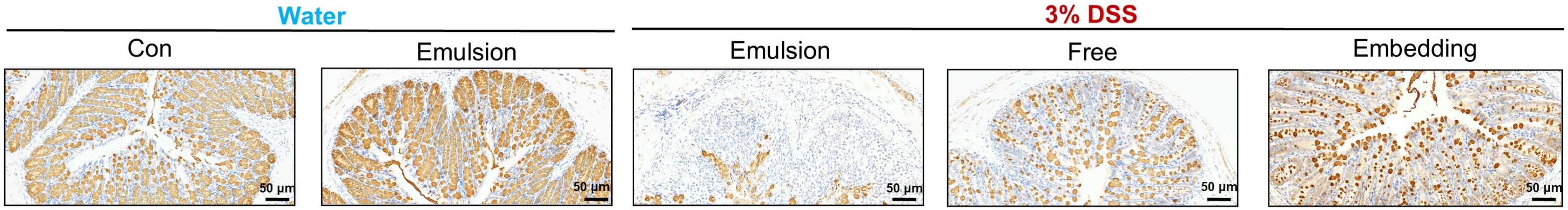

Figure S41. Immunohistochemistry of MUC-2 (scale bar=50 μm).

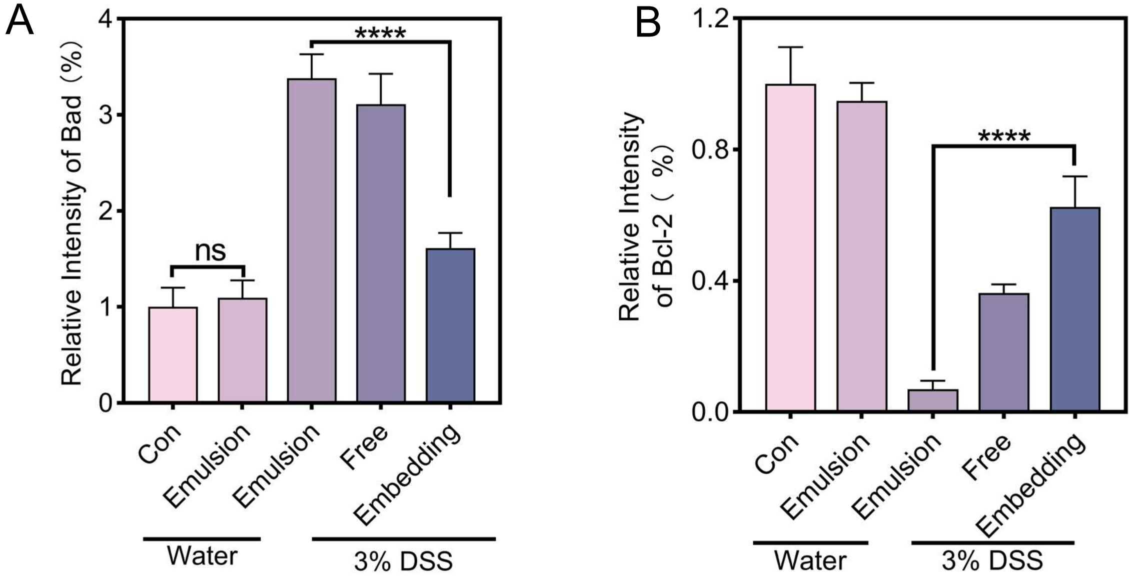

Figure S42. The expression of apoptosis proteins (Bad, Bcl-2) in different groups of mice. A) Pro apoptotic protein (Bad) expression. B) Anti apoptotic protein (Bcl-2) expression. *$P < 0.05$, **$P < 0.01$, ***$P < 0.001$, ****$P < 0.0001$ (scale bar = 50 μm).

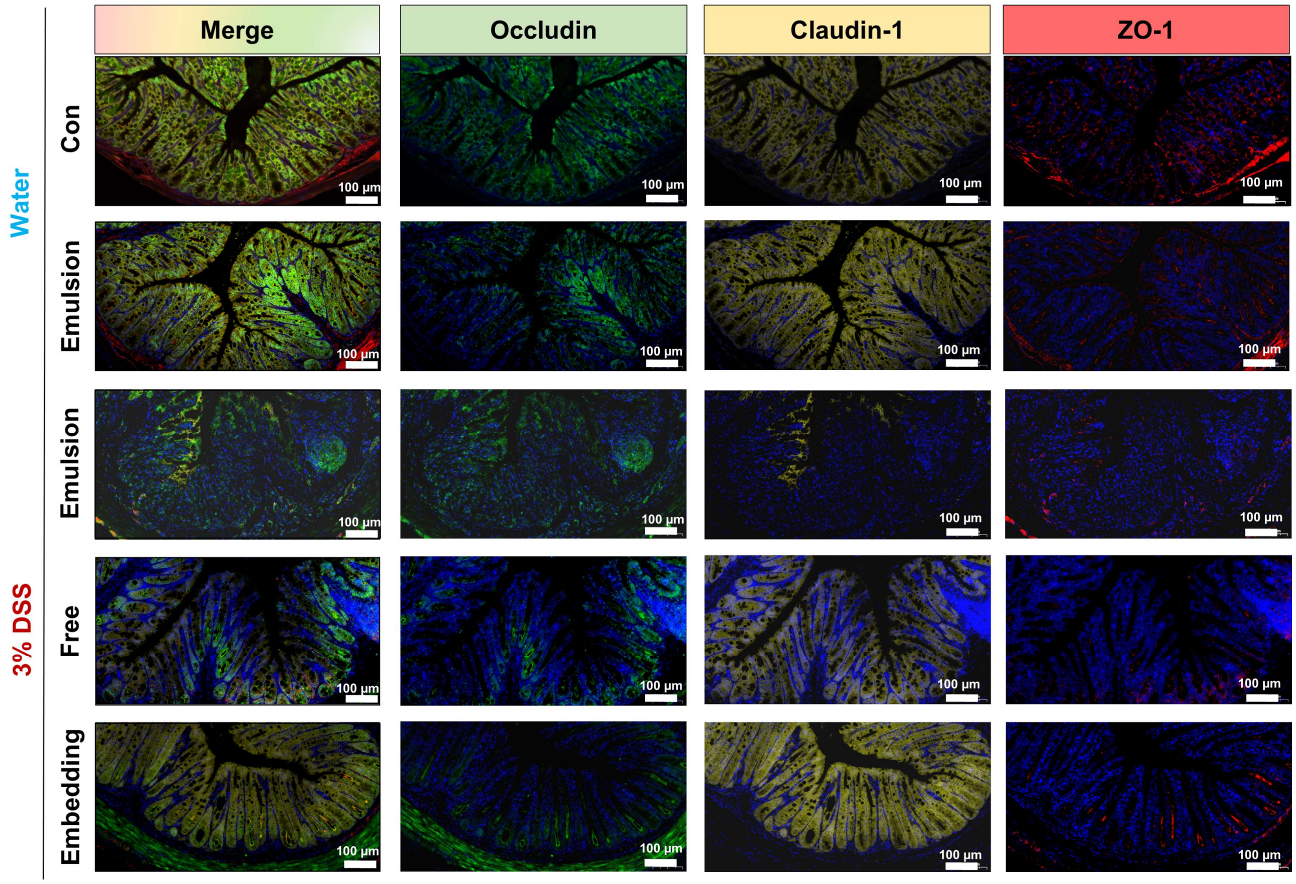

Figure S43. Immunofluorescence analysis of colonic tight junction proteins in different groups of mice. Green represents Occludin, orange represents Claudin-1, and red represents ZO-1, scale bar=100 μm.

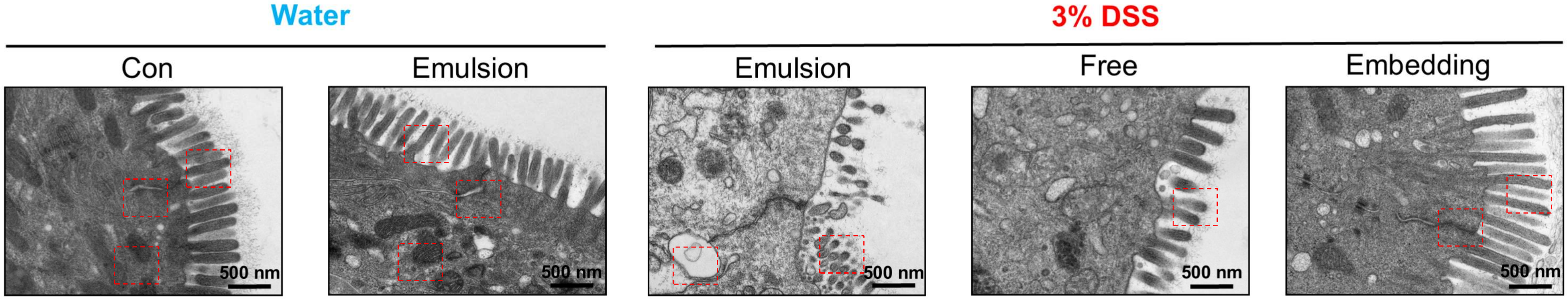

Figure S44. Ultrastructure of colon tissue epithelial (scale bar=500 nm).

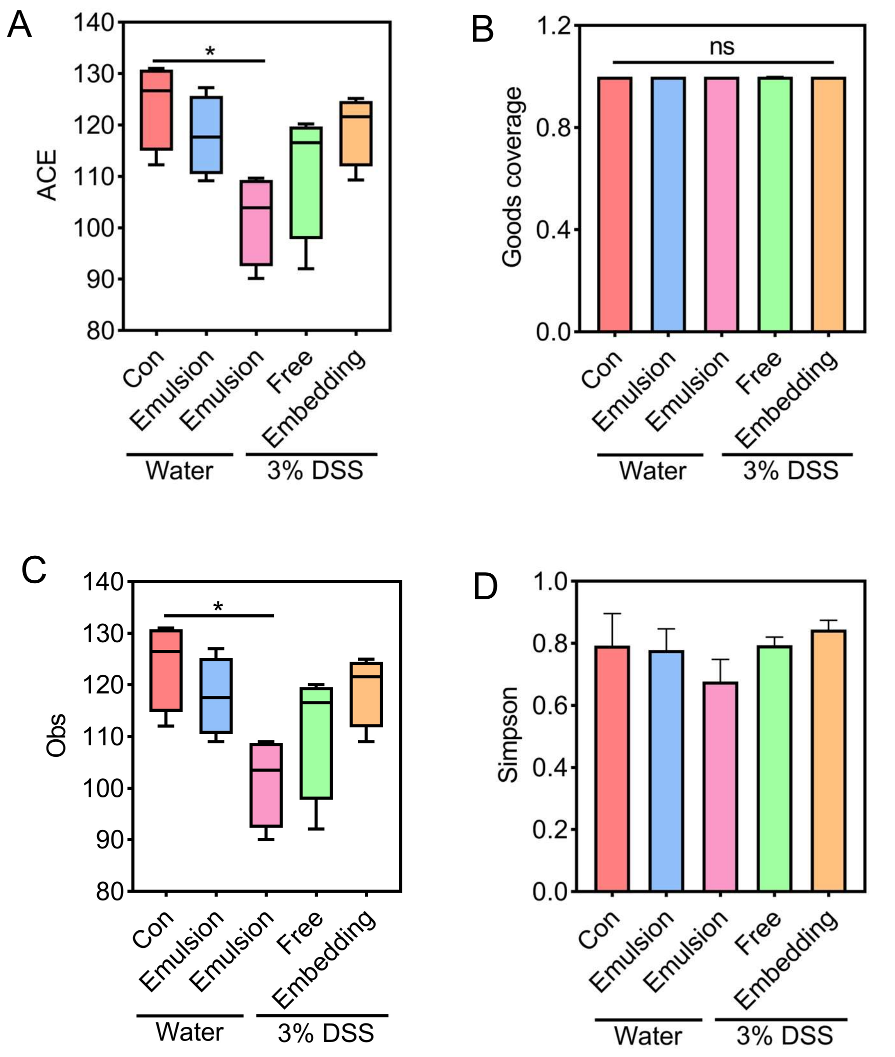

Figure S45. Intestinal microbiota α index analysis: A) ACE. B) Goods coverage. C) Observe species. D) Simpson. *$P < 0.05$, **$P < 0.01$, ***$P < 0.001$, ****$P < 0.0001$.

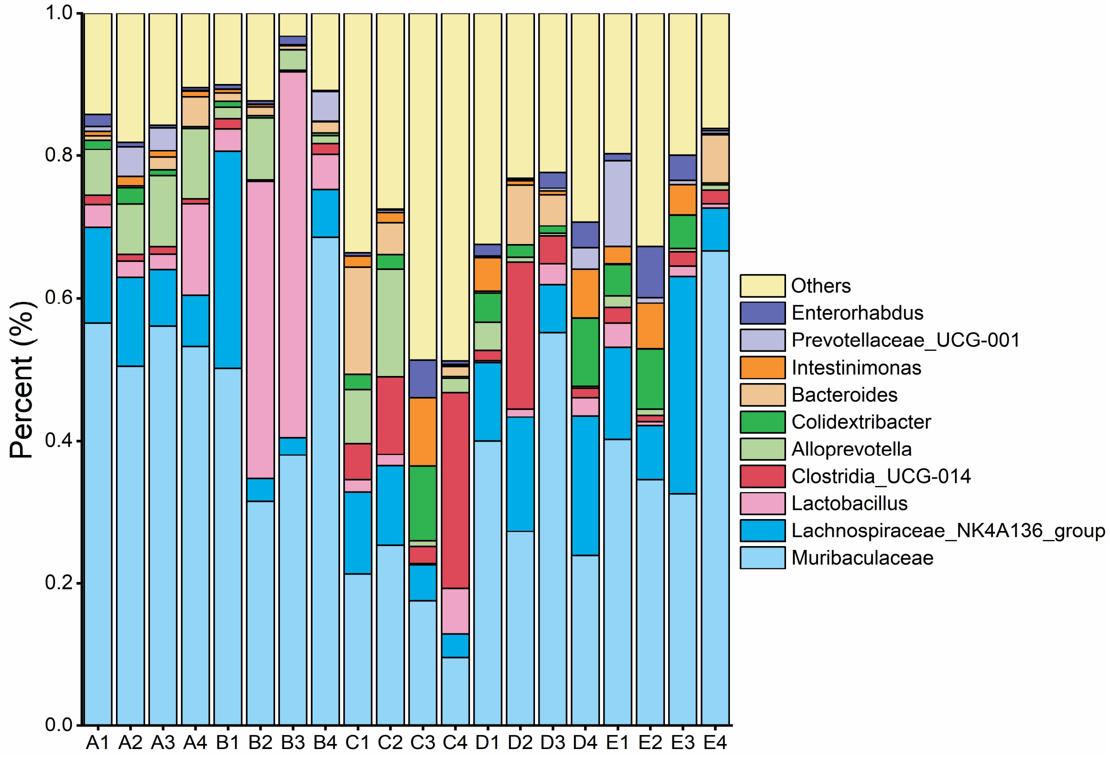

Figure S46. Relative abundance of genus-level microbiota in different groups of mice (A: Con group, B: Emulsion group, C. DSS+Emulsion group, D. DSS+Free group, E. DSS+Embedding group).

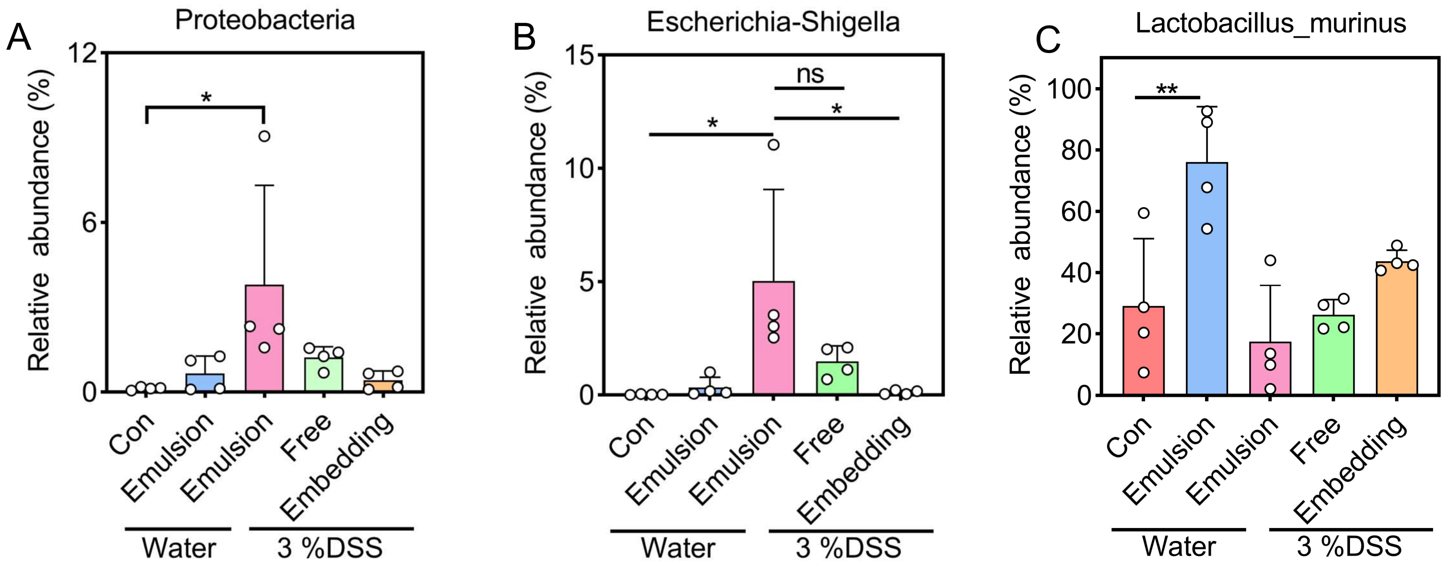

Figure S47 Relative abundance of significant bacteria. A-C) The relative abundance of *Proteobacteria*, *Escherichia-Shigella, Lactobacillus_murinus* in different groups of mice. **P* < 0.05, ***P* < 0.01, ****P* < 0.001, *****P* < 0.0001.

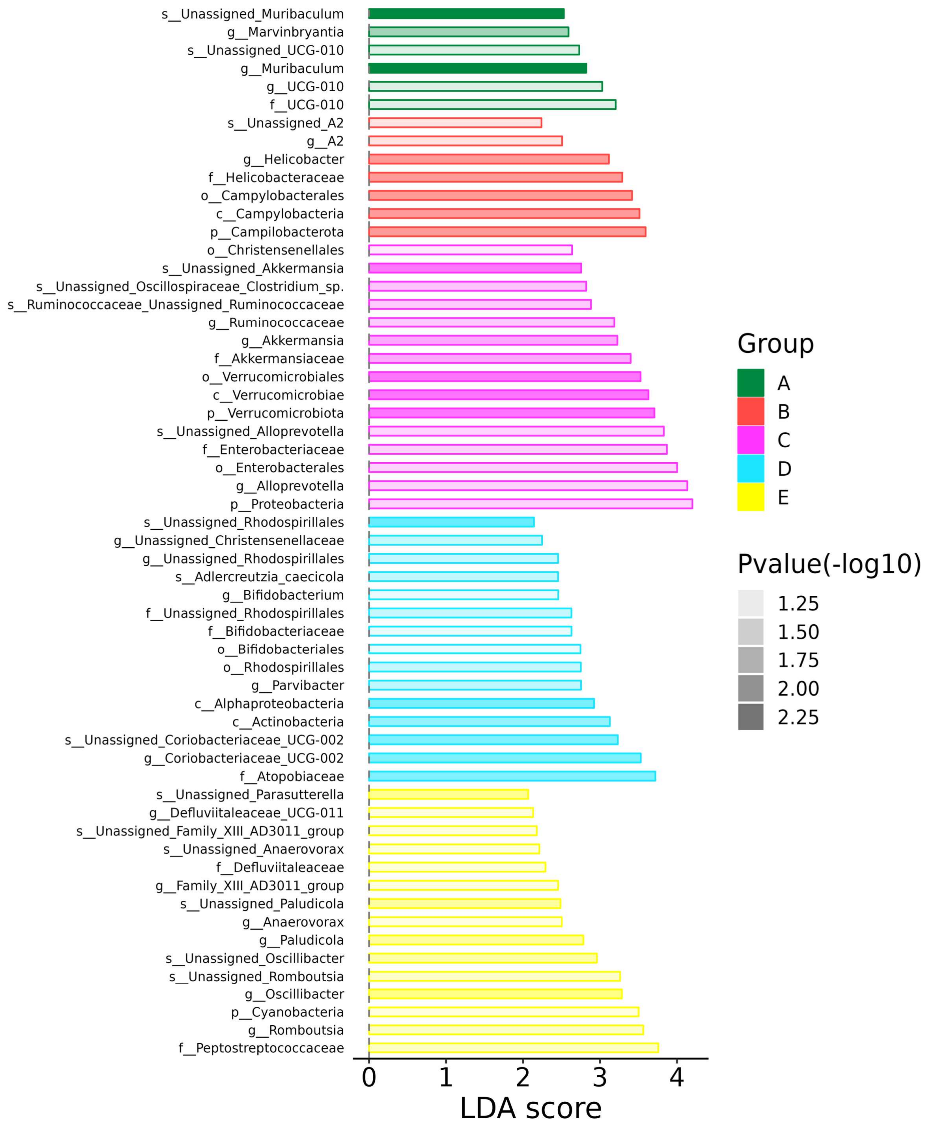

Figure S48. Linear discriminant analysis (LEfse) analysis of different taxonomic levels from phylum to species.

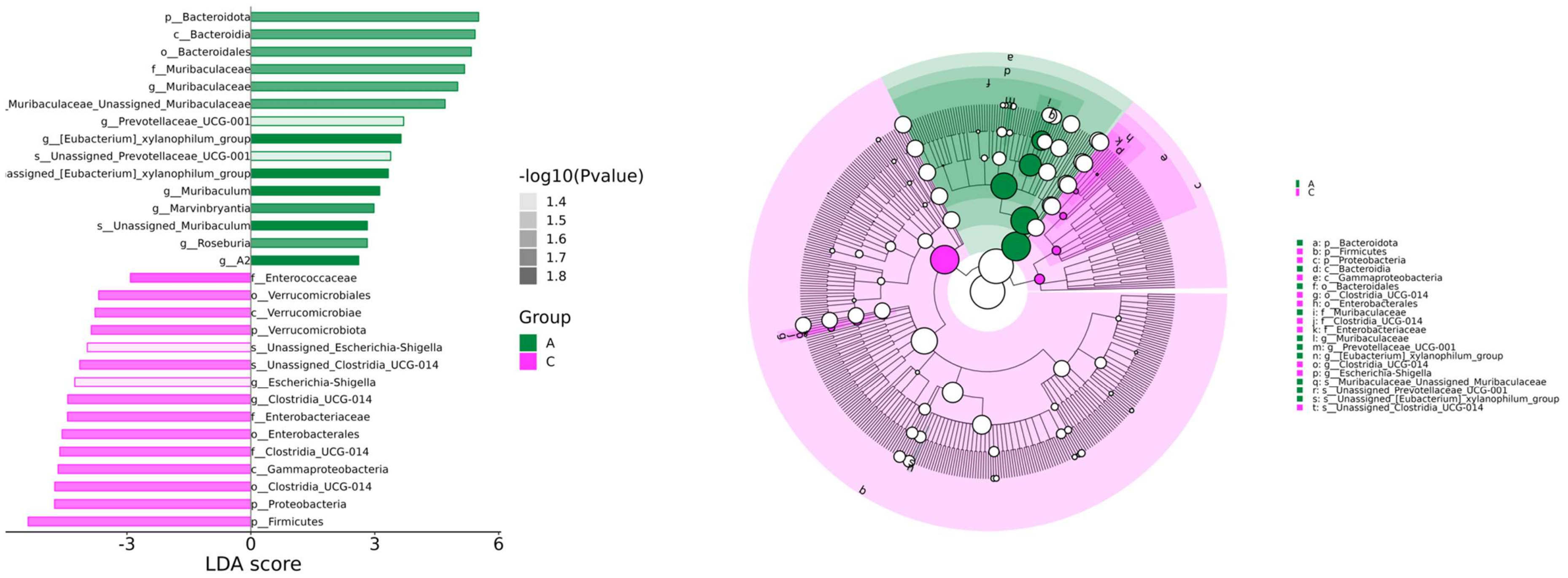

Figure S49. Linear discriminant analysis (LEfse) analysis of different taxonomic levels from phylum to species. (A: Con group VS C: DSS group).

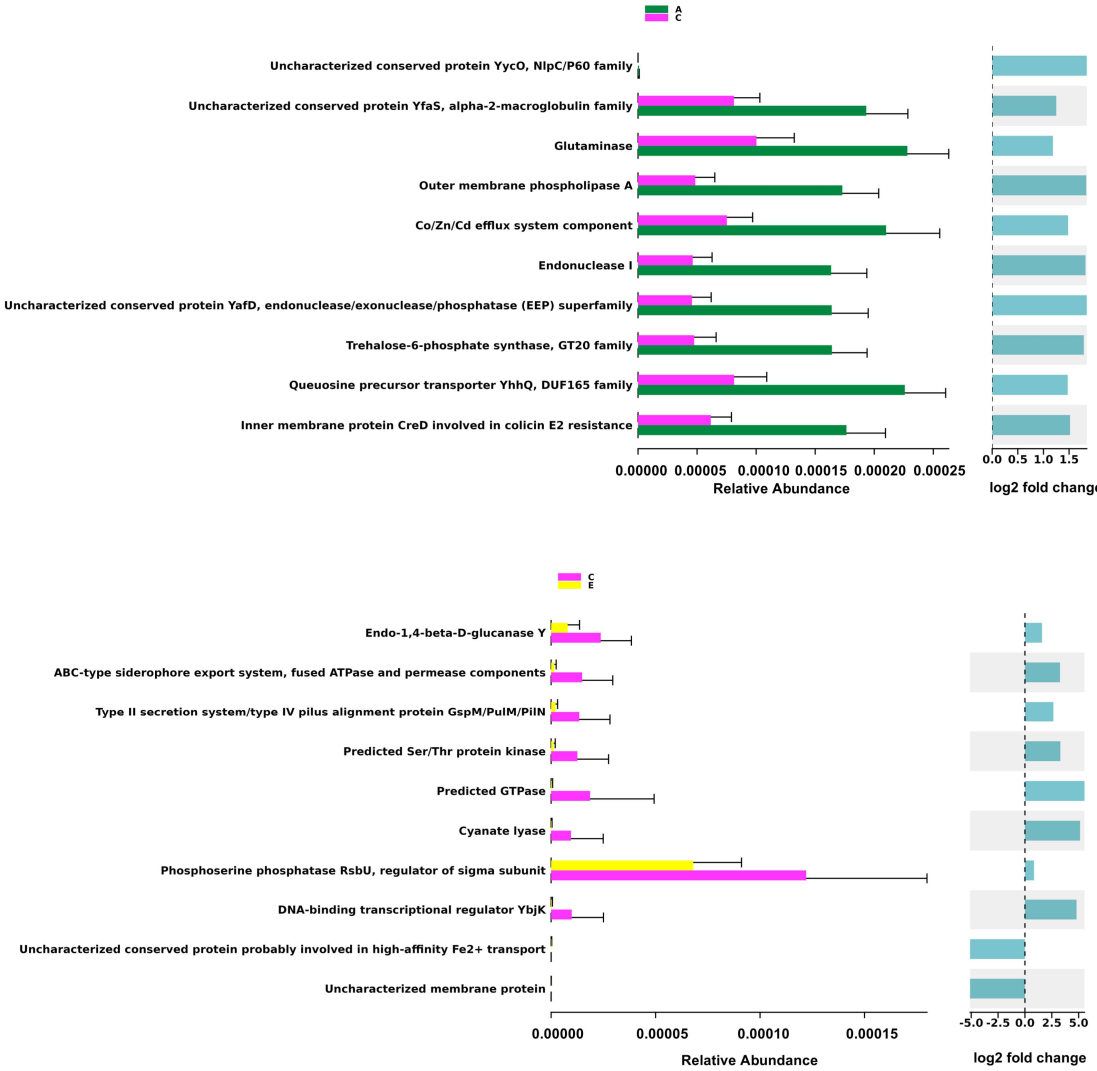

Figure S50. COG functional enrichment related to DSS-induced gut microbiota after drug intervention. A: the con group; C: the DSS group; E: the embedding group.

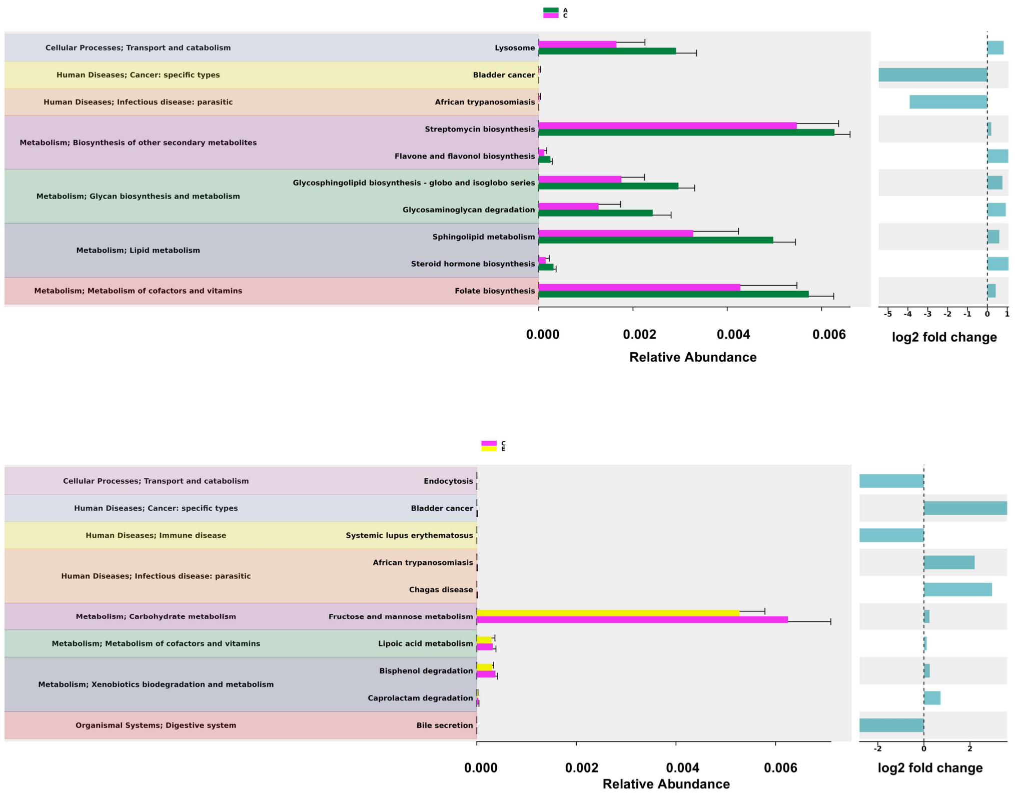

Figure S51. KEGG functional enrichment related to DSS-induced gut microbiota after drug intervention. A: the con group; C: the DSS group; E: the embedding group.

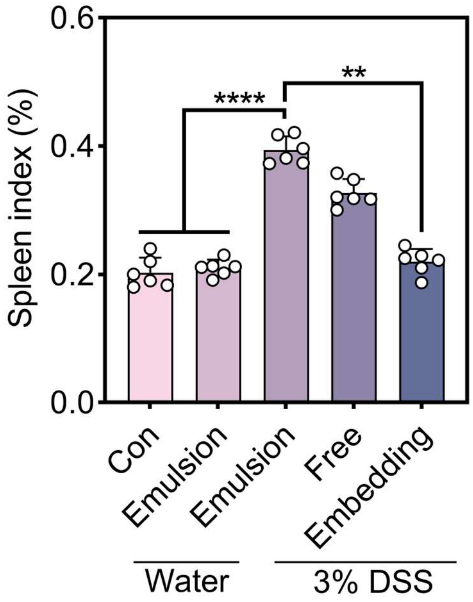

Figure S52. Spleen index of mice in different groups (n=6).

Table S1. Molecular weight before and after LCC modification

Samples	Mw×104 (Da)	Mw/Mn
LCC	2.26±0.01a	2.09
Modified LCC	4.18±0.02b	2.16

Table S2. Chemical composition of LCC and modified LCC

Chemicalcompositions	LCC	Modified LCC
Glucose	31.01	12.25
Mannose	18.84	5.96
Xylose	14.63	2.61
Galactose	10.66	3.48
Arabinose	2.53	0.23

Table S3. Assignments of ^{13}C–^{1}H cross-signals in the 2D HSQC NMR Spectra of LCC, Modified LCC.

Lable	δC/δH (ppm)	Assignment	LCC	Modified LCC
OCH_3	55.33/3.73	C-H in methoxyls	√	√
A_α	71.00/4.74	C_α-H_α in *β*-O-4 substructures (A)	×	√
$A_{\beta(G)}$	83.44/4.28	C_β-H_β in *β*-O-4 linked to G	√	√
A_γ	60.86/3.48-3.51	C_γ-H_γ in *β*-O-4 substructures (A)	×	√
B_α	84.67/4.58	C_α-H_α in *β*-*β*′ resinol	√	√
B_β	53.27/2.97	C_β-H_β in *β*-*β*′ resinol	√	√
B_γ	69.12-71.12/3.49-3.92	C_γ-H_γ in *β*-*β*′ resinol	×	√
C_α	86.75/5.42	C_α-H_α in phenylcoumaran	√	×
C_β	52.87/3.44	C_β-H_β in phenylcoumaran	√	√
C_γ	63.13/3.62	C_γ-H_γ in phenylcoumaran	×	√
D'_α	79.34/4.01	C'_α-H'_α in spirodienones	×	√
G_2	110.18/6.9	C_2-H_2 in guaiacyl units	√	√
G_5	114.4/6.68	C_5-H_5 in guaiacyl units	√	√
G_6	120.3/6.74	C_6-H_6 in guaiacyl units	√	√
$H_{2.6}$	127.99/7.35	$C_{2.6}$-$H_{2.6}$ in *p*-hydroxyphenyl units	√	√
Ara_5	62.90/3.53	C_5-H_5 in *α*-L-arabinofuranose	√	×
Glc_1	102.47/4.30	C_1-H_1 in *β*-D-glucopyranose	√	×

Glc_2	70.88/4.73	C_2-H_2 in *β*-D-glucopyranose	√	×
Man_3	70.53/5.19	C_3-H_3 in *β*-D-mannopyranose	√	×
Man_4	72.73/3.82	C_4-H_4 in *β*-D-mannopyranose	√	×
Gal_1	105.22/4.25	C_1-H_1 in *β*-D-galactopyranose	√	×
Est	62.88/4.21	Esters to the lignin γ-OH	√	√
BE_1	80.66/4.51	Benzyl ether	√	√

Table S4. Quantitative high-resolution XPS spectra (%)

Element	LCC	Modified LCC
C	68.67	75.27
O	31.33	24.73
O/C	0.46	0.33

Table S5. Cls·and Ols peaks of: LCC and·modified LCC

Object	Type	Assignment	LCC	Modified LCC
Ralative content C1s(%)	C1	C-C	25.30	34.82
	C2	C-O	57.03	43.63
	C3	C=O	17.67	21.55
Ralative content O1s(%)	O1	O-C=O	0.00	0.00
	O2	C=O	9.33	12.62
	O3	C-O	90.67	87.38

Table S6. a. Primers used for RT-qPCR in cell.

Gene	Primer sequence	
	Forward primer	Reverse primer
TNF-α	TGCACTTTGGAGTGATCGGC	ACTCGGGGTTCGAGAAGATG
IL-1β	TCGCCAGTGAAATGATGGCT	AGAACACCACTTGTTGCTCCA
IL-10	CCTGCCTAACATGCTTCGAG	TGGCAACCCAGGTAACCCTT
GAPDH	ATTTCTCCTCCGGGTGATGC	TGACGGTGCCATGGAATTTG

Table S6. b. Primers used for RT-qPCR in mice.

Gene	Primer sequence	
	Forward primer	Reverse primer
TNF-α	CCCTCACACTCACAAACCAC	ACAAGGTACAACCCATCGGC
IL-1β	TGCCACCTTTTGACAGTGATG	TGATGTGCTGCTGCGAGATT
IL-10	AAGCTCCAAGACCAAGGTGTC	TCCGTTAGCTAAGATCCCTGG
β-actin	ACAGCAGTTGGTTGGAGCAA	ACGCGACCATCCTCCTCTTA

Table S7. a. Experimental design of $W_1/O/W_2$ for alleviating colitis in mice

Group	0-7 (day)	7-22 (day)	15-22 (day)
Con	Free drinking water	Normal saline	Free drinking water
Emulsion	Free drinking water	Emulsion	Free drinking water
DSS-Emulsion	Free drinking water	Emulsion	Free drinking 3% DSS
DSS-Free	Free drinking water	Free (Q+C)	Free drinking 3% DSS
DSS-embedding	Free drinking water	Embedding(Q+C)	Free drinking 3% DSS

Table S7. b. Disease activity index score

Weight loss (%)	Stool status	Degree of rectal bleeding	Score
0%	Normal	No	0
1%-5%	/	/	1
5%-10%	Loose stool	Mild blood in the stool	2
10-15%	/	Occult blood	3
≥15%	Watery diarrhoea	Obvious blood in the stool	4

Table S7. c. Colonic H&E pathological score

Inflammation	Depth of lesion	Damage to crypts	Extent of disease	Score
Nothing	Nothing	Nothing	Nothing	0
Light	Mucosal layer	1/3	1-25%	1
Moderate	Submucosa	2/3	26-50%	2
Serious	Muscularis	100%	51-75%	3
/	Outer membrane	/	76-100%	4
